# *Calmodulin* and *calmodulin-like* gene family in barley: Identification, characterization and expression analyses

**DOI:** 10.3389/fpls.2022.964888

**Published:** 2022-08-19

**Authors:** Kangfeng Cai, Liuhui Kuang, Wenhao Yue, Shanggeng Xie, Xue Xia, Guoping Zhang, Junmei Wang

**Affiliations:** ^1^Key Laboratory of Digital Dry Land Crops of Zhejiang Province, Zhejiang Academy of Agricultural Sciences, Hangzhou, China; ^2^National Barley Improvement Centre, Hangzhou, China; ^3^Institute of Crop Science, Zhejiang University, Hangzhou, China

**Keywords:** calmodulin, calmodulin-like, barley, expression, pan-genome, abiotic stress

## Abstract

Calmodulin (CaM) and calmodulin-like (CML) proteins are Ca^2+^ relays and play diverse and multiple roles in plant growth, development and stress responses. However, *CaM/CML* gene family has not been identified in barley (*Hordeum vulgare*). In the present study, 5 *HvCaMs* and 80 *HvCMLs* were identified through a genome-wide analysis. All HvCaM proteins possessed 4 EF-hand motifs, whereas HvCMLs contained 1 to 4 EF-hand motifs. *HvCaM2*, *HvCaM3* and *HvCaM5* coded the same polypeptide although they differed in nucleotide sequence, which was identical to the polypeptides coded by *OsCaM1-1*, *OsCaM1-2* and *OsCaM1-3*. *HvCaMs/CMLs* were unevenly distributed over barley 7 chromosomes, and could be phylogenetically classified into 8 groups. *HvCaMs/CMLs* differed in gene structure, *cis*-acting elements and tissue expression patterns. Segmental and tandem duplication were observed among *HvCaMs/CMLs* during evolution. *HvCML16*, *HvCML18*, *HvCML50* and *HvCML78* were dispensable genes and the others were core genes in barley pan-genome. In addition, 14 *HvCaM/CML* genes were selected to examine their responses to salt, osmotic and low potassium stresses by qRT-PCR, and their expression were stress-and time-dependent. These results facilitate our understanding and further functional identification of *HvCaMs/CMLs*.

## Introduction

As sessile organisms, plants are constantly exposed to fluctuating environments and are prone to suffer from abiotic stresses. During evolution, plants have developed sophisticated mechanisms to perceive and respond to environmental cues, in order to maintain normal growth and development. Calcium is an essential element for plants not only as a structural component for cell wall and membrane stability, but also as an important second messenger in signaling networks (Thor, 2019). Upon exposure to environmental stimuli, Ca^2+^ enters into cytosol through Ca^2+^-permeable cation channels from apoplast or intracellular compartments (such as the endoplasmic reticulum and vacuoles), which leads to an increase in cytosolic calcium concentration ([Ca^2+^]_cyt_). Then the cytosolic Ca^2+^ are transported out of cells or compartmented into calcium pools by Ca^2+^-ATPases and H^+^/Ca^2+^ antiporters, and [Ca^2+^]_cyt_ is recovered to resting levels ranging from 50 to 200 nM (Lee and Seo, 2021). Ca^2+^ signatures, defined as spatial and temporal characteristics of transient Ca^2+^ influxes varying in amplitude, frequency, and intracellular distribution, encode unique information of external stimuli (Zhu et al., 2015; Leitão et al., 2019). Ca^2+^-binding proteins (Ca^2+^ sensors) decode, relay and amplify the information of Ca^2+^ signatures, and then regulate downstream signal transduction and initiate specific cellular responses (Zhu et al., 2015; Thor, 2019). Ca^2+^ sensors include calmodulins (CaMs) and CaM-like proteins (CMLs), Ca^2+^-dependent protein kinases (CDPKs or CPKs) and Ca^2+^- and Ca^2+^/CaM-dependent protein kinase (CCaMK), and calcineurin B-like proteins (CBLs; Galon et al., 2010). These proteins contain one or more EF-hand motifs and can bind Ca^2+^ reversibly to provoke conformational changes for binding and regulating downstream targets, and then propagate Ca^2+^ signals (McCormack et al., 2005; Zhu et al., 2015). CDPKs and CCaMK are sensor responders (with Ca^2+^-binding and kinase activities), while CaMs, CMLs and CBLs are sensor relay proteins (with only Ca^2+^-binding ability, except AtCaM7; Reddy et al., 2011; Vadassery et al., 2012). CBLs specifically interact with CBL-interacting protein kinases (CIPKs), whereas CaMs and CMLs interact with diverse proteins (Reddy et al., 2011). CaM is highly conserved during evolution and exists in all eukaryotes, whereas CMLs are restricted to plants and lower protists (Cheval et al., 2013). Generally, CaM proteins consist of 149 amino acid residues and contain 4 typical Ca2^+^-binding EF-hand motifs, while CML proteins considerably vary in length and possess 1 to 6 EF-hand motifs (Aleynova et al., 2020). Both CaMs and CMLs lack any other functional domains or intrinsic activities (Vadassery et al., 2012; Aleynova et al., 2020).

*CaM* and *CML* genes play diverse and multiple roles in plant growth, development and stress responses. Totally, 7 *CaMs* and 50 *CMLs* have been identified in *Arabidopsis thaliana* (McCormack and Braam, 2003; McCormack et al., 2005), and their functions have been analyzed in detail. CaM1 positively regulates ROS production, leaf senescence and ABA response (Dai et al., 2018). Together with CaM4, CaM1 confers salt resistance by promoting NO accumulation (Zhou et al., 2016). CaM4 negatively regulates freezing tolerance by interacting with CaM-binding protein PATL1 (Chu et al., 2018). NO functions as a signal and acts upstream of CaM3 in thermotolerance, and CaM3 inhibits NO accumulation and improves thermotolerance (Xuan et al., 2010; Zhang et al., 2020). CaM7 is a component of photomorphogenesis and multiple signaling pathways are involved in plant immunity, biotic and abiotic stress responses, and hormonal responses (Basu et al., 2021). On the other hand, CML24 plays important roles in pollen germination and pollen tube extension (Yang et al., 2014), circadian oscillation (Martí Ruiz et al., 2018), root mechanoresponses (Wang et al., 2011), Al resistance (Zhu et al., 2022), as well as seed germination, floral transition, ion stress and the sensing of photoperiod (Delk et al., 2005). CML42 participated in trichome morphology (Dobney et al., 2009), insect herbivory defense and drought stress responses (Vadassery et al., 2012; Scholz et al., 2015). CML37, CML38 and CML39 are involved in responses to H_2_O_2_ or MeJA treatments as well as drought, salinity, wounding and bacterial infection (Vanderbeld and Snedden, 2007; Scholz et al., 2015). Interestingly, CML37 and CML42 are proved to act antagonistically in the regulation of induced defense responses, and the double knock-out line, *cml37* × *cml42*, shows wild-type phenotypes upon stresses (Heyer et al., 2021). CML8 and CML9 are implicated in both abiotic and biotic stress responses (Magnan et al., 2008; Zeng et al., 2015; Zhu et al., 2017, 2021). CML20 is a negative regulator in guard cell ABA signaling and drought tolerance (Wu et al., 2017). In rice, 5 *CaMs* and 32 *CMLs* have been identified (Boonburapong and Buaboocha, 2007). *OsCaM1-1* mediates the expression of downstream target genes and contributed to rice thermotolerance (Wu et al., 2012). *OsCML4* and *OsDSR-1* improves drought tolerance of rice through ROS scavenging (Yin et al., 2015, 2017). *OsCML16* plays important roles in *OsERF48* regulation of root growth and drought tolerance (Jung et al., 2017). *OsMSR2*, a novel rice calmodulin-like gene, enhances drought and salt tolerance and increases ABA sensitivity in *Arabidopsis* (Xu et al., 2011). In wheat, overexpression of *TaCML20* enhances water soluble carbohydrate accumulation and yield (Kalaipandian et al., 2019). TaCML36 positively participates in an immune response to *Rhizoctonia cerealis* by modulating the expression of defense-associated genes (Lu et al., 2019). In tomato, SlCML37 interacts with proteasome maturation factor SlUMP1 and plays a role in fruit chilling stress tolerance (Tang et al., 2021). SlCML39 acts negatively in high temperature tolerance possibly through ABA signaling pathway (Ding et al., 2021). Overexpression of *ShCML44* enhances tolerance to cold, drought and salinity stress by higher antioxidant enzymes activity, less membrane damage, better gas exchange and plant water relations in *Solanum habrochaites* (Munir et al., 2016). MtCML40 negatively regulates salt tolerance through targeting MtHKT-dependent Na^+^ accumulation in *Medicago truncatula* (Zhang et al., 2019). MtCML42 positively regulates cold tolerance through regulating *MtCBF1* and *MtCBF4* expression, and regulates flowering time through sequentially downregulation of *MtABI5* and upregulation of *MtFTa1* (Sun et al., 2021). Alfalfa (*Medicago sativa*) MsCML10 regulates cold tolerance through activating MsGSTU8 and MsFBA6, leading to improved maintenance of ROS homeostasis and increased accumulation of sugars for osmoregulation, respectively (Yu et al., 2022). MsCML46 enhances tolerance to abiotic stresses through alleviating osmotic stress and oxidative damage in transgenic tobacco (Du et al., 2021).

Although less well-studied than in *Arabidopsis thaliana* and rice, the *CaM* and *CML* gene family has been characterized in some other plant species, such as *Brassica napus* (He et al., 2020), wheat (Liu et al., 2022), papaya (Ding et al., 2018), wild tomato (Shi and Du, 2020) and grapevine (Vandelle et al., 2018). However, similar research has not been conducted in barley. In the present study, 5 *HvCaM* and 80 *HvCML* genes were identified in barley genome, and their phylogenetic relationships, gene structures, *cis*-acting elements, syntenic relationships, tissue expression patterns and responses to abiotic stresses were analyzed. These results facilitate our understanding and further functional identification of *HvCaMs/CMLs*.

## Materials and methods

### Identification of *CaM*/*CML* genes in barley

The protein sequences of *CaM/CML* genes in *Arabidopsis thaliana* (McCormack and Braam, 2003; McCormack et al., 2005) and rice (Boonburapong and Buaboocha, 2007) were retrieved from TAIR^1^ and RAP-DB,^2^ respectively. Then these protein sequences were used as queries (Blastp, *E*-value <1e-5) to search against barley genome database Morex v2 (Monat et al., 2019a). Meanwhile, ‘IPR002048’ was used as criterion to search against barley genome database in EnsemblPlants.^3^ The putative *HvCaM/CML* genes were further verified using IntertPro database^4^ (Blum et al., 2021), and those containing domains other than ‘EF-hand domain’ were discarded.

### Physicochemical properties and subcellular localizations

The theoretical molecular weights (MW) and isoelectric points (pI) of HvCaMs/CMLs were calculated using ExPASy.^5^ The subcellular localizations of HvCaMs/CMLs were predicted using BUSCA^6^ (Savojardo et al., 2018).

### Phylogenetic and syntenic relationships

The CaM/CML protein sequences of *Arabidopsis* (7 AtCaMs, 50 AtCMLs; McCormack and Braam, 2003; McCormack et al., 2005) and rice (5 OsCaMs, 32 OsCMLs; Boonburapong and Buaboocha, 2007) were downloaded from TAIR and RAP-DB, respectively. CaM1 from *Amborella trichopoda* was set as outgroup. CaM/CML protein sequences of *Arabidopsis*, rice and barley were aligned with MAFFT^7^ (Madeira et al., 2019), and the phylogenetic tree was constructed using MEGA X with maximum-likelihood (ML) method and 1,000 bootstrap replicates (Kumar et al., 2018). The genome sequences and annotations of *Brassica napus* was downloaded from BnPIR,^8^ the genomic data of *Arabidopsis*, rice and wheat were downloaded from EnsemblPlants,^9^ and their syntenic relations with barley were analyzed and visualized using TBtools (Chen et al., 2020).

### Sequence, chromosomal location and duplication analyses

Conserved motifs of *HvCaMs/CMLs* were analyzed using MEME^10^ (Bailey et al., 2009) with following parameters: classic motif discovery mode, any number of repetitions (anr), motif number was set to 10. Number of EF-hand and calcium binding bites were analyzed using InterPro database (see Footnote 4; Blum et al., 2021). Motifs, gene structures and gene duplication events were visualized using TBtools (Chen et al., 2020).

### *Cis*-acting elements

The 2000 bp upstream of coding sequences of *HvCaMs/CMLs* were extracted for *cis*-acting element analysis using PlantCARE database^11^ (Lescot et al., 2002).

### Tissue expression patterns

Transcriptomic data (FPKM) were downloaded from BARLEX^12^ and normalized with log_10_(FPKM+1) transform. The expression heatmap of different tissues was drawn using TBtools (Chen et al., 2020).

### *HvCaMs/CMLs* in pan-genome context

Assemblies of 20 barley genotypes were downloaded (Jayakodi et al., 2020), and nucleotide sequences of 85 *HvCaM/CML* genes were used as queries to search against each assembly. The Blastn results were verified for presence/absence variation and chromosomal location.

### Plant materials, growth conditions and abiotic stress treatments

Barley (*Hordeum vulgare* cv. Golden Promise) seeds were sterilized with 10% commercial NaClO, rinsed with tap water and then germinated in BSM solution (0.5 mM KCl + 0.1 mM CaCl_2_) for 2 days. Afterwards BSM was changed to 1/5 Hoagland solution for another 5 days with a photoperiod of 14/10 h, light intensity of 200 ± 25 μmol·m^−2^·s^−1^, temperature of 23/18°C (day/night) and relative humidity of 60% (Cai et al., 2019). After growth for 7 days, seedlings were subjected to salt stress (200 mM NaCl), osmotic stress (20% PEG8000) and potassium deficiency (0.01 mM K^+^; Cai et al., 2021). Plants grown in 1/5 Hoagland solution were set as control. Solutions were renewed every 2 days. RNA extraction and qRT-PCR were performed on barley roots under both control and abiotic stress conditions after treatment for 1 h, 3 h, 6 h, 1 day, 3 days, and 6 days. All samples were collected in three replicates.

### qRT-PCR

Total RNA was extracted using Easy Plant RNA Extraction Kit (DR0406050, Easy-Do, China). The cDNA was synthesized from total RNA using PrimeScript RT Master Mix (RR036A, TaKaRa, Japan) and was then used for qRT-PCR amplification. qRT-PCR was performed with LightCycler 480 II (Roche, Basel, Switzerland) using ChamQ Universal SYBR qPCR Master Mix (Q711, Vazyme, China). The relative gene expression was calculated with 2^−△△CT^ method (Livak and Schmittgen, 2001) using *actin* as the internal standard. The primer sequences were listed in Supplementary File 1.

## Results

### Identification of *CaM/CML* genes in barley

A total of 5 *HvCaM* and 80 *HvCML* genes were identified in barley and were named in the order of their chromosomal locations (*viz. HvCaM1* to *HvCaM5* and *HvCML1* to *HvCML80*), respectively (Table 1). All HvCaMs shared the same protein length (149 aa), methionine percentage (6.0%), number of introns (1) and EF-hands with calcium-binding ability (4), as well as subcellular localization (nucleus). Their isoelectric points (pI) and theoretical molecular weights (MW) ranged from 4.10 to 4.12 and 16.80 to 16.85 kDa, respectively (Table 1). Notably, *HvCaM2/3/5* differed in nucleotide sequence but coded the same peptide (Table 1; Supplementary File 2). The protein sequence identity of *HvCaM1* and *HvCaM4* to *HvCaM2* were also as high as 99.3 and 98.7%, respectively (Table 1). Compared with HvCaMs, characteristics of HvCMLs were more diverged. The protein length, pI, MW and methionine percentage of HvCMLs ranged from 78 to 389 aa, 3.93 to 9.59, 8.62 to 44.82 kDa and 1.23 to 8.97%, respectively (Table 1). Twenty-three HvCMLs contained 3 EF-hand domains, followed by 20, 19 and 18 HvCMLs containing 1, 4 and 2 domains, respectively. All HvCMLs retained at least one EF-hand domain with calcium-binding ability except HvCML57 and HvCML68, whose sole EF-hand has lost the ability to bind calcium (Table 1; Supplementary File 3). Most of HvCMLs (53, 66.3%) did not contain introns, and the others had 1 to 9 introns. Likewise, most of the HvCMLs (64, 80.0%) were localized in nucleus, followed by 9 and 3 HvCMLs were in chloroplast and endomembrane system, respectively. HvCML24, HvCML37, HvCML45 and HvCML65 were localized in cytoplasm, extracellular space, plasma membrane and chloroplast outer membrane, respectively (Table 1). Sequence identity of all HvCMLs to HvCaM2 were lower than 50% except 6 HvCMLs (HvCML30, HvCML48, HvCML38, HvCML59, HvCML44 and HvCML43).

**Table 1:** Characteristics of 5 *CaMs* and 80 *CMLs* in barley.

Name	Gene ID	EF hands	Intron	pI	MW (kDa)	Length (aa)	SL	Met (%)	Identity to HvCaM2 (%)
HvCaM1	HORVU.MOREX.r2.1HG0056080.1	4	1	4.12	16.85	149	N	9 (6.0)	99.3
HvCaM2	HORVU.MOREX.r2.2HG0090830.1	4	1	4.11	16.83	149	N	9 (6.0)	100
HvCaM3	HORVU.MOREX.r2.3HG0212490.1	4	1	4.11	16.83	149	N	9 (6.0)	100
HvCaM4	HORVU.MOREX.r2.3HG0214120.1	4	1	4.10	16.80	149	N	9 (6.0)	98.7
HvCaM5	HORVU.MOREX.r2.4HG0316710.1	4	1	4.11	16.83	149	N	9 (6.0)	100
HvCML1	HORVU.MOREX.r2.1HG0015960.1	3	3	5.17	35.83	315	N	8 (2.5)	22.6
HvCML2	HORVU.MOREX.r2.1HG0025060.1	4	1	5.17	24.57	228	N	13 (5.7)	38.4
HvCML3	HORVU.MOREX.r2.1HG0041720.1	4	0	4.29	20.96	192	N	3 (1.6)	42.8
HvCML4	HORVU.MOREX.r2.1HG0046990.1	4	0	5.07	20.09	189	C	8 (4.2)	40.3
HvCML5	HORVU.MOREX.r2.1HG0056090.1	3	0	4.53	16.12	151	N	3 (2.0)	44.7
HvCML6	HORVU.MOREX.r2.1HG0062780.1	2	0	4.36	16.52	147	N	8 (5.4)	31.8
HvCML7	HORVU.MOREX.r2.1HG0066020.1	3	1	4.57	21.17	192	N	3 (1.6)	40.1
HvCML8	HORVU.MOREX.r2.1HG0071870.1	1	0	4.43	20.58	182	N	8 (4.4)	24.7
HvCML9	HORVU.MOREX.r2.1HG0075300.1	4	0	6.65	25.36	235	C	7 (3.0)	39.9
HvCML10	HORVU.MOREX.r2.1HG0077590.1	2	0	4.43	20.89	186	EN	11 (5.9)	23.0
HvCML11	HORVU.MOREX.r2.1HG0077610.1	2	0	4.33	20.77	186	EN	8 (4.3)	35.4
HvCML12	HORVU.MOREX.r2.2HG0091700.1	3	0	4.45	20.33	191	N	7 (3.7)	32.7
HvCML13	HORVU.MOREX.r2.2HG0100710.1	1	0	4.42	22.03	207	N	6 (2.9)	19.1
HvCML14	HORVU.MOREX.r2.2HG0100730.1	1	0	4.85	21.46	202	C	6 (3.0)	22.8
HvCML15	HORVU.MOREX.r2.2HG0102600.1	1	0	9.59	10.58	92	N	2 (2.2)	32.2
HvCML16	HORVU.MOREX.r2.2HG0102640.1	2	0	9.58	10.26	89	N	2 (2.3)	26.9
HvCML17	HORVU.MOREX.r2.2HG0102660.1	2	0	9.50	10.47	91	N	4 (4.4)	33.3
HvCML18	HORVU.MOREX.r2.2HG0102680.1	4	6	4.76	19.40	169	N	10 (5.9)	43.6
HvCML19	HORVU.MOREX.r2.2HG0121540.1	3	1	4.65	12.48	111	N	4 (3.6)	34.0
HvCML20	HORVU.MOREX.r2.2HG0146960.1	4	0	4.50	26.43	251	N	9 (3.6)	35.9
HvCML21	HORVU.MOREX.r2.2HG0152560.1	1	5	5.13	34.70	321	N	5 (1.6)	29.8
HvCML22	HORVU.MOREX.r2.2HG0154830.1	1	1	4.68	16.50	150	N	2 (1.3)	29.5
HvCML23	HORVU.MOREX.r2.2HG0157810.1	1	4	4.95	41.04	376	N	11 (2.9)	15.6
HvCML24	HORVU.MOREX.r2.2HG0160400.1	1	3	6.84	19.39	172	CP	3 (1.7)	18.8
HvCML25	HORVU.MOREX.r2.3HG0187650.1	4	0	4.43	19.64	186	N	6 (3.2)	37.9
HvCML26	HORVU.MOREX.r2.3HG0221410.1	4	5	5.11	44.82	389	N	6 (1.5)	27.3
HvCML27	HORVU.MOREX.r2.3HG0223920.1	4	0	5.84	27.80	255	N	9 (3.5)	40.4
HvCML28	HORVU.MOREX.r2.3HG0242210.1	2	0	4.96	21.80	202	C	9 (4.5)	32.2
HvCML29	HORVU.MOREX.r2.3HG0242550.1	3	0	4.31	16.05	145	N	13 (9.0)	35.2
HvCML30	HORVU.MOREX.r2.3HG0246380.1	4	2	4.49	20.51	184	N	9 (4.9)	92.6
HvCML31	HORVU.MOREX.r2.3HG0266990.1	2	0	4.69	20.07	191	C	8 (4.2)	35.6
HvCML32	HORVU.MOREX.r2.3HG0267050.1	4	0	4.79	20.40	191	N	9 (4.7)	44.3
HvCML33	HORVU.MOREX.r2.3HG0268840.1	3	0	4.24	15.93	148	N	9 (6.1)	32.9
HvCML34	HORVU.MOREX.r2.3HG0268890.1	3	0	4.19	16.21	152	N	8 (5.3)	32.0
HvCML35	HORVU.MOREX.r2.3HG0268900.1	3	0	4.14	16.33	152	N	8 (5.3)	32.0
HvCML36	HORVU.MOREX.r2.3HG0268910.1	3	0	4.42	16.25	147	N	8 (5.4)	32.4
HvCML37	HORVU.MOREX.r2.4HG0297110.1	2	4	6.31	33.88	326	EXT	4 (1.2)	21.1
HvCML38	HORVU.MOREX.r2.4HG0297400.1	4	3	4.76	26.33	232	N	11 (4.7)	71.6
HvCML39	HORVU.MOREX.r2.4HG0315500.1	3	0	4.45	20.61	193	N	6 (3.1)	34.1
HvCML40	HORVU.MOREX.r2.4HG0317560.1	3	0	4.05	17.45	167	N	7 (4.2)	25.8
HvCML41	HORVU.MOREX.r2.4HG0325840.1	1	4	4.99	41.97	388	N	8 (2.1)	15.6
HvCML42	HORVU.MOREX.r2.4HG0341420.1	1	9	5.42	19.20	169	N	5 (3.0)	21.7
HvCML43	HORVU.MOREX.r2.5HG0359840.1	4	0	4.16	18.16	165	N	9 (5.5)	64.1
HvCML44	HORVU.MOREX.r2.5HG0359860.1	4	0	4.14	18.25	164	N	9 (5.5)	64.7
HvCML45	HORVU.MOREX.r2.5HG0363320.1	2	6	5.32	36.83	328	PM	9 (2.7)	24.6
HvCML46	HORVU.MOREX.r2.5HG0381760.1	2	0	4.51	19.10	183	N	7 (3.8)	28.8
HvCML47	HORVU.MOREX.r2.5HG0381860.1	2	4	8.60	40.30	389	C	5 (1.3)	20.5
HvCML48	HORVU.MOREX.r2.5HG0382630.1	4	2	4.42	19.92	180	N	8 (4.4)	73.2
HvCML49	HORVU.MOREX.r2.5HG0385590.1	3	0	4.27	17.30	163	N	9 (5.5)	34.6
HvCML50	HORVU.MOREX.r2.5HG0401310.1	2	0	3.93	18.67	182	N	7 (3.9)	37.5
HvCML51	HORVU.MOREX.r2.5HG0401670.1	2	0	4.51	16.49	148	N	9 (6.1)	37.9
HvCML52	HORVU.MOREX.r2.5HG0403070.1	2	0	6.33	21.23	183	C	14 (7.7)	25.3
HvCML53	HORVU.MOREX.r2.5HG0403080.1	1	0	8.61	11.79	103	N	4 (3.9)	31.5
HvCML54	HORVU.MOREX.r2.5HG0403090.1	1	0	5.14	11.05	96	N	3 (3.1)	29.4
HvCML55	HORVU.MOREX.r2.5HG0406420.1	1	0	4.29	14.90	138	N	6 (4.4)	21.2
HvCML56	HORVU.MOREX.r2.5HG0406440.1	1	0	4.41	15.14	137	N	7 (5.1)	19.2
HvCML57	HORVU.MOREX.r2.5HG0407200.1	1	5	4.54	20.10	175	N	6 (3.4)	20.1
HvCML58	HORVU.MOREX.r2.5HG0417090.1	3	1	4.52	17.12	159	N	8 (5.0)	36.6
HvCML59	HORVU.MOREX.r2.5HG0423440.1	4	3	4.04	17.39	155	N	9 (5.8)	67.8
HvCML60	HORVU.MOREX.r2.5HG0426500.1	2	0	6.15	20.25	189	C	6 (3.2)	34.0
HvCML61	HORVU.MOREX.r2.5HG0434020.1	2	0	4.36	16.77	159	N	5 (3.1)	22.1
HvCML62	HORVU.MOREX.r2.5HG0436020.1	1	0	4.49	21.18	206	N	9 (4.4)	33.7
HvCML63	HORVU.MOREX.r2.6HG0474900.1	3	3	4.64	29.22	254	C	9 (3.5)	25.3
HvCML64	HORVU.MOREX.r2.6HG0481250.1	4	5	4.82	42.62	378	EN	6 (1.6)	27.1
HvCML65	HORVU.MOREX.r2.6HG0508160.1	3	0	4.49	18.19	169	COM	6 (3.6)	35.5
HvCML66	HORVU.MOREX.r2.6HG0510800.1	3	0	4.75	14.27	133	N	3 (2.3)	31.6
HvCML67	HORVU.MOREX.r2.6HG0512400.1	1	0	4.71	11.45	98	N	3 (3.1)	24.1
HvCML68	HORVU.MOREX.r2.6HG0519400.1	1	5	4.54	19.96	175	N	5 (2.9)	20.1
HvCML69	HORVU.MOREX.r2.7HG0532820.1	4	0	4.54	19.57	175	N	10 (5.7)	36.0
HvCML70	HORVU.MOREX.r2.7HG0544910.1	2	4	6.52	26.66	246	N	5 (2.0)	21.1
HvCML71	HORVU.MOREX.r2.7HG0550710.1	1	0	9.38	11.05	96	N	2 (2.1)	25.3
HvCML72	HORVU.MOREX.r2.7HG0550720.1	1	0	6.57	10.91	96	N	2 (2.1)	24.1
HvCML73	HORVU.MOREX.r2.7HG0572780.1	2	0	4.19	8.62	78	N	5 (6.4)	36.7
HvCML74	HORVU.MOREX.r2.7HG0581800.1	3	0	4.39	21.05	198	N	5 (2.5)	32.5
HvCML75	HORVU.MOREX.r2.7HG0584210.1	3	0	4.89	16.67	148	N	4 (2.7)	47.7
HvCML76	HORVU.MOREX.r2.7HG0597770.1	3	3	4.73	25.59	224	N	12 (5.4)	21.5
HvCML77	HORVU.MOREX.r2.7HG0605630.1	3	0	3.96	17.54	168	N	6 (3.6)	34.9
HvCML78	HORVU.MOREX.r2.7HG0615610.1	4	4	4.73	20.61	181	N	13 (7.2)	45.6
HvCML79	HORVU.MOREX.r2.7HG0617740.1	3	0	4.27	15.99	148	N	7 (4.7)	33.1
HvCML80	HORVU.MOREX.r2.7HG0617750.1	3	0	4.28	15.89	147	N	8 (5.4)	34.0

aa, Amino acids; pI, Isoelectric point; MW, Molecular weight; SL, Subcellular localization; N, Nucleus; C, Chloroplast; EN, Endomembrane system; CP, Cytoplasm; EXT, Extracellular space; PM, Plasma membrane; COM, chloroplast outer membrane; Met, Methionine.

### Phylogenetic and gene structure analysis of *HvCaMs/CMLs*

**Figure 1:**
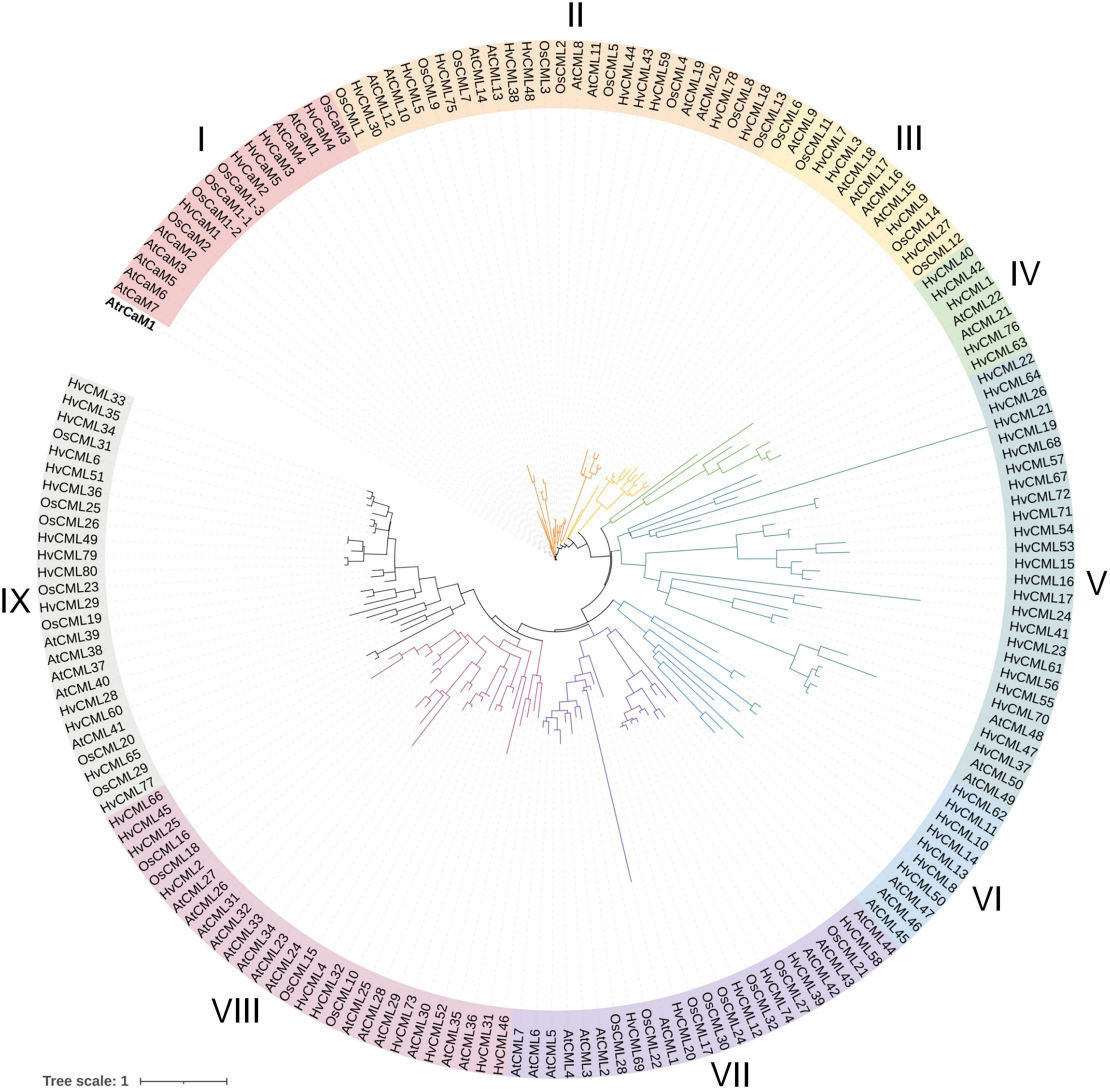
Phylogenetic tree of *CaM* and *CML* family proteins from barley, *Arabidopsis* and rice. *CaM1* from *Amborella trichopoda* was used as outgroup. Atr, *Amborella trichopoda*; Hv, *Hordeum vulgare*; Os, *Oryza sativa*; At, *Arabidopsis thaliana*.

The protein sequence of 7 *AtCaMs* and 50 *AtCMLs* in *Arabidopsis*, 5 *OsCaMs* and 32 *OsCMLs* in rice, 5 *HvCaMs* and 80 *HvCMLs* in barley, together with *CaM1* from *Amborella trichopoda* (outgroup), were used to construct phylogenetic tree (Figure 1). *CaMs/CMLs* were grouped into 9 clusters according to their phylogenetic relationships. Cluster I comprised 17 *CaM* members. Cluster VIII contained 28 members and 10 of them were *HvCMLs*. Cluster II and V each contained 27 *CML* genes (with 10 and 24 *HvCMLs*, respectively). Cluster IX and VII contained 26 and 24 *CMLs*, respectively (with 14 and 6 *HvCMLs*, respectively). Cluster III, VI and IV consisted of 13, 10 and 7 *CML* genes, respectively (*HvCMLs* in each cluster being 4, 7, and 5).

**Figure 2:**
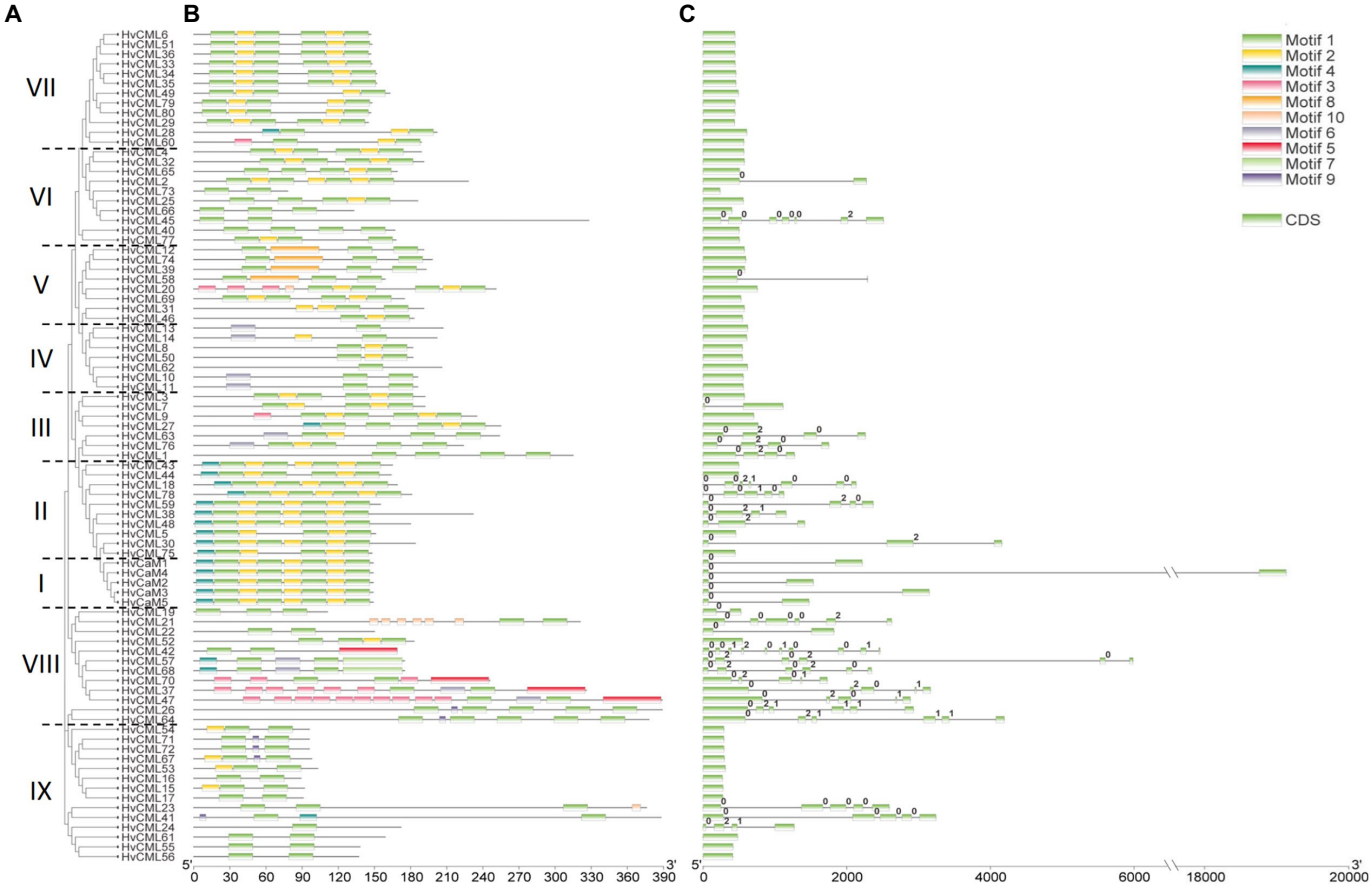
Phylogenetic relationship and sequence characteristics of *HvCaMs/CMLs*. **(A)** Phylogenetic tree of HvCaM/CML proteins; **(B)** Conserved motifs of HvCaM/CML proteins; **(C)** Gene structure of *HvCaMs/CMLs*.

Phylogeny, conserved motifs and gene structures of *HvCaMs/CMLs* were further comprehensively analyzed (Figure 2). *HvCaMs/CMLs* were classified into 9 groups (Figure 2A). Group I consisted of 5 *HvCaMs*, all of which had one phase 0 intron and the same conserved motif arrangements (Figure 2). Group IX incorporated the most *HvCML* genes (14), followed by group VII and VIII with 12 *HvCML* members in each. Group II and VI each contained 10 *HvCMLs*, and group III, IV and V contained 7, 7 and 8 genes, respectively. *HvCMLs* with close phylogenetic relatives showed similar motif arrangements, though considerable variation was observed between genes from group V and VIII (Figure 2B). More than half genes from group VIII, II and III were intron-rich (91.7, 60.0 and 57.1%, respectively), followed by group IX, VI and V, in which the percentage was 21.4, 20.0 and 12.5%, respectively (Figure 2C). However, all *HvCMLs* in group IV and VII were intron-free (Figures 2A,C). Overall, *HvCaMs* were more conserved than *HvCMLs* in phylogeny and gene structure (Figures 1, 2; Table 1).

### Chromosomal distribution and duplication analysis of *HvCaMs/CMLs*

**Figure 3:**
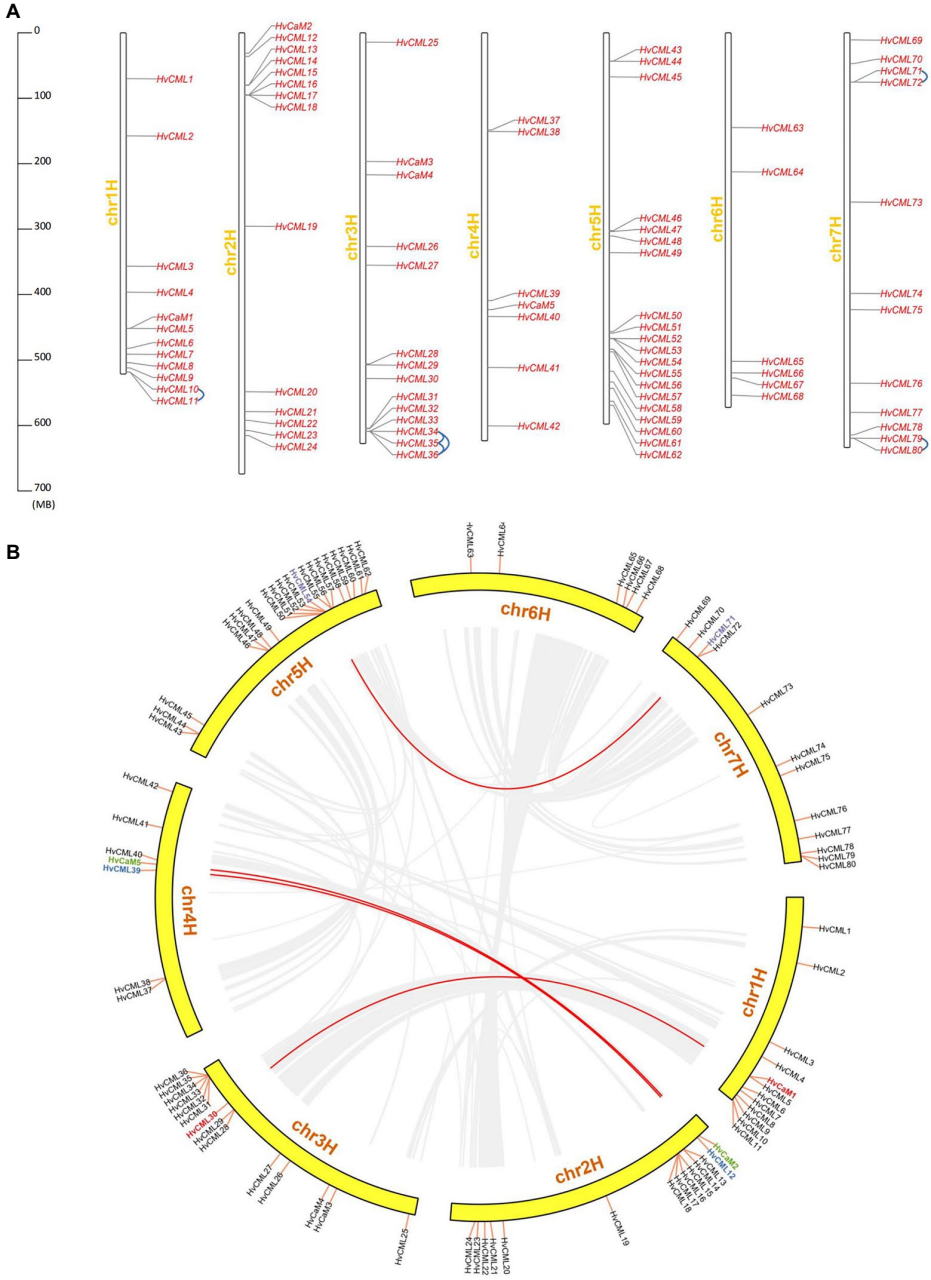
Chromosomal distribution and duplication of *HvCaMs/CMLs*. **(A)** Chromosomal distribution and tandem duplication of *HvCaMs/CMLs*; **(B)** Segmental duplication of *HvCaMs/CMLs*.

*HvCaMs/CMLs* were unevenly distributed over 7 chromosomes, with chromosome 5 containing 20 *HvCML* genes (*HvCML43* to *HvCML62*), chromosome 2 (*HvCaM2*, *HvCML12* to *HvCML24*) and chromosome 3 (*HvCaM3* to *HvCaM4*, *HvCML25* to *HvCML36*) each containing 14 genes, chromosome 1 (*HvCaM1*, *HvCML1* to *HvCML11*) and chromosome 7 (*HvCML69* to *HvCML80*) each containing 12 genes, and chromosome 4 (*HvCaM5*, *HvCML37* to *HvCML42*) and chromosome 6 (*HvCML63* to *HvCML68*) containing 7 and 6 genes, respectively (Figure 3A).

Tandem and segmental duplications are considered as the main driving forces expanding gene families during evolution (Kuo et al., 2019). In this study, tandem duplicated *HvCML* genes were defined as follows: (1) on a single chromosome with no more than one intervening gene; (2) similarity of aligned regions more than 70% (Zhu et al., 2014; Vatansever et al., 2016). In total, 9 genes (6 pairs) were involved in tandem duplication events (*HvCML10* and *HvCML11*, *HvCML34* and *HvCML35*, *HvCML35* and *HvCML36*, *HvCML34* and *HvCML36*, *HvCML71* and *HvCML72*, *HvCML79* and *HvCML80*; Figure 3A). On the other hand, 8 genes (4 pairs) were implicated in segmental duplication events (*HvCaM1* and *HvCML30*, *HvCaM2* and *HvCaM5*, *HvCML12* and *HvCML39*, *HvCML54* and *HvCML71*; Figure 3B).

### *Cis*-acting elements analysis

**Figure 4:**
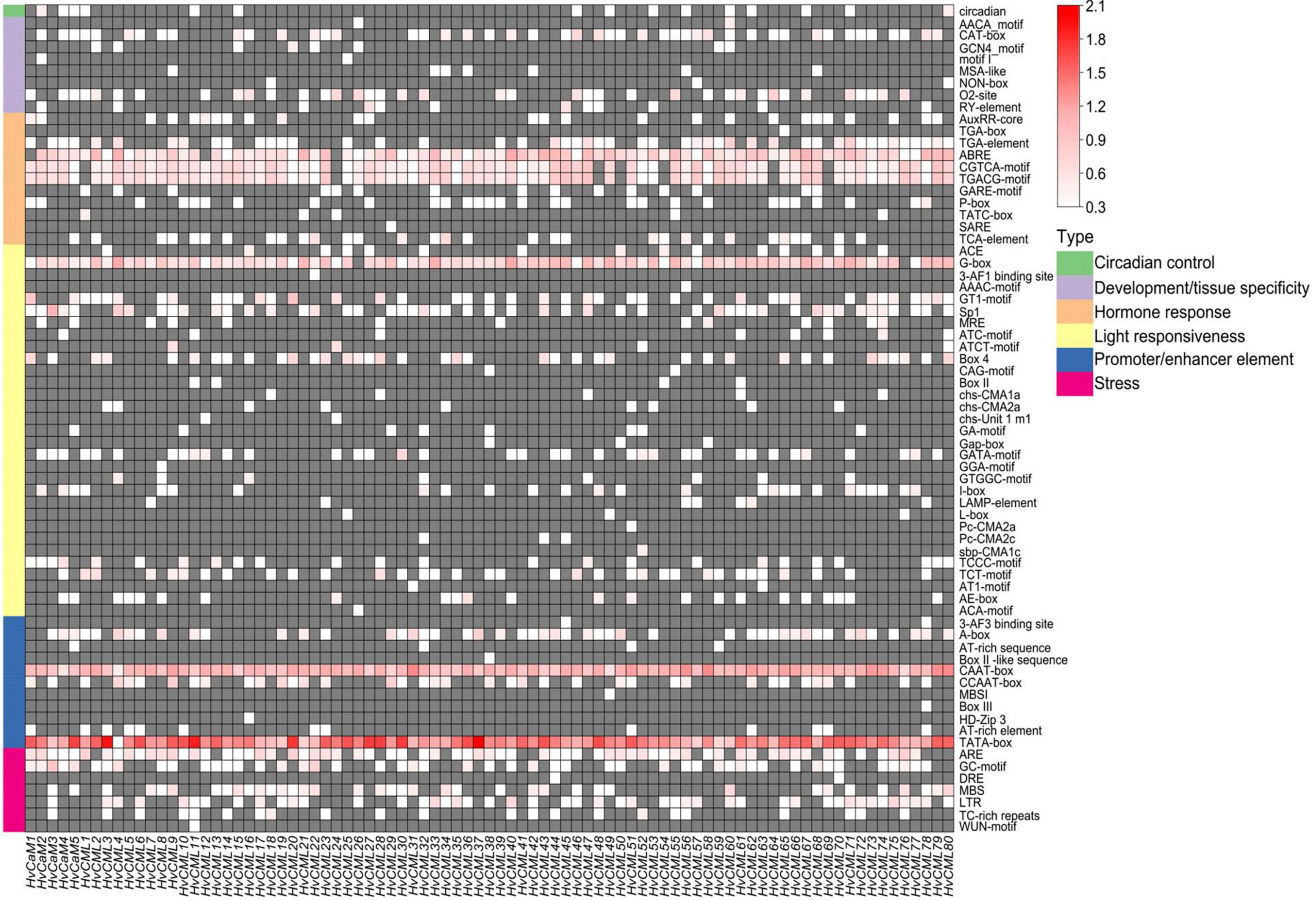
*Cis*-acting elements in promoter regions of *HvCaMs/CMLs*. *Cis*-acting elements were predicted based on 2 kb sequences upstream of coding sequences of *HvCaMs/CMLs*. The quantity of *cis*-acting elements was normalized by log_10_(number + 1) and then used for heatmap construction.

The 2 kb upstream sequences of *HvCaMs/CMLs* coding regions were retrieved for *cis*-acting elements analysis. Totally, 69 *cis*-acting elements were identified and could be fractionalized into 6 types based on functional annotations (Figure 4; Supplementary File 4). Nearly half of the *cis*-acting elements (31, 44.9%) were in ‘light responsiveness’ category, followed by ones in ‘hormone response’ (11, 15.9%), ‘promoter/enhancer element’ (11, 15.9%), ‘development/tissue specificity’ (8, 11.6%) and ‘stress’ category (7, 10.1%). Only one kind of elements was identified in ‘circadian control’ category (Figure 4). CAAT-box and TATA-box in ‘promoter/enhancer element’ category, which are binding sites of RNA polymerase and responsible for transcription efficiency, were ubiquitously detected in all *HvCaMs/CMLs*, indicating their potential critical roles in controlling the transcription initiation and expression levels of *HvCaMs/CMLs*. ABRE, CGTCA-motif and TGACG-motif (in ‘hormone response’ category), which are involved in ABA and methyl jasmonate (MeJA) responsiveness, respectively, were widely present in *HvCaMs/CMLs* (Figure 4). Further, G-box in ‘light responsiveness’ category was also extensively distributed in promoter regions of *HvCaMs/CMLs* (Figure 4). On the other hand, the presence of other *cis*-acting elements was comparatively gene-specific. These results suggest that *HvCaMs/CMLs* might play pivotal roles in hormone metabolism and environmental responses, while the response patterns and expression profiling of *HvCaMs/CMLs* might be different.

### Synteny analysis of *HvCaMs/CMLs*

**Figure 5:**
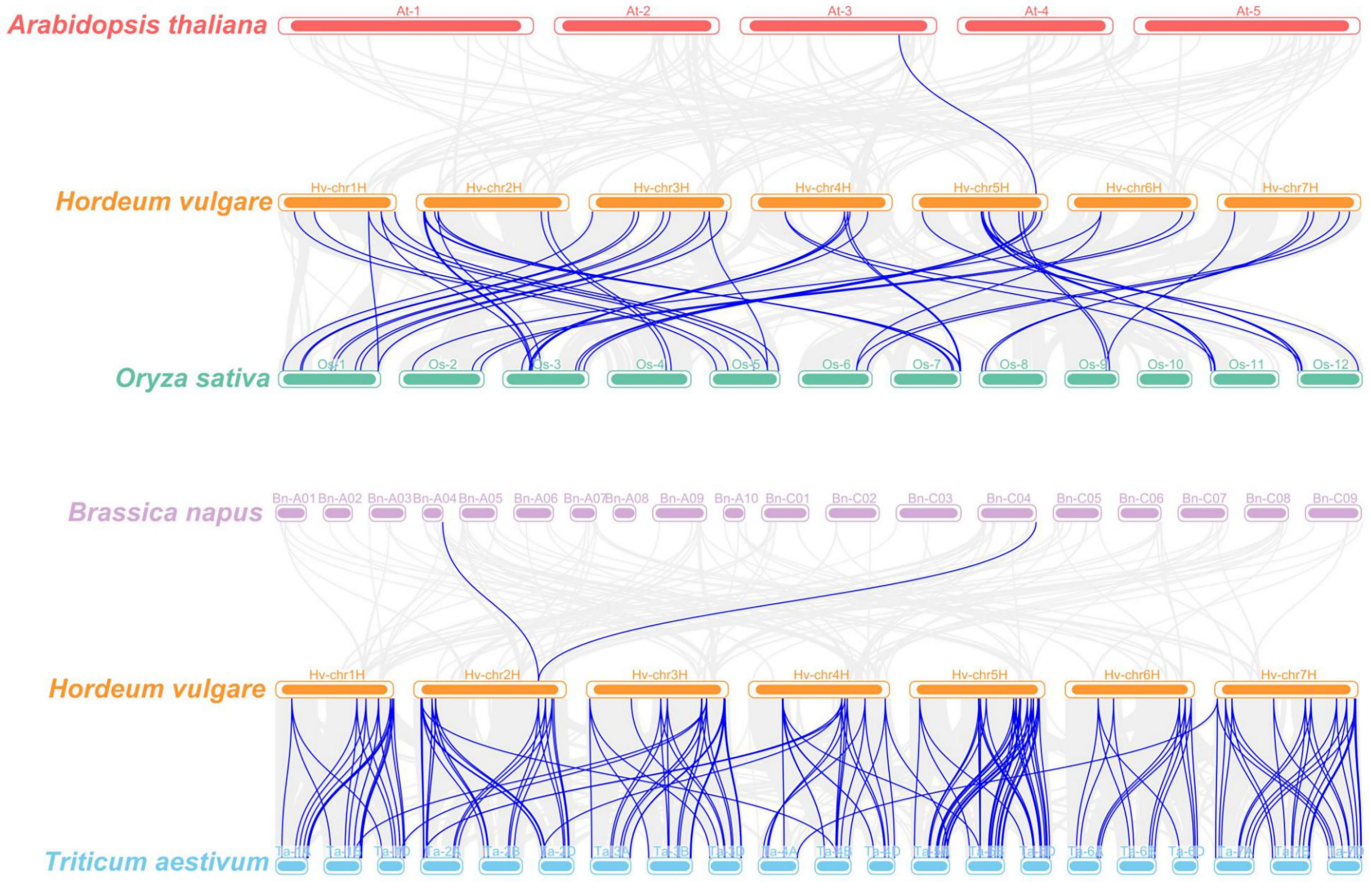
Synteny analyses of *CaMs/CMLs* between barley and four plant species (*Arabidopsis thaliana*, *Oryza sativa*, *Brassica napus*, and *Triticum aestivum*). Gray lines indicated collinear blocks and blue lines highlighted syntenic *CaMs/CMLs* gene pairs.

The syntenic relationships of *CaMs/CMLs* between barley and four plant species were investigated (Figure 5; Supplementary File 5). Only one pair of orthologous genes was identified between *Arabidopsis* and barley (*AtCML41* and *HvCML60*). Similarly, only two pair of orthologous genes was identified between *Brassica napus* and barley (Supplementary File 5). This might be caused by evolutionally far genetic relationships between dicots and monocots. However, 60 pairs of orthologous genes were observed between barley and rice, involving 46 *HvCaMs/CMLs* (5 *HvCaMs* and 41 *HvCMLs*) and 49 rice genes (including 5 *OsCaMs*, 21 *OsCMLs* and 23 ‘other genes’). Nine *HvCaMs/CMLs* (3 *HvCaMs* and 6 *HvCMLs*) each had 2 orthologous genes in rice, while 5 *OsCaMs/CMLs* (2 *OsCaMs* and 3 *OsCMLs*) each had 2 orthologous genes in barley. Besides, *OsCaM1-1* was orthologous to 3 *HvCaM* genes (*HvCaM1*, *HvCaM2* and *HvCaM5*). Wheat was evolutionally closer to barley than rice, as expected, much more orthologous gene pairs (189 pairs) were detected between barley and wheat (Supplementary File 5).

### *HvCaMs/CMLs* in different genotypes

**Figure 6:**
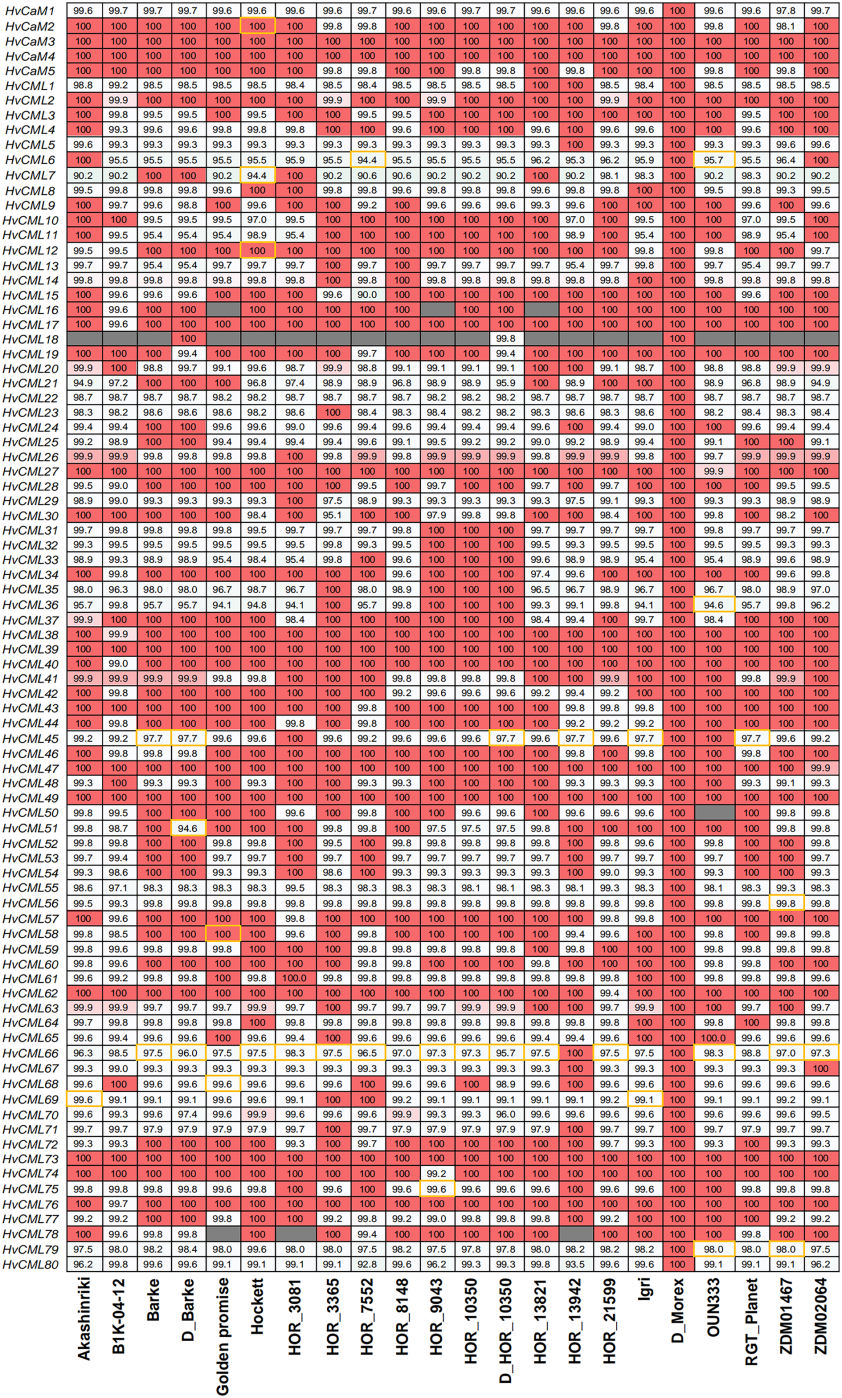
Nucleotide sequence identity of *HvCaMs/CMLs* between barley genotypes. Sequences of *HvCaMs/CMLs* in Morex were used as Blastn queries and the percent identity was designated in grids. Grids filled with gray indicated gene absence variation and those framed with yellow indicated discrepancies of chromosomal location between query and best hit.

Pan-genome refers to a species-wide catalog of genic presence/absence variation or structural variation that affects (potentially non-coding) sequences of 50 or more base pairs in size (Jayakodi et al., 2020). The genic presence/absence variation of *HvCaMs/CMLs* was investigated in the context of the first-generation barley pan-genome comprising 20 varieties (Figure 6; Supplementary File 6; Jayakodi et al., 2020). The pan-genome can be divided into core genome and dispensable genome, the former comprises genes present in all genotypes while the latter comprises genes absent from some genotypes, which refers to genes showing presence/absence variation (Tettelin et al., 2005; Monat et al., 2019b). Accordingly, 81 of all 85 *HvCaMs/CMLs* (95.3%) were in the category of core genome and only 4 genes (*HvCML16*, *HvCML18*, *HvCML50* and *HvCML78*) were in dispensable-genome category. B1K-04-12 was a wild barley genotype and displayed the least members (19 *HvCaMs/CMLs*) identical in nucleotide sequences to Morex (Figure 6). In the other 18 genotypes, 29 to 46 *HvCaMs/CMLs* were identical to those in Morex. Two *HvCaM*s (*HvCaM3* and *HvCaM4*) and 3 *HvCMLs* (*HvCML39*, *HvCML49* and *HvCML73*) were identical in nucleotide sequence among 20 genotypes. However, sequences of *HvCaM1* and 6 *HvCMLs* (*HvCML22*, *HvCML55*, *HvCML56*, *HvCML70*, *HvCML79* and *HvCML80*) in reference barley cultivar Morex differed from those in all the other 19 genotypes. Furthermore, the sequence identity of *HvCaMs* and *HvCMLs* between reference cultivar Morex and the other 19 genotypes ranged from 97.8 to 100% and from 90.0 to 100%, respectively. The biggest difference was in *HvCML7* and *HvCML6*, whose sequence identity between Morex and the other genotypes averaged 93.7 and 96.0%, respectively.

### Tissue expression patterns of *HvCaMs/CMLs*

**Figure 7:**
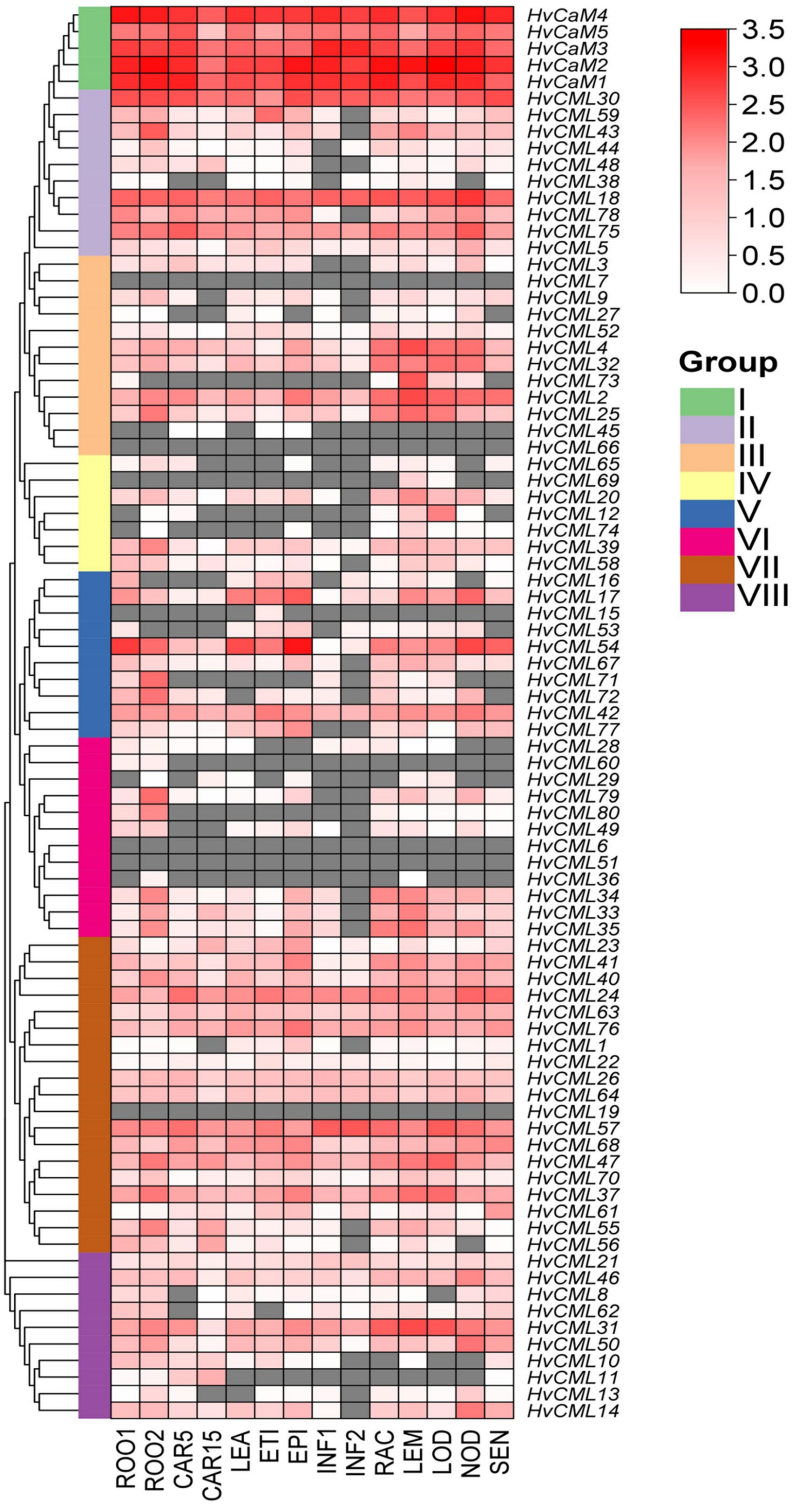
Expression profiling of *HvCaMs/CMLs* in 14 tissues based on transcriptomic data. FPKM values were normalized by log_10_(FPKM+1) transformation. ROO1, roots from seedlings (10 cm shoot stage); ROO2, roots (28 DAP); CAR5, developing grain (5 DAP); CAR15, developing grain (15 DAP); LEA, shoots from seedlings (10 cm shoot stage); ETI, etiolated seedling, dark condition (10 DAP); EPI, epidermal strips (28 DAP); INF1, young developing inflorescences (5 mm); INF2, developing inflorescences (1–1.5 cm); RAC, inflorescences, rachis (35 DAP); LEM, inflorescences, lemma (42 DAP); LOD, inflorescences, lodicule (42 DAP); NOD, developing tillers, 3rd internode (42 DAP); SEN, senescing leaves (56 DAP).

Transcriptomic data of *HvCaMs/CMLs* were downloaded from BARLEX database to analyze their expression patterns in 14 tissues (Figure 7; Supplementary File 7). Five *HvCaMs* in group I ubiquitously expressed in all 14 tissues with relatively high levels. Most genes in group VII (15/19, 79.0%) also expressed in all tissues, although with comparatively low levels. Besides, there were 4 genes in group II, 5 genes in group III, 1 gene in group IV, 3 genes in group V and 4 genes in group VIII showing ubiquitous expression. Notably, the expression of 5 genes (*HvCML7* and *HvCML66* in group III, *HvCML6* and *HvCML51* in group 6 and *HvCML19* in group VII) failed to be detected in any determined tissues under normal conditions, indicating that these genes might be luxury genes. Expression of the rest 43 (50.6%) genes could be detected in at least one tissues, showing high ratio of tissue-specific genes in *HvCaM/CML* gene family. Furthermore, 3 *HvCMLs* (*HvCML73*, *HvCML16* and *HvCML53*) expressed in seedling roots but not in roots at 28 days after pollination. On the contrary, 4 genes (*HvCML12*, *HvCML74*, *HvCML29* and *HvCML36*) were silent in roots at seedling stage but expressed at 28 days after pollination. Similar situation was also observed for 8 *HvCMLs* in developing grains at 5 days or 15 days after pollination. Thus, the expression of *HvCMLs* is not only tissue-specific but also development phase-dependent.

### Expression of *HvCaMs/CMLs* in response to abiotic stresses

**Figure 8:**
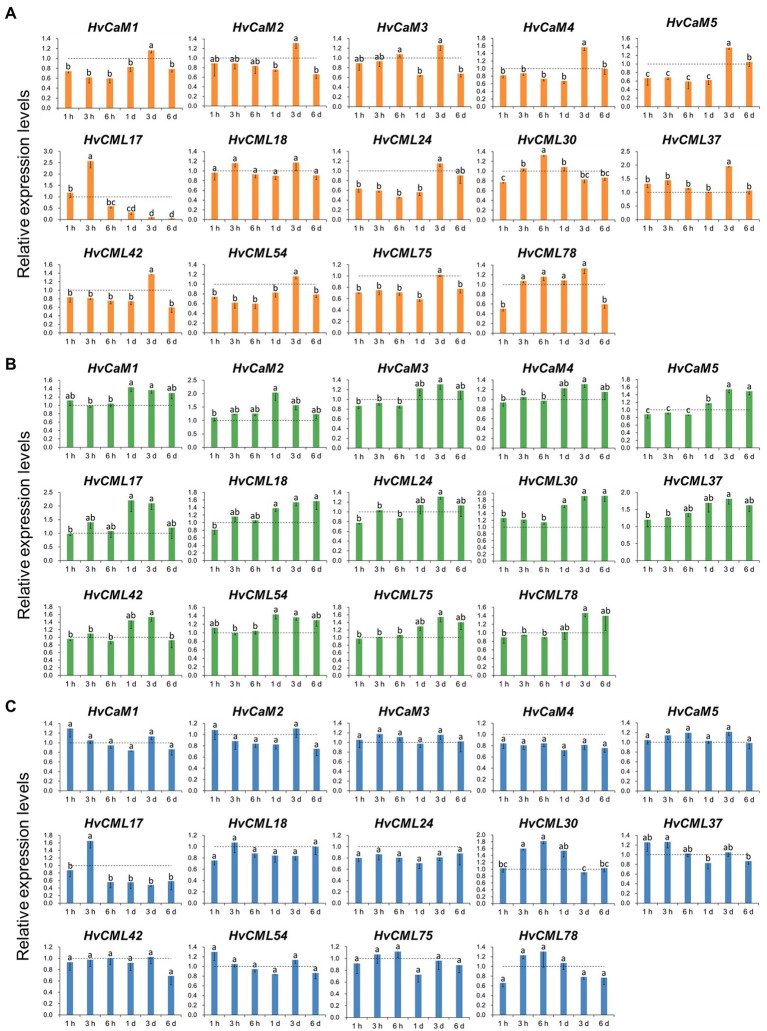
Expression levels of 14 *HvCaMs/CMLs* in response to salt stress **(A)**, potassium deficiency **(B)** and osmotic stress **(C)** at seedling stage. Dotted lines indicated the expression levels of *HvCaMs/CMLs* in control seedlings. Lowercase letters indicated the significant difference at *p* < 0.05.

To investigate the response of *HvCaMs/CMLs* to abiotic stresses, the expression changes of 14 genes (5 *HvCaMs* and 9 *HvCMLs*) were examined through qRT-PCR after salt (200 mM NaCl), potassium deficiency (0.01 mM K^+^) and osmotic (20% PEG8000) treatments (Figure 8). *HvCaM1*, *HvCaM4* and *HvCaM5* displayed similar response patterns to salt stress (Figure 8A). They were down-regulated after salt treatment for 1 h to 1 day and were up-regulated after treatment for 3 days (Figure 8A), indicating the possibility of synergistic response to salt stress. The expression levels of *HvCML37*, *HvCML42* and *HvCML54* were significantly higher after salt treatment for 3 days, while *HvCML17* and *HvCML30* were up-regulated significantly after salt treatment for 3 h and 6 h, respectively (Figure 8A). These results suggested that response patterns to salt stress differed between *HvCMLs* and were time-dependent. Under potassium deficiency conditions, the expression levels of *HvCaMs* were all increased after treatment for 1–6 days and were comparatively higher than those after treatment for 1–6 h. *HvCMLs* displayed similar response patterns to potassium deficiency (Figure 8B). On the other hand, although the expression levels of *HvCaMs/CMLs* fluctuated, no obvious patterns were observed in response to osmotic stress, and no significant difference was observed between different treatments for 11 of 14 examined *HvCaMs/CMLs* (Figure 8C). These results indicated that *HvCaMs/CMLs* might respond to abiotic stress in a synergistic way, and respo.

## Discussion

*CaM* and *CML* gene family has been identified and analyzed in many plant species, such as *Arabidopsis* (McCormack and Braam, 2003), rice (Boonburapong and Buaboocha, 2007), wheat (Liu et al., 2022), *Brassica napus* (He et al., 2020) and papaya (Ding et al., 2018). Compared with relatively well-studied in *Arabidopsis* and rice, *CaMs/CMLs* gene family in barley remains largely unknown. So far, only *HvCaM1* has been functionally characterized, which negatively regulated salt tolerance probably *via* interaction with *HvCAMTA4* to modulate the expression of *HvHKT1;5* and *HvHKT1;1* in barley (Shen et al., 2020). In this work, we identified 5 *HvCaMs* and 80 *HvCMLs* in barley, and then investigated their phylogenetic relationships, sequence characteristics, syntenic relationships, presence/absence variation in pan-genome, expression patterns in different tissues and in response to abiotic stresses.

### Identification of *HvCaMs/CMLs* in barley

A total of 179 genes were identified as *CaM/CML* gene family candidates in barley through preliminary search. As calcium sensor relays, CaMs and CMLs do not have any functional domains and catalytic activities other than EF-hand motifs (Reddy et al., 2011). Based on this criterion, 96 candidate genes were further screened in barley genome. It is noteworthy that CBLs are also sensor relays, and the abovementioned criterion fails to distinguish CBLs and CaMs/CMLs. Thus, the putative *HvCaMs/CMLs*, together with sensor relay genes in *Arabidopsis* (7 *CaMs*, 50 *CMLs* and 10 *CBLs*) and rice (5 *OsCaMs*, 32 *OsCMLs* and 10 *OsCBLs*), were phylogenetically analyzed (Kolukisaoglu et al., 2004; McCormack et al., 2005; Boonburapong and Buaboocha, 2007). The amino acid sequences of these genes were aligned with MAFFT and the phylogenetic tree was constructed with maximum likelihood method. According to phylogenetic evidence, the 96 candidate genes in barley were classified into 5 *HvCaMs*, 80 *HvCMLs* and 11 *HvCBLs* (Supplementary File 8). The 85 *HvCaMs/CMLs* were retrieved for further characterization. Phylogeny-assistant identification was also adopted for *CaMs/CMLs* identification in papaya (Ding et al., 2018).

Totally, 57 *AtCaMs/CMLs* (7 *AtCaMs* and 50 *AtCMLs*), 37 *OsCaMs/CMLs* (5 *OsCaMs* and 32 *OsCMLs*) and 248 *TaCaMs/CMLs* (18 *TaCaMs* and 230 *TaCMLs*) were identified in *Arabidopsis*, rice and wheat, respectively (McCormack and Braam, 2003; McCormack et al., 2005; Boonburapong and Buaboocha, 2007; Liu et al., 2022). In this research, 85 *HvCaMs/CMLs* (5 *HvCaMs* and 80 *HvCMLs*) were identified in barley. *CML* genes were much more than *CaMs* in *Arabidopsis*, rice, wheat and barley, similar results were also observed in other species from lower plants to higher plants (Zhu et al., 2015). Wheat is hexaploid with a genome size of about 17 Gb, which is nearly threefold the genome size of barley (~5.3 Gb). And the number of *CaM/CML* family members in wheat is also nearly three times of that in barley.The genome size of rice (~500 Mb) is 3.7-fold that of *Arabidopsis* (~135 Mb), while fewer *CaMs* and *CMLs* were identified. Notably, *OsCMLs* were absent in cluster IV, V and VI (Figure 1). Besides, orthologous gene pairs between barley and rice involved 49 rice genes, in which 23 genes were not identified as *OsCMLs* (Figure 5; Supplementary File 5). In addition, 53 *OsCMLs* were identified in another research on *CaMs/CMLs* evolution (Zhu et al., 2015). Thus, some members might be left out in previous identification of *OsCMLs*.

### Similarities and differences of *CaMs/CMLs* among barley, *Arabidopsis* and rice

CaMs are highly conserved and ubiquitous in all eukaryotes (Halling et al., 2016). *HvCaMs* were phylogenetically closer to *OsCaMs* than *AtCaMs* (Figures 1, 5). All these *CaM* genes were interrupted by one phase 0 intron and coded polypeptides with 149 amino acids, which possessed 4 EF-hand motifs and contained 9 methionine residues (6.0%, Table 1; McCormack and Braam, 2003; Boonburapong and Buaboocha, 2007). Seven *AtCaMs* in *Arabidopsis* and 5 *OsCaMs* in rice coded 4 isoforms (*AtCaM1/4*, *AtCaM2/3/5*, *AtCaM6* and *AtCaM7*) and 3 isoforms (*OsCaM1-1/1–2/1–3*, *OsCaM2* and *OsCaM3*), respectively. Similarly, 5 *HvCaMs* in barley coded 3 isoforms (*HvCaM2/3/5*, *HvCaM1* and *HvCaM4*; Supplementary File 9). Besides, the amino acid sequence coded by *HvCaM2* was identical to that by *OsCaM1-1* (Supplementary File 9). On the other hand, *CMLs* were more diversified in length, gene structure and methionine percentage. The length of amino acid sequences of *AtCMLs* and *HvCMLs* are similar, ranging from 83 to 354 and 78 to 389, respectively (McCormack and Braam, 2003; McCormack et al., 2005; Boonburapong and Buaboocha, 2007), while the length of *OsCMLs* varies from 146 to 250. Most of *AtCMLs* (31, 62.0%) and *OsCMLs* (20, 62.5%) contained 4 EF-hand motifs. By contrast, EF-hand motifs in *HvCMLs* varied from 1 to 4 (being 20, 18, 23 and 19, respectively; Table 1). Intron-free *CML* genes were predominant in all these three plants (74.0, 75.0 and 66.3% in *Arabidopsis*, rice and barley, respectively). Six *HvCMLs*, together with 6 *AtCMLs* and 6 *OsCMLs*, had higher methionine percentage than *CaMs* (6.0%).

### *HvCaMs/CMLs* in different barley genotypes

The genomic information revealed by reference genome assembly was partly limited to the genotype sequenced and failed to capture the full complement of a species. Pan-genome enabled the characterization of the genetic diversity present in a species. In plants, core genes are often associated with essential metabolic processes, while dispensable genes are related to adaptive functions such as disease resistance and stress responses (Danilevicz et al., 2020; Yocca and Edger, 2022). *HvCaM/CML* was a large gene family, and according to the released first-generation barley pan-genome data (Jayakodi et al., 2020), 81 (95.3%) *HvCaMs/CMLs* were core genes (Figure 6). These results indicated that although the functions of *HvCaMs/CMLs* were largely unknown, they might play diverse and essential roles and were indispensable. The wild barley genotype B1K-04-12 had more *HvCaMs/CMLs* members differed in sequences from Morex than other cultivars and landraces (Figure 6), however, only *HvCML18* was absent in this genotype. Comparatively, there were 3 *HvCMLs* absent in cultivar Golden promise. Thus, there was no necessary connection between evolutionary relationship and presence/absence variation. Recently, it has been found that allelic changes of *cis*-regulatory elements of RAP2.12 are responsible for differentially regulating tolerance to drought and flooding in *Arabidopsis* (Lou et al., 2022). Therefore, in addition to presence/absence variation, variation in *cis*-acting elements should be taken into consideration when characterizing certain *CaM/CML* genes. Notably, *HvCML18* was absent in assembly projection of cultivar Barke, but was present in *de novo* annotation with the evidence from RNA-Seq and PacBio Iso-Seq data. Similar phenomena were also observed for *HvCML18* in landrace HOR_10350. On the other hand, although *HvCaM2* and *HvCML12* in Hockett and *HvCML58* in Golden Promise were identical to those in Morex in nucleotide sequence, they failed to be anchored on certain chromosomes (Supplementary File 6). Discrepancy in chromosomal location of genes was also observed between Morex and other 17 genotypes (Supplementary File 6), indicating more efforts were needed for genome annotation improvement.

### Expression of *HvCaMs/CMLs* in different tissues and in response to abiotic stress

*HvCaMs* were ubiquitously expressed in all examined tissues with high levels, while *HvCMLs* displayed different expression patterns and levels among tissues (Figure 7; Supplementary File 7), which was consistent with tissue expression patterns of *CaMs/CMLs* in *Brassica napus* (He et al., 2020). *HvCaM2* and *HvCML30* were involved in segmental duplication and displayed similar tissue expression patterns and levels (Figures 3B, 6). *HvCML39* and *HvCML12*, *HvCML54* and *HvCML71* were another two segmental duplication gene pairs, however, *HvCML39* and *HvCML54* expressed in all examined tissues, whereas *HvCML12* and *HvCML71* only expressed in certain tissues (Figures 3B, 6). *HvCML34*, *HvCML35* and *HvCML36* were tandem duplicated genes, *HvCML34* and *HvCML35* displayed the same tissue expression patterns and similar expression levels, while *HvCML36* and above two genes differed in tissue expression patterns and levels (Figures 3A, 6). The same was true for the other three pairs of tandem duplicated *CMLs* (*HvCML10* and *HvCML11*, *HvCML71* and *HvCML72*, *HvCML79* and *HvCML80*). These results suggested that gene expression patterns might not interrelate with duplication events. On the other hand, expression of *HvCMLs* were affected by development stages. For example, the expression of *HvCML53* and *HvCML73* were detected in seedling roots but were undetectable in roots at 28 days after pollination (Figure 7). By contrast, *HvCML29* and *HvCML36* did not express in roots from seedlings but expressed in roots at 28 days after pollination (Figure 7). Similar results were observed for expression of *HvCMLs* in developing grains and inflorescences (Figure 7). These results revealed that the expression of *HvCMLs* was more variable than *HvCaMs*, and was tissue-and development stage-dependent.

A series of *cis*-acting elements were identified in the upstream of *HvCaMs/CMLs* coding sequences, including 11 hormone-responsive elements and 7 stress-responsive ones (Figure 4; Supplementary File 4). Thus, the expression of *HvCaMs/CMLs* under abiotic stress treatments was investigated. Salt, osmotic and potassium deficiency stresses all affected the transcription levels of *HvCaMs/CMLs* in a time-dependent way (Figure 8). The expression response of *HvCaMs* to salt stress (up-regulation after 3 days) and *HvCaMs/CMLs* to potassium deficiency (up-regulation after 1–6 days, and relatively higher than that after 1–6 h) displayed similar patterns, indicating these genes might respond in a synergistic manner. Besides, no significant difference was observed in transcription levels of 11 *HvCaMs/CMLs* during osmotic stress (Figure 8), which was also observed in expression of *OsCML1*, *OsCML3* and *OsCML13* under osmotic stress (Chinpongpanich et al., 2012). These results indicated that expression patterns of *HvCaMs/CMLs* were stress-dependent.

## Conclusion and prospects

In the current study, 5 *HvCaMs* genes and 80 *HvCMLs* were identified in barley. Eighty-one *HvCaMs/CMLs* were core genes and only 4 *HvCMLs* were dispensable genes based on the first generation of barley pan-genome. The expression of *HvCaMs/CMLs* varied with plant tissues, abiotic stresses and exposure time of stresses. The obtained results will be helpful for further understanding of CaM/CML family in barley.

## Data availability statement

The datasets presented in this study can be found in online repositories. The names of the repository/repositories and accession number(s) can be found in the article/Supplementary material.

## Author contributions

KC and JW designed the experiments and wrote the paper. KC analyzed the data. LK, WY, SX, and XX performed the experiments. GZ analyzed the data and revised the paper. All authors contributed to the article and approved the submitted version.

## Funding

This work was supported by Zhejiang Science and Technology Major Program on Agricultural New Variety Breeding (2021C02064-3-2), China Agriculture Research System of MOF and MARA (CARS-05-01A-06), the National Natural Science Foundation of China (32101642), and Natural Science Foundation of Zhejiang Province (LQ21C130006).

## Conflict of interest

The authors declare that the research was conducted in the absence of any commercial or financial relationships that could be construed as a potential conflict of interest.

The reviewer GC declared a shared affiliation with the authors KC, WY, XX, and JW at the time of the review.

## Publisher’s note

All claims expressed in this article are solely those of the authors and do not necessarily represent those of their affiliated organizations, or those of the publisher, the editors and the reviewers. Any product that may be evaluated in this article, or claim that may be made by its manufacturer, is not guaranteed or endorsed by the publisher.

## Supplementary material

The Supplementary material for this article can be found online at: https://www.frontiersin.org/articles/10.3389/fpls.2022.964888/full#supplementary-material

## Footnotes

^1^ https://www.arabidopsis.org/

^2^ https://rapdb.dna.affrc.go.jp/

^3^ http://plants.ensembl.org/

^4^ https://www.ebi.ac.uk/interpro/

^5^ https://web.expasy.org/compute_pi/

^6^ http://busca.biocomp.unibo.it/

^7^ https://www.ebi.ac.uk/Tools/msa/mafft/

^8^ http://cbi.hzau.edu.cn/bnapus/index.php

^9^ http://plants.ensembl.org/info/data/ftp/index.html

^10^ https://meme-suite.org/meme/tools/meme

^11^ http://bioinformatics.psb.ugent.be/webtools/plantcare/html/

^12^ https://apex.ipk-gatersleben.de/apex/f?p=284:49:::::P49_GENE_CHOICE:4
