# Supplementary material for: Calmodulin and calmodulin-like gene family in barley: Identification, characterization and expression analyses
Source: Front Plant Sci. 2022 Aug 19;13:964888. doi: 10.3389/fpls.2022.964888 (PMC9439640; doi:10.3389/fpls.2022.964888)
Supplement: Supplementary file 2 [file Table_2.docx]

**Nucleotide and amino acid sequences of *HvCaMs/CMLs***

Nucleotide sequences

>HvCaM1

ATGGCGGACCAGCTCACCGACGAGCAGATCGCCGAGTTCAAGGAGGCCTTCAGCCTCTTCGACAAGGACGGCGACGGTTGCATCACCACCAAGGAGCTCGGAACCGTGATGCGATCCCTTGGGCAGAACCCCACGGAGGCGGAGCTGCAGGACATGATCAACGAGGTTGATGCGGACGGCAACGGCACCATCGACTTCCCAGAGTTCCTGAACCTGATGGCGAGGAAGATGAAGGACACGGACTCGGAGGAGGAGCTCAAGGAGGCCTTCCGCGTGTTCGACAAGGACCAGAACGGCTTCATCTCGGCTGCAGAGCTGCGCCATGTGATGACCAACCTCGGGGAGAAGCTGACGGACGAGGAGGTGGACGAGATGATCCGGGAGGCGGACGTGGACGGCGACGGCCAGATCAACTACGAGGAGTTCGTCAAGGTCATGATGGCCAAGTGA

>HvCaM2

ATGGCGGATCAGCTCACCGACGACCAGATCGCCGAGTTCAAGGAGGCCTTCAGCCTCTTCGACAAGGACGGAGATGGTTGCATCACCACCAAGGAGCTGGGAACAGTCATGCGCTCGCTGGGGCAGAACCCAACGGAGGCTGAGCTCCAGGACATGATCAATGAAGTCGACGCTGATGGCAACGGCACCATTGACTTCCCAGAGTTCCTCAACCTGATGGCCCGCAAGATGAAGGACACTGACTCAGAGGAGGAGCTCAAGGAGGCCTTCAGGGTGTTCGACAAGGACCAAAACGGCTTCATCTCTGCTGCTGAGCTCCGCCACGTCATGACGAATCTCGGCGAGAAGCTCACCGACGAGGAGGTGGACGAGATGATCCGTGAAGCTGACGTCGACGGTGATGGCCAGATCAACTACGAGGAGTTCGTCAAGGTCATGATGGCCAAATGA

>HvCaM3

ATGGCCGACCAGCTCACCGACGACCAGATCGCCGAGTTCAAGGAGGCCTTCAGCCTCTTCGACAAGGACGGAGACGGCTGCATCACCACCAAGGAACTTGGAACTGTGATGCGTTCGCTAGGGCAGAACCCCACTGAGGCAGAGCTTCAGGATATGATCAATGAAGTGGATGCTGATGGCAATGGAACGATTGACTTTCCTGAGTTCCTCAACCTAATGGCACGTAAGATGAAGGACACTGATTCTGAGGAGGAGCTTAAGGAGGCCTTCCGCGTGTTTGACAAGGACCAGAATGGTTTCATCTCGGCGGCTGAACTCCGCCATGTCATGACCAACCTTGGTGAGAAGCTGACAGACGAGGAGGTGGATGAGATGATCCGCGAGGCTGATGTCGATGGTGACGGACAGATCAATTACGAAGAGTTTGTGAAGGTGATGATGGCCAAGTGA

>HvCaM4

ATGGCGGACCAGCTCACCGACGACCAGATCGCCGAGTTCAAGGAGGCTTTCAGCCTCTTCGACAAGGATGGGGACGGTTGCATTACGACCAAGGAGCTGGGAACTGTCATGCGTTCCCTGGGGCAGAATCCCACTGAGGCAGAGCTTCAAGACATGATCAATGAGGTGGATGCCGACGGCAATGGAACAATTGATTTCCCCGAATTCCTCAACCTTATGGCCCGCAAGATGAAGGACACTGATTCTGAGGAAGAGCTCAAGGAGGCATTCCGTGTGTTTGACAAGGATCAAAATGGTTTTATCTCTGCTGCTGAACTGCGCCATGTCATGACCAACCTTGGTGAGAAGTTGACTGATGAGGAGGTTGACGAGATGGTCCGTGAGGCTGATGTTGATGGTGACGGCCAGATCAACTATGACGAATTTGTTAAAGTCATGATGGCCAAGTAA

>HvCaM5

ATGGCGGACCAACTCACCGACGACCAGATCGCCGAGTTCAAGGAAGCCTTCAGCCTATTCGACAAGGACGGCGACGGTTGCATCACAACTAAGGAGCTGGGAACAGTCATGCGTTCGCTGGGGCAGAACCCAACGGAGGCAGAGCTCCAGGACATGATCAACGAGGTGGATGCAGACGGCAACGGCACTATCGACTTCCCGGAATTCCTCAACCTGATGGCTCGCAAGATGAAGGACACCGACTCGGAGGAAGAGCTCAAGGAGGCTTTCCGGGTGTTCGATAAGGACCAGAACGGCTTCATCTCGGCCGCGGAGCTCCGCCACGTCATGACCAACCTCGGCGAGAAGCTAACGGACGAGGAGGTGGACGAGATGATCCGCGAGGCCGATGTCGACGGCGACGGGCAGATCAACTACGAGGAGTTCGTTAAGGTGATGATGGCCAAGTAA

>HvCML1

ATGGCAGGCTCCTGCCGCAGCGTCCACTTCTATTTTTGGGCATCAGATTCTACCTCATCCGGCGCCCCCCTCCCTCCCAGGTCGACGGAGATTGCCGTAACCAAGACGGATTCCATCCCCCACTCAGAATCCAATCCAACAACAACCTACACCACCTACAACAAAACCCTCAATTCCACCACGCAAGGCAGCACCCAGGCATCAAGGGCTGTTTCTGAAGAAGAAGAAGAAGAAGAAGAAGGAGGAGGAGGAATGGGGGGCGCGGCGTCGAGGCTCGCTGCCCCGATAAAGCACCGGCGGGGGGAGAAGGAGCTGGACAACAGGGTGGCGGAGGCGCTGCGGGAGCGGGCCAAGGCGAGGACGAGGACGTTCCGGTCGGTGAACAGCATCACCATGCGCCTCCCTCGCTTCAAGGACGGCCTCAAGGACATCCGGGACGTCTTCGACCACTACGACGCCGACTCCAACGGGACCATCGACAACGAGGAGCTGCGGAGCTGCCTGAGCAAGCTCGAGGTGCGCATGTCGGAGAGGGAGTCGGACGACGTGCACCGCTACTGCGACGTCGACAGCCGCAAGGGGATCCAGTTCCAGGAGTTCGTCGTCCTCATCTGCCTCATGTACCTGCTCTTCGGCCCCAACGTCACGCGCCGGGTTTCCGAGTTCGAGTCGGCCAAGCTCAACTACATCTTCGACGAGCTCATCGACGCCTTCCTCTTCTTCAACAGGGACGGCGACGGCAAGGTCACCAGGAAAGACGTCACCCAGAGGATGAACGAGGAGTGTGACCGGGAGAGGACGCCCGCCCACATAACCACGCAGCTATTCAAGGAGATGGACCTGAACAAGAACGGCAAGGTGAACCTCAAGGAGTTCCTCTTCTCCATGATCAGATGGGCGGGGCTCGAGCCCGAAGAAGATGATGAAAGCAATGACATTTCCCCCTAG

>HvCML2

ATGGCGAACGGCGTGGGCTCGGCGAAACCGGAGATCTGCGCCGGGTTGGGCATGCCGATGGCGGAGCTGGAGCAGGTGTTCCGGCGGTACGACGCCAACGGCGACGGCAAGATCTCGGCGGACGAGATGGCGTCCGTGCTGTGCGCGCTGGGCGCGCCCCCGGGGCCAGGGGAGGTGCAGAGCATGATGGAGGAAATGGACGCCGACAGGGACGGCTTCGTCGACCTCCACGAGTTCGCCGCCTTCCACTGTGGCCCCTGCAAGGCCGGCGCCGGCGCGGACGCCAAGGAGCAGGAGGACGCCACGGAGGCGGAGCTCAAGGAGGCCTTCCGGATGTACGACGCCGACCGCAACGGGCTCATCTCCGCGCGGGAGCTTCACCGCGTGCTCCGCCAGCTCGGGGACAAGTGCTCTGTTTCTGACTGCTCGCGGATGATCCGATCCGTCGACGCCGACGGAGACGGCAGCGTCAACTTTGAGGAATTCAAGAAGATGATGGGCGGCGGAGGATCAAATCATTCCCGACTCTCCTTCAAGTCATTCGACGTGCTCGGCCTAGATGTCCTCCTTCCAACCCCGATGACAGGACATGTATTGCTGCAGCCGCATAGTTTTGGTTCTAACTTTTGCATACGGTCTCGGATTAAGACGATTCAACTATCAAAAATTACCGCTTCGATGAGATGA

>HvCML3

ATGAGCGAGGCACCCGGGACGACCGCCGCCGCCGGAGACGATCCTGACGCCACCGCCAACGCCAATCAGCAGCAGCAGCAGCAGCAGCAGCAGGCGCAGCCCGAGGCGCAGGACGACGCGGACCAGCTGTCGGAGCTGAGGCAGATCTTCCGGTCCTTCGACCGCAACAAGGACGGCAGCCTGACGCAGCTGGAGCTGGGCTCCCTCCTCCGCTCCCTCGGCCTCAAGCCCAGCACCGACGAGCTCGACGCCCTCATCCACCGCGCCGACACCAACTCCAACGGCCTCGTCGAGTTCTCCGAGTTCGTCGCCCTCGTCGCGCCCTCGCTCCTGGACGACCGCTCCCCCTACTCCGAGGACCAGCTCCGCAGGCTCTTCGAGATCTTCGACCGCGACGGCAACGGCTTCATCACCGCCGCCGAGCTCGCGCACTCCATGGCCAAGCTCGGCCACGCGCTGACCGCCAAGGAGCTCACCGGGATGATCGAGGAGGCCGACACCGACGGCGACGGCCGGATCGACTTCCGTGAGTTCTCCCGCGCCATCACCGCCGCCGCCTTCGACAACGTCTTCTCCTGA

>HvCML4

ATGGGCAAGATGCGGTCACTCTTCTCACGGAGCCGGAGCGGCAACGGTGGGAGCCGGCGGTCCACCTCCTCCTCCAGGTCGTCAGCGCCGCCGTCGCCCGCGCGCGGGGCCTCGAGGGAGGACGAGATGGAGCGCGTGTTCCGCAAGTTCGACGCCAACGGCGACGGCAGGATCTCGCGGGCGGAGCTGGCGGCGCTCTTCGAGAGCGTGGGCCACGCGGTCACCGACGACGAGGTGGCGCGCATGATGGAGGAGGCCGACGCAGACGGCGACGGCTACATCAGCCTCGCCGAGTTCGCCGCCATCAACGCCGCGCCCGACGCAGCCGTCGAGGAGGACCTCCGCCACGCCTTCCGCGTCTTCGACGCCGACGGCAACGGCGTCATCTCCCCCGCCGAGCTCGCGCGCGTGCTGCGGGGGCTCGGCGAGGCCGCCACCGTCGCGCAGTGCCGCCGCATGATCGAGGGCGTCGACCGCAACGGCGACGGCCTCGTCTCCTTCGACGAGTTCAAGCTCATGATGGCCAACGGCGCCGGGTTCGCCATCGCCCAGGGCAACGTCCGCGCCTGA

>HvCML5

ATGGCGGCCAAGCTGACCCGGGAGCAGGCGGACGAGTGCAAGGAGGTGTTCGACCTGTTCGACGGCGACGAGGACGGCCGCATCGCCGCGGGCGAGCTGGTCACGGCGCTCCGCTCGCTGGGCCAGAACGTGGACGAGGCCGAGGCGCGGGGCTTCCTCGAGGACGCGGGCGCGGGCGCGGGCGCCGCCGCCGTCGACCTCCCCACGTTCCTGGCCGTGGCGGCGCGCAAGGCCAACGCCGGCGTGTCGGCCAAGCGCCTGGTCGAGTGCCTCGACGCCTTCGACGACGACGGGAGCGGCGTCATCCCCGCGGAGCAGCTGCGGCAGGTGATGCTGACCCACGGCGACCGGCTGACGGAGGAGGAGGCCGACGAGCTTGTCCGCAAGGCCGACCCCCGCGGCGAGGGCCGCGTCCAGTGCAAGGAGCTCGTCAAGGTGCTCATGAACAACAAGTGA

>HvCML6

ATGGTGCACACCGCGACGGCCGAGTGCTTCAGCAGCGTGTTCGCCTCATTCGAACGCGACGCCGACGGCAGGATCTCGGCGGCGGAGCTGTGGCTGTGCATGAAGGCGGCGCTGGGCGAGGACGTGTCAGCGCAGGACGCCGAGGCGCTCGTGGCGTCGGCCGACGCCGACGGAGACCAGCTTCTGGACGAGCAGGAGTTCCTCCGGCTGGTGGCGCGGCCGGAGACGGAGGAGGAGGAGTGGTGCAGGGGGCTGAGGGAGGCATTCGCGATGTACGAGGTGAAGGGCGAAGGGTGCATCACGCGGTCCAGCCTGATGCGGATGCTCGCCAGGCTGGGGTCCGAGCAGGGCATCGAGGAGTGCCGCGCCATGATCCGCAGGTTTGATTTGAATGAAGACAGAGTGGTTTGCTTCGACGAGTTCAAGGTTATGATGGATGCGTAG

>HvCML7

ATGTCGACCCATCCTACAAATCTTGCCAGCTACAGATCGCCCCGATCAACCAGGACGACCGTCGCCGCCAGAGACGATCCTGACGCCACCGCCAACGCCAATCAGCAGCAGCAGGCGCAGCCCGAGGCGCAAGACGACGTGGACCAGCTGTCGGAGCTGCGGCAAATCTTCCGGTCCTTCGACCGCAACAAGGACGGCAGCCTGACGCAGCTGGAGCTAGGCTCCCTCCTCCGCTCCCTCGGCCTCAAGCCCTGCACCGACGAGCTCGACGCCCTCATCCACCGCGCCGACACCAACTCCGAGTTCGTCGCCCTCGTCGCGCCCTCGCTCCTCGACGACCGCTCCCCCTACTCCGAGGATCAGCTCTGCAGGCTCTTCGAGATCTTCGACCGCGACGGCACTGGCTTCATCATCGCCGCCGAGCTCGCGCACTCCATGGCCAAGCTCGGCCACGCACTCACCGCCAAGGAGCTCACTGGGATGATCGAGGAGGCCGACACCGACGGCGACGGCCGGATCGACTTCCGTGAGTTCTCCCGCGCCATCACCGCCGCCGCCTTTGACAACGTCTTCTCCTGA

>HvCML8

ATGGACAAGTCCCCAGCCACTGCGGGATGCCATCTTGAACCACTTTTCCTAGAGCCACTTGGTATTCTCATCCTTTTCATCCTAACCTGGTTCATCTCAGAGGTTCAAAGACTCCTTCCCAGTTCTTGCCAATCCTGCAGTTGTCCTGTCTCCACGACAACCTCGCCGCCGGTCCTCGCGGAGACATCAAAGGCCCCAAATAAGAGTGAATATGTGGAGATGAATGCGAAGCAGTCAGATGCAGAGATAGCCATGAGAAAGATGGGATTCGATTTTGATCAAGAGAAAAGCTGTGAACATATTTCTACGCTTTTCGACGACGACGAACCAAGCTTCCAAGAAGTGAAAATGGCATTCTTGGTTTTTGATGAGAACAATGATGGGTACATCGATGCATTGGATTTGCGACGAGTCCTCCACAACTTAGGATTAGGTGACCGGGTAGGGGTCAGTGAGTCTGAGCAGATGATTGCTAGATATGACATGAACAATGACAGGAGGATAGATCTGATGGAGTTCACTAAGGTTTTGGAGGATAGCTTTTGCTAA

>HvCML9

ATGCACCCACCGACCCGGTCACCGCCAACGCCGACGACGCGTCTTCCTCCCTGCCGCCAGTGCCCGGTAATCTCTCCTCTCCTCTCCTCCATAAATATATACCAAAGTCGTCGTCTCCACTCTCCACCCCGGCCCGGTCATCATCCCAAACCCATTCTCCATCCCCCACCTCCACCTCCTCCGATCCCATCCCATCGGCCTTCCACGATGACGAAGGCGTCGCCGGCGCTGCGGGGCAGCCAGCTGAAGCAGCTGCGGTCCCTCTTCGACCGCTTCGACATGGACGGCGACGGCAGCCTGACCCAGCTGGAGCTGGCCGCGCTGCTCCGGTCCCTGGGCCTGCGCCCCACGGGCGACGAGTCGCGCGCGCTCCTCCTCGCCATCGACGCCGACGGCAGCGGCACCGTGGAGTTCGACGAGCTGGCGCGCGCCATCGCGCCGGTCCTCACCGCCCACGCGCCGCGGCTCGTCGACCAGGCGCAGCTGCTCGAGGTCTTCCAAGCCTTCGACCGCGACGGCAACGGCTACATCTCCGCCGCCGAGCTCGCGCGCTCCATGGCCAAGCTCGGCCAGCCGCTCACGTTCGACGAGCTGCGGACCATGATGCGGGACGCCGACGCGGATGGTGACGGCGTGATTAGCTTCGGCGAGTTCGCCGCCGTCATGGCCAGGTCCGCGCTCGACTTCCTCGGCGTCCCCGCCGCCTGA

>HvCML10

ATGTGGGTGAAGATGTTGATGGATCAAAACATGCTCATCGCATTGGTGTCATCTCTCCTGATGTTGATCTTGGGGCCATTGATCAAAGATATCATACTGGTAAGCAAAAAGATCTGGAGCTTCTTATGCACACTTACAAAGTACCTTGTGCACAATGACACCCTCGCCGTAGACTCTGTGGTGCTTGATGATAACATTTCACCGCCGGCACAACTAGCTTGTGGTGGAGGATTGACCTCTGGTGACATAGAAATTGTCACGGCGAGGCTAGGTCTTACCAGGTTTAGTTACCAAGGGTGTGAAGGACTTGGTGTCGTAGAGGAGTTGATGGACGGTAAGCAAGCGAGCCAGGACGAGCTAGAGGAGGCCTTCTGCATTTTCGACCGTGATGAGGACGGGTTCATATGCACCGGGGAGTTGTGGAACGTGATGAGGAGGCTTGGGTGGAAAGAAGGGGCGATGTATGAGGACTGTGTGAGGATGATCCGTGCCTTCGATGAGGATGGAGACGGAAAGATCAGCTTCCTCGAGTTCAGAAGGATGATGGAGAATGCCGTTTAA

>HvCML11

ATGTGGGCTGTGGTGATTATGGGTCAAAACATCCTAATCGCATTGGTGTCATCTCTCCTGACGTTGATCTTGGGGCCATTGATCATAGATGTCATCCTGGTAAGCAAAAAGATATGGAGCTTCCTGCACACATTTACAAAATACTTAGTGCATGATGACACCCTCGTCATCGACTCCGTGGTGCTTGATGATAGCCCCATGTTGCCGGCGCAACTAGTTGGCGGCGGCGGATTGACCTCTGTTGACATAGAAATTGTCACGACGAGGCTAGGTCTGGGCGGGTGGAGTTACCAAGGGTGTGAAGGAATCGGTGTTGTAGATGAGTTGATTGACGGTAAGCAAGCGAGCGAGGACGAGCTAGAGGCGACCTTCTACATTTTTGACCGTAACGAGGACGGGTTTATATGCGCCGGGGAATTGTGGAATGTGATGAGGAGGCTGGGGTGGAAAGAAGGGGCGATGTATGAAGACTGTGTGAGGATGATCCGCGCCTTCGATGAGGATGGAGATGGGAAGATCAACTTCCTAGAGTTTAGAAGGATGATGGAGAATGTTGTTTAA

>HvCML12

ATGGCCGGCGAGCACCAGCAGAGCCAGGGCGCGGCGAAGCCCCTGTCCAAGGGCGCGCCGTCGCCGTCCTTCCGCCTGCGCAACGGCAGCCTCAACGCGGTGCGGCTGCGGCGCGTGTTCGACATGTTCGACCGCAACGGCGACGGCGAGATCACGGTGGACGAGCTGGCGCAGGCGCTGGACGCGCTCGGGCTGGAGGCGGACCGCGCCAGCCTGGCCGCCACCGTCGGCGCGCACGTGCCCCCCGGCGCCTCCGGGCTCCGCTTCGAGGACTTCGAGGGCCTCCACCGCGCGCTCGGCGACGCGCTCTTCGGCGCCCTCGCGGACGACGGCGAGGACGGCGGCGCCGGCGGGGAGGACGAGGAGGAGATGCGGGAGGCCTTCAAGGTGTTCGACGTCGACGGCGACGGCTTCATCTCGGCCTCCGAGCTGCAGGAGGTGCTCAAGAAGCTCGGCATGCCCGAGGCCAGCAGCCTGGCCAACGTCCGGGAGATGATCTGCAACGTCGACCGCGACAGCGACGGCCGCGTCGACTTCGGCGAGTTCAAGATCATGATGCAAGGGATCAACGTCTGA

>HvCML13

ATGGCAGACGCGATTGCGCCGCGACCGGTCCTCCCCCGCGCGCTCTCGTTCCGAGAGCCGCTGCTGCTCATCCCCTACTTCCTCGGCTTCCTCGGCACCGTCGCCTCCGCCTTATTCTACAACTATGCTTCCTTCCTTCGGTCCTTCGCCAGGTCGATCGTCGTCCCCTCGCCGGCCGCGGCTTGCGCCAAGTGCACGTACGCAACATCTTCAGTACCGTGCTGCGAGGACGCCGACGCCGACGCCGAGGAGATGAGGAAGGAGGAGGTGGAGGCCATCATGGCTAGAATCGGGCTGGGAGTGACCGGTGCCGGCGAGGGGCTGAGGGCCAGCATGGGCCACAACGAGGTGTCCCGGCTGTTCGACGCCGAGGAGCCGAGCTTCGCGGAGGTGCGGCGCGCGTTCGCCGTGTTCGACGGCGATGCGGACGGGTTCATCGGCGCCGCGGACCTGCAGGGCGCCCTGACCAGGCTCGGGCTCCCGGATGTCGACTCCGCCGCGTGCGAGGCGACGATCTCGTCGAGCTGCGGGTCCACGGACGGCAGGATGAACCTGTTCCAGTTCGTCAGGTTCCTCGAGGACGGCCTCCAAGAGTCTGGGTGGATGGATTGTGCAACAAATTAA

>HvCML14

ATGGCAGACGCGACGGCGCCGCGACCGCTCCTCCGCCGCATGCTCTCGTTCCGGGAGCCGCTGCTGCTCATCCCCCACCTCGTCTTCTTCCTCGGCACCGTCGCGTCCGCCTTCTTCCACAGCTACGCCTCCTTCCTGCAGTCCTTCGCCAGGTCCGTCGTCGTCCCGTCGCCGGCCGCCTGCGCCAAGTGCGCGTACGCAGCGTCGTCGTCGGCGGTCTGCTGCGATGACGCCGCCGTCGCCGAGGAGCGGGGGGAGGGCGAGGAGCTGAGGAAGGAGGAGGTGGAGGCGATCATGGCCAGGATCGGGCTGGGCGTGGCCGGCGCCGGCGAGGGGCTGAGGGCCAGCATGGGCCGCAACGAGGTGTCCCGGCTGTTCGACGCCGAGGAGCCGAGCTTCGCGGAGGTGCGGCGCGCGTTCGCCGTGTTCGACGGCGACGCGGACGGGTTCATCGGCGCCGCGGACCTGCAGGGCGCGCTGGCCAGGCTCGGGTTCCCGGAGGTCGACGCCGCCGCGTGCAGGGCCATGATCTCGTCCAGCTGCGGGTCCACGGACGGCAGGATGAACCTGTTCCAGTTCGTCAGGTTCCTCGAGGACGGCCTCTGCTGA

>HvCML15

ATGGCAGGGCGGAGGTGGCTCCTCTGGCCGGGAGGGCTCACGGTGGAGGAATTCAAGGAGTGGCTGAAGCAGTTCGACGTGGACGGCGACGGCCGGATCAGCAGGGCCGAGCTCCGCAAGGCCATCCGCAGCCGGGGGGTCTGGTTCGCCACCCTCAGGGCCGGCCGAGCCATCCGTCACGCCGACAGGGACAAGAGCGGCTACGTCGAAGACGCCGAGATTGAGAATCTCGTCGCATTCGCGCAGAAAGACCTCGGCATGAAGATCTCTGCCTGGTAG

>HvCML16

ATGCCGATAAGGGGCGTCCCGTGTGCGTGGACGGTGGAAGAATTCAAGAGCTGGCTGAAGCAGTTCGACGTGGACCGCGACGGCAAGATCAGCAAGGCCGAGCTCCGCCAGGCCATCCGCCGCCGGGGCTGCTGGTTCGCCACCGCTAGGGCCGGCCGCGCCGTCCGCCGCGCCGACAGAGACCACAACGGCTACGTCGACGACGCCGAGCTCGAGAACCTCGTCGCTTTCGCACGGGAACACCTCGGCATGAATATATCTGCCCGTTAG

>HvCML17

ATGGCGATCAGGGGGATACCTTCGGCGCGGGAGATGACGATGGAGGAGTTCAAGGAGTGGCTGAAGCAGTTCGACGTGGACGGCGACGGCCGGATCAGCAGGGCCGAGCTCCGCGAGGCCATCCGCCGCCGCGGGGGGTGGTTCACCACCCTCAGGGCCGGGCGCGCCGTCCGCCGCGCCGACAGGGACAACAGCGGCTTCATCGACGACGCCGAGGTTGAGAACCTCGTCGCCTTCGCGAAGAAGGATCTCGGCATGAGGATCTCTGCCTGGTAG

>HvCML18

ATGTCTACAATGAAGGGACAGACAAGGAGGGAGAGGCCTAGAACTCGCCCTCATGGCCTCACACAACAGAAGAGGCAGGAAATAAAGGAAGCATTTGATCTTTTCGACACCGATAACTCTGGAACCATCGATGCCAAAGAGTTGAATGTTGCGATGAGAGCCTTGGGATTTGAGATGACAGAAGAGCAAATCAATCAGATGATTGCTGATGTAGACAAAGATGGCAGTGGATCGATAGATTATGAGGAGTTTGAGCACATGATGACTGCCAAGATTGGGGAGAGGGATACTAAAGAAGAGCTTACAAAGGCGTTCCGCATTATTGACCAAGATAAAAATGGGAAGATTTCAAATGTTGATATTCAGCGTATTGCCAAGGAGTTGGGTGAGAACTTCACTCTCCAAGAGATCCAAGAGATGGTTCAAGAGGCAGATCAAAATGGCGATGGCGAGATAGATTTTGGCGAGTTTGCCAGGATGATGAAGAAGACCAGCTATGGTGGTTACTAG

>HvCML19

ATGCTTGAGCAGATTGACAGTAACACAGATGGATTTGTTGATTTCGAAGAGTTTGTTGCTGCCACATTACATATGCATCAGCTGGTGGAACATGATGCTGAGAAGTGGAAATCATTGTCTCAGGCTGCATTTGATAAATTTGATGTTGACGGTGATGGCTACATCACATCTAATGAACTGAGAATGAATACAGGACTGAAGGGTTCTATTGACCCCCTCCTGGAGGAGGCTGATATTGACAAAGATGGCAAAATAAGCCTTGATGAATTTCGTAAGCTCTTGAAAACTGCAAGCATGAGGTCATGCAACCCAACTCCACGGAGTGTTTCCAAGTAA

>HvCML20

ATGGTCCAGGGCAAAGGCTTTGCGCCACCACCACCAAACCTCTCCCCACCCAACCAACTCAACTCGAGCGATCTTCAAAGCCTCCACAGCAAAGCAACACCACCACCCTCCATGCCACCCTCCGGCCTCCTCTCCTACATCCCCACCAGCCTCTCCTCCATCCTCCCGGCCCGCGGCTGCGGCGCGGCCCCCTCGCCGTCGCCCCCACCGCCGGCGTCTCCGGCCCCGAGCAAGATGTCGCCCTCGGTGAAGGCGGCGGACCGCGCGGAGCTGGCGCGCGTGTTCGAGCTGTTTGACCGGAACGGCGACGGGCGCATCACGCGGGAGGAGCTGGAGGACTCGCTGGGCAAGCTGGGCATCCCCGTGCCGGGCGACGAGCTGGCCGCCATGATCGCGCGCATCGACGCCGACGGCGACGGGTGCGTGGACGTGGAGGAGTTCGGGGAGCTCTACCGCACCATCATGTCCACGGGCAGCGGCGGGGGCCAGAAGGGCTCGTCGGACGCGGAGGCGGAGGAGGAGGACGAGGACGAGGACATGCGGGAGGCTTTCCGGGTGTTCGACGCCAACGGCGACGGGTTCATCACGGTGGACGAGCTGAGCGCCGTGCTGGCGTCGCTGGGGCTGAAGCAGGGCCGGTCGGCCGAGGAGTGCCGCCGCATGATCGGCCAGGTCGACCGCGACGGCGACGGCCGCGTCGACTTCCACGAGTTCCGCCAGATGATGCGCGGCGGCGGGCTCGCCGCGCTAGCCTGA

>HvCML21

ATGGCGGGAGAGGAGCAGCCCCTGACGGAGTACGAGAAGGAGCGGCTGGCGCGGATACGGGAGAACGAGGCCCGCCTCGAGGCCCTCGGCATCCGCCGCCTAGCGGCGTCCCCCCTCCTCAACCAGCCGTCCTCCGCGGCGGCGGCGGCGGCGGCGGGGGCCAAGAGAAAACAGAAGAAGCGATCCGACGATGCGGACGAGGAGTACCTCCCGTCCGACGGGGGCGGCGGGGAGGAGAGCTCGTCGGCCAGTGACCAGGACACCGAGGAGGACTTCAAGCCTTCCTCTAGGTCTAACCAAAAGGGAAAGGCAAAGAAGAAACTGAACTTAGGAAGCCCTTCTAAAAGCACCTTTAGAGAAGAAGATGCTCCCTTAACCGATTTTATGGATGACGATGCAGCCTTACAACAGGCGATTGCACTCTCCCTGGCAGAACCTTCAAAAAGCTCCGTGACAACAGCAGAACCTTCAAAAAGTTCCGTGACGACAACAACAGCTGCAGAACCTTCAAAAAGTTCCGTGACGACAACAACAGCTGCAGAACCTTCAAAAAGCTCCGTGACAACAGCAGAACCTTCAAAAAGTTCAGTGACGACAACAACAACTGCAGAAACTTCAAGCAGAGGAGCCAAAGGACGGAAAGGCACGCCGTGCAAAAATGACAATACCACACCTGTTAAGGACTCTGCTAAAAATAGGAAGACAAAGAAGCAGGTCAGGAGCAGAATTCAACTGTCGGAAGATGATGTGGTGGCAATATTCTTCTCATTTGATGAAGCTGGAAAAGGATACATCGCACCCTGGGATCTTGAAAAAATGGCCAACGTAAATGATTTCATCTGGACAGATTTTGAGCTATCTAATATGATCAATTTCTTTGATAATGACAAAGATGGAAAGATAAGCCTCGAGGAATTCCGAGCCATCGTGTCCCGATGCAACATGCTGCAAGAGCCAGGGGAGTGA

>HvCML22ATGGCATGCCAGAGTGGGATCCATCAGAGCGCTGCTGGTAGCTCAAGCAGATTGCTGTGTAGCCATCCTGCTGATTCGTTGCACCTGAACAACAAGAACAGTTCAACGCCGCCCACCAAGACGCTCCTGCAAGGCTGCAACGCGGATGTGGACAACAATGGGACCATTGACTATATAGAGTTCATCGCTGCAACATTGCATCTGAATAAACTGGAGCGTGAGGAGCATCTGGTGGCAGCCTTTTCATATTTTGACAAAGATGGAAGTGGTTACATCACAGTGGATGAGCTGCGGCAAGCTTGCCTGGAGCATAACATGCCAGATGCTTTTCTTGATGATGTCATTAAAGAAGCTGACCAGGACAACGATAAGTGCGGTCTTGATTCTATATATTTATTATTCTCGAGATATGGTATATCTTGGTTACCTATTAATCCATTACTGTCTTCCTAA

>HvCML23

ATGCAGAGCGGCCAGCAGCAAGCGAGGAGGAAGAACGCGCAGGTGCTGGACGGGTCGGACATCCGGGAGCTGGTGGAGAACAAGGAGGCGTTCGCGAAGTTCGTGGAGAACAAGTTCCGGCACCTTGACGCCGACGGCGACGGGAGGTTGTCGTTGAAGGAGCTCCAGCCCGCCGTCGCGGACATCGGCGCCGCCATCGGGCTGCCGGCGAGGGGGTCGTCGGCGCAGGCGGACCACATCTACTCCGAGGTTCTAAATGAGTTCACCCACGGGAAGCAAGATTCGGTGAGCAAGCCGGAGTTCCAGCACGTGCTGTCCGACATACTCCTCGGCATGGCGGCGGGGCTCAAGAGGGACCCAATCATGATCCTCAGGGTGGACGGGGAAGACCTGAACGAGTTTGTTGAGAGCGCTGCGTACGAAGCAGAGGCAGTCGCCATATTCTCTCAAATCGAGTCGGGAAATTCGTCGTTGCGACAATGTTTGCCCGCCGCTCTTCGACAGCTGACGGTCGATCACGGCATGCCACCGGCTTCGGACTCTCTGGTTATGGAGAAGATCATAGTACCTGCATTGCAGGAACTGCCAGCGGACCAGCTTGACCAGCCAGCATCGCAAGAGGTCTTCTTCCAAGAGTTCAAGAAGTATTTGGGAATGATCGCCCGGCGACTCCAGGAGTGCCCGATAATTGTGGCGCACACGGAGAACACCTTTGATGGGGCTGGCATCAGGAAGATACTGTCGAACAAGTTCGAGTTCGACAAGCTGCTGGATTCTGTCTGGGGAGATGTCCCCAAGGAACACAAAGATAGAACATCAAAGAAGTACCTTCGCGTCGCGTTTGACAAGATGGCCGCATCAGTAAATCTACCACCGTATGGAGCTGTTAATCAGGTGGATGCTATGGTGAACGACGCGTTCAAGATGGCGAACGCCGACGACGGGAAGGCGGTGGACGAGGCCGAGTTCAAGAAGCTGCTGACCGAGATCCTAGGAGCGGTGATGCTTCAGCTAGACGGCAACGCGATCGCGGTCTCCACCAACACCGTCCTCCACGAGCCCATGTCCACTTCGTCGACCTTGTTGTCACCGTCGCCGTCGTCGCCCACGGTGTCATCGCCGAGCGAGTAG

>HvCML24

ATGGGGCAGGTCTGGGCGTCTCTGCAAGAGAAGCTCCAAGGGCGGCACTGGAAGGAGAGGCAGGTGCGGAAGATCACCGACAAGGTGTTCGACCGGCTCACAGAGGACACGCAGAGGCGCGAGAAGGAGGCCCTCCAGTTCGAGGAGGTCTACATCGCCGTCCTCTGCGTCTACAACGACATCAACAAGTACTTGCCGGGGCCGCACTACGACCCCCCGTCCAAGGAGAGGCTCAAGGCTCTCATGAATGAGTTTGACATTGACATGAACGGTCTCCTGGACCGTGAGGAGTTTGCGGAGTTCATCCGGAAGCTGACCGCCGAGTCACTCTGTGCGATCAGCCTCAAGCTGATCATCACGCTGGTCGCCGCCCCTGCGCTGGCGCTGGCGACCAAAAGGGCAACCGAGGGCGTCCCCGGCGTCGGCAAGGTAGTGCACAAGGTGCCCAATGCCATCTACGCCTCCGCCATCACCCTGGCCGCCGTGCTCATCCAGAGATCCGCCGAGGGCGTCGAGTAG

>HvCML25

ATGTCGAGCGGCGGGCAGAGGCAGCAGCAGGCGAAGAAGCCGTCGCCGGCTGCGGCGGGGGCGGACGAGATCGAGATCAAGAAGGTGTTCTCCCGCTTCGACACGGACGGGGACGGCAGGATCTCGCCCTCGGAGCTGGCGGCCGTGTCGCGCGCCATCGCGCCGCCGGCCACCGAGTCCGCGGGGGGGCGGGAGGTGGCGTCCATGATGGACGAGCTCGACACCGACCGCGACGGCTACGTGGACCTCGGCGAGTTCGCCGCCTTCCACGGCCGCGGCCGCGGGGAGCGCGAGCTGGACGCCGAGCTGCGCGACGCCTTCGACGTCTACGACATCAACGGCGACGGCCGCATCTCCGTCGCCGAGCTCAGCAAGGTCCTGTCCCGGATCGGCGAGGGATGCACCACCCAGGACTGCGAGAAGATGATCGCCTCCGTCGACGTCGACGGCGACGGCTGCGTCGGCTTCGAGGAGTTCAAGAAGATGATGGCCGGCGACGGCGCCGCACGGCCCCTAGACGGCGGCGTCCCTGACGACGACGGCAAAGCCAAGACGGAGTGA

>HvCML26

ATGACGCCGCCGCCGGCGGTCCCACCGGGGCGGAAGTCTCCAGCTGCGGTCCTCTTCCTCTGCTTGGTCACTACCTCATTACTCATGTTCATACTCCTTGCCTCCTACACCCCCCGCCTTGAGCCCCACGGCCGCAGCCCCCACCGCCGCCTCAAGCTCCACCCCAAGAACTCCGCTGCCGTCGCTTCTTCCTACGGGGCCGGCGCCGTCCACGAATCCGGAGGAAACCGCCACGCGGCGCCCTTCGACCCGGCCATCGCCGAGCTGGAGCGGCGGCTGGAGGACAAGGAGTGGGAGCGCGAGCACTACCGCATCCTCCATGGCGACGCCGAGAAGGGCGACCACATGAAGGAGTGGGAGGACTTCCTCAAGGAGGAAGAGGACTTCATCAACGACGACGATCGCTTCAACATCTCCGATCGCATCCGCGCGCTTTTCCCCAAGATCGACCTCAGCCCCGAGGACGGCTTCGTCTCCCTCGATGAGCTCATCAGGTGGAACCTCGATCAGGCCAGGGCCGACCAGCTCCATCGCTCCGCCAGGGAGATGGAGCTCTACGACAAGAATGGCAACGGAATCGTCTCCTTCACTGCTTTCCAGACGCTGCGCCAACAGTCCCATGGGGATGGAAACTCACTCGGCTTCCCGTGGTGGAAAGAGGAGCACTTCAATGCTTCGGACGTCAACGGGGATGGTTTCCTCAATAAAACTGAGTTTCACGACTTTCTTAATCCAAGTGATTCGGAGAATCCTAAAATTATCAACTTGCTCTGCAGACAAGAATTAAGGCAGAGAGATAAAGATGGTGATGGAAAGTTAAACTTCGAAGAATACTTTCATGGGCTACATGACCATATACATGGTTATGATGACGAGAATGCAGCTATTTCTCATATTGGGAACATGACAATCGCAAAGGAGCGGTTTTCCAAGCTTGACAAAGATAATGACGGATTCATTTCAGGGCATGAACTAGAACCTGTTCTTGATAAGCTCCATCTGTCAGAACGCTATTATGCCAGACAACAAGCCACACATGCTATTTCAGAGGCAGACAAAGATCATGATGGAAGGCTAACACTGGAAGAGATGATCGAGAACCCCTATGCATTTTATGGTAGTGTTTACTTTAGCGATGACGAGGACTACTTCCATGAAGAGTTCCGCTAA

>HvCML27

ATGAAACGACCTCGTCATCAGAAGTTGAAAACGAGATCCCGACGGAAAGGATGGTCAGATCTCGTGGCCACGCCACGCAACATGCAGAGCCATCGCCCCGCCCCCGAAGACCACGTCCGAGAAGAAAGCAGAAGCACACGCGCCCAACCAACAACCAGGCCGCACGCGGCGGCGACGAGGACAACCGCCACCCGTGCGAGACGCTCGGCATTCATGTCCGTCCCCTCCGACGCCGGCCAGGAGACACCGCCCTCGGCGACCGAGGGCGGTCGGGCGCGCCTCCAGGACGAGCAGCTGGGCCAGCTCCGGGAGCTCTTCCTGCGCTTCGACCTCGACGGCGACGGCAGTCTCACCAAGCTCGAGATAGCCGCGCTGCTCCGCTCGCTCGGTCTTCGCCCCGCCGCGGGGGACGAGATCCACACCCTCATCGCCTCCATGGACGCCGACGGCAACGGCACCGTGGAGTTCGACGAACTAGCCTCTTCCCTCTCGCAGCTGCTCCTCGGGCCCGGCCGCCCCGCAGTCGCCGTCGACCACGAGCAGCTCGCCGAGGCCTTCCGCGCATTCGACCGCGACGGCAATGGATACATATCCGCTGCCGAGCTCGCGCGCTCCATGGCGCAAATGGGGCACCCCATCTGCTACGCTGAACTCACCGACATGATGCGCGAGGCCGACACCGACGGCGACGGCTCCATCAGCTTTGAGGAGTTCACCGCCATCATGGCCAAGTCCGCCGTCGAATTTCTCGGCCTCGCCGCGTTGTGA

>HvCML28

ATGGCGAGCATGGAGGTAGCCAAGCCGGGGTCGTCCAAGCGGATGTCACCAAAGGGGAGCTTCAAGCTGAGCCTGCTTGCATGCGGCCAGTGCAAGGCCACCACCGTGTCGCCCCCGGACTCCCCCACCGGCGCCGGCGCGCGGTCGCTGTCTTCCTCGGCGTCGTCCTCCGCGGGGACGTCCCGCGACCGCCAGGCGGAACTCCGGGAGATCTTCCGTCACTTCGACCGCGACATGGACGGCCGGATCTCGGGCCGGGAGCTGGGCGAGTTCTTCGCGTCCATGGGCGACGGCGGCGCCAAAGCCGCGCTGGAGCTGGACGCGGCCGGCGGCGGCGACCTCATGCTGGGGTTCGAGGACTTCGTGAGGATCGTGGAGCGGAAGGGCGGGGAGGAGGAGGAGCGCGAGGACCTGCGGCGCGCGTTCGAGGCCTTCGAGGCGGTCAAGGGCTCCGGCCGGATTACGCCGCGCGGCCTGCAGCGCGTGCTCAGCCAGCTCGGCGACGACCCGTCCGTCGCCGAGTGCGAGGCCATGATACGCGCCTACGACGACGACGGCGACGGCGAGCTCGATTTCCACGACTTCCATCGCATGATGAACCATGACTAG

>HvCML29

ATGGCTACAATAGGTGAGTTCAGGCGCGTGTTCTCGGCGTTCGACCAGGACGGCGACGGCAAGATCTCCGCCGCCGAGCTGCAGCTCTGCATGAAGGCCGCGCTGGGCTGCGACATGTCGGTCGAGGAGGTGCAGTCGCTGATGGCGTCCGCGGACACCGACGGCGACGGGCTGTTGGACAAGGAGGAGTTCCTGAGGCTGGTGCTGGAGACCGAGGCCGGCAAGGAGGAAGAGGGGGACAGGTGCAGGGAGGCGTTCGGGATGTACGAGATGGAAGGGAGGGGCTGCATCACCCCGCTCAGCCTCCAGCTGATGATGAGCAAGCTGGGGCTGCACCTGGCCGTCGACGAGTGCCAGGCCATGATCCGCCGGTTCGACCTCAACGGCGACGGGGTGCTCACTTTCGACGAGTTTAAGACCATGATGATGATGGGATGA

>HvCML30

ATGGCGGACCAGCTCAGCGAGGAGCAGATTGGCGAGTTCAAGGAGGCCTTCAGCCTCTTCGACAAGGACGGCGACGGTAGCATCACCACCAAGGAGCTTGGAACCGTGATGCGCTCCCTTGGCCAGAACCCTACCGAGGCGGAGCTGCAGGACATGATCAACGAGGTGGATGCGGACGGCAACGGCACCATTGACTTCCCAGAGTTCCTGAACCTGATGGCCCGCAAGATGAAGGACACCGACTCGGAGGAGGAGCTCAAGGAGGCGTTCCGTGTCTTTGACAAGGACCAGAACGGCTTCATCTCCGCGGCGGAGCTCCGCCAGGTGATGACCAACCTCGGGGAGAAGCTGTCGGAGGAGGAGGTGGAGGAGATGGTCCGGGAGGCGGACGTGGACGGCGACGGCCAGATCAACTACGACGAGTTCGTCAAGGTCATGATGGCCAAGAGAAGGGATAAGAGGGTAGAGGAGAGGAGGGCGCCGCCGGCCAGGAAGAGCGCGGCAGGGGCATCGCCATCCGGTGCAAAGAGTGGCAACAAATGCATAATCCTCTAG

>HvCML31

ATGAAGCTCTCCATGCAGTCGCTGGCCCGGAAGCTCTCCATCCCGTCGCCCAAGCGGGGCAAGAAGCAGCAGCAGCAGGAGGAAAGCGGCAAGCGGGGCATCTCCCGGAGCGAGGCGCCGTCGTTCGCGTCGGCCTCGTCCTCCTCCACCGCCTCGTCGTCTGCCTCCGAGGACGCGCCGGCGAGGGCGTCCACGCCGCGGTCCGTGCTCCCCGCCGAGATCTCGCGGCGGGAGCTGGAGGCCGTGCTCCGGCGGCTGGGCCACGAGGAGCCCTCCGACGACGAGCTCGACGCCGTGGCCGCCATCGCCGCCGCCGGCGAGGCCGGCCCCGAGGACGAGCTCATGGAGGCGTTCAACGTGTTCGACGCCGACGGCGACGGCCGCATCACCGCCGAGGAGCTCCGCGGCGTCATGGTCGCCATCCTCGGCGGCGAGGCCGACGGGTGCAGCCTCGACGACTGCCGCCGTATGATCGGCGGCGTCGACGCCGACGGCGACGGCTTCGTCGGCTTCCAGGACTTCGCGCGCATGATGATGGTGTCCACCACCGCGGCGGCCGGCCCGAGATTCCTGTGA

>HvCML32

ATGGGCAAGATCAAGATGCCGTCGCTGTTCCGCCGCCGCTCGTCCTCCAAGTCGCGGTCTTCCTCGCCGCCGCCGCCGCAGCAGCAGGAGGAGGAGGGGGACCGGGCGGCGTCGGGCGCGGGGTCCCCGGCGCGGACGGCGGAGGAGGAGATGGAGCGGGTGTTCCGCAAGTTCGACGCCAACGGCGACGGCCGGATCTCGCGGCCGGAGCTGGCGGCCCTCTTCGAGAGCCTGGGCCACGCGGCGACCGACGACGAGCTGTCCCGCATGATGGCGGAGGCGGACGCGGACGGCGACGGCTTCATCAGCCTGGCGGAGTTCGCGGCGCTGAACGCGACCGCGGCGGGCGACGACGAGGAGGACCTGCGGCTCGCCTTCAAGGTGTTCGACGCGGACGGCAGCGGCGCCATCTCCGCCGCCGAGCTGGCGCGCGTGCTCCACGGCCTCGGCGAGAAGGCCACGGTCCAGCAGTGCCGCCGCATGATCGAGGGCGTCGATAAGAACGGCGACGGCCTCATCTCCTTCGACGAGTTCAAGGTCATGATGGCCTCCGGCTTCGCCGCCAAGATGGCATGA

>HvCML33

ATGGTTGCGGCAAAGTCGGCCGAGCTGAGGGCGCTCTTCTTGTCACTGGACCGGGACGCAGACGGCAGGATCTCTCCGGCGGAGCTGCAGGGGTGCATGCGCGCGACGCTGGGCGAGGACGTGCCGGCGGAGGAGGCCGAGGCGCTGGTGGCGTCGGTGGACGCGGACGGTGACGGGCTGCTAAGCGAGTCCGAGTTCCTCGAGCTGGCGCAGCAGGCGGACGCGGGGGAGGAGGACGGCGAGCGGAGGATATGGGCGCTGAGGGAGGCGTTCGGGATGTACGAGATGGAAGGGCTGGGGTGCATCACGCCGGCTAGCCTGGCGCGGATGCTCGGCAGGCTCGGCGCCGAGCGCGGCGCCGGCGAGTGCCGCGCCATGATCTGCCGGTTCGACCTCAACGGTGACGGCATGCTCAGCTTCGACGAGTTCAAGATCATGATGAGCTAG

>HvCML34

ATGGTTGCGTCCAAGTCCGGCGAGCTGAGCACGCTGTTCGCGTCCCTGGACCAAGACGCGGACGGCAGGATCTCCGCGACGGAGCTGCGGTTGTGCATGCGGGCGACGCTGGGAGAGGACGTGCCGGCGGAGGAGGCCGAGGCGCTGGTGGCGTCGGCAGACGCCGACGGAGACGGGCTGCTAAGCGAGTCCGAGTTCCTCGAGCTGGCGCAGCAGGCAGCCTGGGCGGGCGACGCGGGGGAGGAGGACGACGAGCGGAGGATCCAGGCGCTGAAAAAGGCGTTCGGGATGTACGAGATGGAGGGGCAGGGGTGCATCACGCCGGCCAGCCTGGGGCGGATGCTCGGCAGGCTCGGCGCCGAGCGGGGCGCCGGCGAGTGCCGTGCCATGATCTGCCGGTTCGACCTCGACGGCGACGGCGTGCTCAGCTTCGACGAGTTCAAGATCATGATGAGCTAG

>HvCML35

ATGGTTGTGTCAAAGTCGGGAGATCTGACGGCGCTCTTCTTGTCACTGGACCGGGACGCGGACGGCCTGATCTCTGCGGCGGAGCTGCTAGGATGCATGCGCGCAACGCTTGGCGAGGACGTGCCGGCGGAGGAGGCCGAGGAGCTGGTGGCGTCGGTGGACGCGGACGGTGACGGGCTGCTAAGCGAGTCCGAGTTCCTCGAGCTGGCGCAACAGGCGGCCTGGGGAGGCGATGCGGGGGAGGAGGACGACGAGCACAGGATCCGGGCGCTGAGGGAGGCGTTCGGGATGTATGAGATGGAGGGGCAGGGGTGCATCACGCCGGCCAGCCTGAGGCGGATGCTCGGCAGGCTCGGCGCTGAGCGGGGCAGTGGCGAGTGTCGCGCCATGATCTGCCGGTTCGACCTCGACGGTGACGGTGTGCTTAGCTTCGACGAGTTCAAGATCATGATGAGCTAG

>HvCML36

ATGGTGCACGCCGCGACGGCCGAACGCTTCAGCAGCTTGTTTGCCTCATTCGACCGCGACGCCGACGGCAGGATCTCGGCGGTGGAGCTGCGGCTGTGCATGAAGGCAGCGCTGGGTGAGGAAGTGTCGGCGGAGGACGCCGAGGCGCTCGTGGCGTCGGCCGACGCCGACGGTGACCGGCTGCTGGACGAGCAGGAGTTCCTCCGGCTGGTGGCGCCGCCGGAGACGGAGGAGGAGGAGCGGTGCAGGGGGCTGAGGGAGGCGTTCGCGATGTACGAGGTGAAGGGCGAAGGGTGTATCACGCCGTCGAGCCTGATGCGGATGCTCGCCAGGCTGGGGTCCGAGCAGGGCATCGAGGAGTGCCGCGCCATGATCCACAGGTTCGATCTGAATGGAGATGGAGTGGTTTGCTTCGACGAGTTTAAGGTTATGATGGATGCGTAG

>HvCML37

ATGGCTGGCTACCCCCCGCCCCCCGGCTCCGGCTACCCCTACGGCGCTGCTGGCGGCTACGGAGCCCCGCCGCCCTCCGGCCAGAAGCCCCCCAAGGAAGGCAAGACCTCTTCCTCTTCCGGCCCCGACCCCTACCACGGCGCCCCGCCCCCGCAGCAGCCTTACGGCGGGGGCGGTGGCGGCGGCTACGGCGCCCCACCGTCCTACGGCCAGAAGCCCCCCAAGGAAGGCAAGACCTCCTACTCCTCCGGCTCCGACCCCTACCACGGTGCTCCGCCCCCGCAGCAGCCTTACGGCGGGGGCGGCGGAGGCGGCTACGGGCAGCAGCCCTACGGCGCCCAGCCGCCTTCGTCCGCCGCGCCGTACGGGGGCCCACCCGCCGCGCAGCCCTACGCAGGAGGCGGCGCCGGCGGGTACGGTAGCCCGTTCGCGGCGCTGGTGCCGTCGACCTTCCCGCCGGGGACGGACCCGAACGTCGTGGCGTGCTTCCAGGCGGCGGACCGCGACGGCAGCGGGATGATCGACGACAAGGAGCTGCAGTCCGCGCTCTCTGGGTACAGCAGCCAGAGCTTCAGCCTCCGCACCGTCCACCTCCTCATGTACCTCTTCACCAACTCCAACGTCCGCAAGATCGGGCCAAAGGAGTTCACTTCTGTGTTTTACAGTCTTCAGAATTGGAGGGGCATATTTGAGAGGTTTGACCGTGACCGAAGTGGTAGAATTGATGCAGCAGAACTGCGTGATGCTCTTCTAGATCTTGGATATTCGGTCTCTCCAACTGTGCTAGACTTGCTTGTGTCTAAATTTGACAAGACTGGGGGCAAGAACAAAGCGGTTGAATATGACAACTTCATTGAATGCTGCCTTACAGTGAAGGGCCTGACCGAGAAGTTCAAGGAGAAGGACACGGCGTACTCGGGGTCTGCAACTTTTGGCTACGAGGCGTTCATGCTAACCGTGCTCCCTTTCCTCATCGCATGA

>HvCML38

ATGGATGAGCTGTCCAAGGAGCAGATCGACGAGTTTCGGGCGGCCTTCAGCCTCTTCGATAAGGATGGAGACGGGACGATCACGGCCAAGGAGCTCGGCACGGTGATGCGGTCGCTGGGGCAGCGCCCGTCAGAGGAGGAGCTGAGGGAGATGATCGCGGAGGTGGACGCCGACGGCAACGGCGTCGTGGACTTCTCCGAGTTCCTGGTCCTCCTCGACCGCAAGATGCGCGGCGCCGACGCCGAGGACGAGCTCCGCGAGGCCTTCCGCGTCTTCGACCAGGACCAGAACGGCTTCATCTCGCTCGACGAGTTCCGGCACGTCATGGACAACCTCGGCGAGCGCCTCTCCGACGAAGAGCTCAAGGAGATGCTCCGCGAGGCCGACCTCGACGGCGACGGCCAGATCAACTACTCCGAGTTCGCCAGGGTCATGATGGCCAAGATGGACTCGACGGGATCAAAACCATTGGTGCCGCTGCTCTTCAAGAATGGAGTCCGGGTCTGCCGCTACCTTCTGGCCTTCCGGATTATGGAGATCGGCCGGAGTATTAAACGACGACGGCGGAAGTCACAGCAAAACAGTCGTGAGATGGTAGGAGAGAAGACCGGCGGCGATGCCGATCCCCCGCCGGAGCAAGGAGACAAGGACAAACGAGGAAGCGACAGCCGCTGCATCCCCTCATGTACCATCCTCTGA

>HvCML39

ATGGACACCGCTCCTGCTCCTGCTCCCGCTCCCACGAAGCCGTCCTTGTCCAAGAAGCCCTCGCCGTCGTTCCGCCTCCGCAACGGCAGCCTCAATGCCCTGCGCCTGCGCCGCGTGTTCGACCTCTTCGACCGCAACGGCGACGGCGAGATCACCCTCGACGAGATGGCGGCCGCGCTCGACACGCTCGGCCTCGGCGCCGACCGCGCCAGCCTGGAGGCCACCGTGGGCGCCTACATCCCGGCTGGCGCCGCCGGCCTCGGCTTCGAGGACTTCGAGGGCCTCCACCGCGCTCTCGGCGACGCGCTCTTCGGCCCCATCGCCGAGGAGGAGCCCGGCAAGGAAGGCGAGGCCGAGGACGAGGACATGAAGGAGGCGTTCCGGGTGTTCGACGAGAACGGCGACGGGTTCATCTCGGCGGCCGAGCTGCAGGCCGTGCTCAAGAAGCTGGGCCTGGCGGAGGCGCGCAACCTGGCGGCGGTGCAGGAGATGATCTGCAACGTCGACCGCGACCGCGACGGCCAAGTTGACTTCGGCGAGTTCAAGTGCATGATGCAGGGGATCACCGTGTGGGGAGCTTGA

>HvCML40

ATGTGTCCCGGCGGCAGGTACGCGGGCCTTGACCTCCCCGCCGGCGCCGGAGCGGGGGACCTGCGGCCGGCATTCGACGTGCTTGACGCGGACCACGACGGCCGCATCAGCCGCGACGACCTCAAGTCCTTCTACGCCAACGCCGGCGCAACCGACGAGCGCTTCGACGACGACGACATCGAGGCCATGATCGCCGCCGCCGATGCCGACCTCGACGGCTTCGTGCAGTACGACGAGTTCGAGGGCCTCCTCGGCCGCGCCGCCAAGGCGGGGACGGACGGCGGGTGCCGCTCCGCGATGGAGGACGCCTTCCGGCTAATGGACCGCGACGGGGACGGCAAGGTCGGCTTCGAGGACCTCAAGGCCTACCTCGGGTGGGCCGGGATGCCGGTCGCCGACGACGAGATCCGCGCCATGATAAGTATGGCTGGTGACGGCGACGGCGGCGTGGGGCTCGAGGCGCTCGCCAGAATACTCGCCGTGGACTTTGGTGCCATCGTCTGA

>HvCML41

ATGGAGAGGGGCTGGTTCGGGTGGAGGAAGGCGAAGCAGGGCGGGGGGAAGGAGGAGGAGGGTCGGGCCAAGGTGGTGGTGGACGGGTCCGGGATACGGCAGCTGGTGGAGGACCGGGAGGCGTTCGGCATGTTCGCGGAGACCAAGTTCCGGCAGCTCGACGCCGACGGCGACGGCCGGCTGTCCGTGCGGGAGCTGCAGCCCGCCGTGGCCGACATCGGCGCCTCGCTCGGCCTGCCCGCGCAGGGCTCCTCGCCCAACGCCGACCACATCTACTCCGAGGCTATGAGCGAGTTCACTCATGGTCACGGGAACCAGGAGGGGGTGAGCAGGGCAGAGTTCCAGGAGGTCCTGTCCGACATTCTCCTCGGCATGGCGGCCGGGCTCAAGAAGGACCCGATCGTGATACTGCGCATCGACGGCGAGGACCTCAGGGACTTCTTGTCCAGCCCAAGGTATGAGCCGGTAGCCGCTGCCATCTTCTCACAGGTTGGGTCTGAAGATGCCCCTCTCCGGCAATGCCTGCTAGCCGCCGTTCAGCAGCTTGGCGTCGACCACGGAATGCCCCCAGCCGCTGACGCTTGGGTGGTAGAGAACGTGGTAGAGCCGGCATTGCAGCAGCTCCCCGCCGATGAGCTCGAGCGACGGGCGTCCCGGGACGTCTTCTTGGAGCAGCTGAAGAAGCTGCTGGCCGGCGTCGCGGAGCAGCTTCAGGAGCGGCACGTGATCGTCGCGCACACGGAGAACACCTTCGACGGGAGCGGCGTCAGGAGGCTGCTGGGTAACAAGTTCGAGCTCGACAAGCTGCTGGATTCTGTGTGGAGGGAGGTGCCGGCAGAGCACAGGAAGAAGGCGCCCAAGGACAAGGAGCACCTGCGGGTCGCGCTCGATAAGATGGCCGACGCAGCAAGCTTGCCACCCCATGGCGCTGTTGATCGGGTGGACGCCGTCGTGGACGAGGCGCTCAAGGTGGCGGACGCCGGCGACGGGAAGGCGGTCGAGGAAGCGGAGTTCAAGAAGCTGCTGACGGACGTCCTTGGGGCCGTGATGCTGCGGCTGAGCGGCGAACCGATCTTCTTCTCCACCAGCACCGTCGTCCACGAGTCGATGCCCGGCTCGTCCGCCCTGTTGCCGTCGCCCGCGGTGGCATCTCCGCCGAGCGAGTAG

>HvCML42

ATGGAGAACGCGGTGGTGCTCCGGGAGTGGTTCGACCGCGTCGACGCCGCCGGCACCGGCAACGTCACGGCTCCTCAGCTCCAGAGCGCGCTGGCCGTGGGCAACCTCGACTTCCCGCTCTCCGTCGTGCAGCAGATGATCAGGATGTACGACTTCGATCGGAACGGCACCATGAGCTTCCAAGAGTTCTTGGCTCTTAACAAGTTCCTTCACAAGGTGCAGGGTGTCTTCTCCACCCTAGAAAGGGGTCGTGGATTTCTCAGTCTTGAGGATGTGTATGAGGCATTAATCAAACTCGGTTTCTCTTTGGATTCACCTGCCTTCTACACTGTCTGTGAGAGCTTCGACAGGAGCAAGAAGGGGATGGTTCGTTTGGATGAGTTTATATCGATCTGCATCTTTGTTCAGTCAGCTCGTAACCTGTTCAGTTCATTCGACACAACCAAGCAAGGGAAAGTGACCTTCGATTTCAACCAGTTTGTCTACTGCACGGCGAACTGCAGGATATAG

>HvCML43

ATGGCGGGCGCCGGCGCAACAGCAGGGATCAGCTCGGAGCAGATGAGCGAGTTCCGGGAGGCGTTCGCCTTCTTCGACAAGGACGGGGACGGCTGCATCACGGCGGAGGAGCTGTCCACGGTCATCCGGTCCCTGGGCCAGACCCCGACGCCCGAGGAGCTGCGGGACATGGTGCGCGACGTGGACGCCGACGGCAACGGCACCATCGAGTTCGCCGAGTTCCTCGCGCTCATGTCACGCAAGGCCGACGCCGACGCGGACGCCGCCGACCCCGAGGAGGAGCTCCGGGAGGCCTTCAGGGTCTTCGACAAGGACCACGACGGCCACATCTCCAAGGCCGAGCTGCGCCACGTCATGATCAGCCTCGGCGAGAAGCTCACCGACGACGAGGTGGACGGGATGATCCAGGAGGCCGACCTCGACGGCGACGGCCTCGTCAATTTCGACGAGTTCGTCAGGATGATGATGCTCTCCGACGCCGACCAGCACCAGCATTGA

>HvCML44

ATGGCTGCCGGTGCCGCAGCAGCGATCAGCTCGGAGCAGATGAGCGAGTTCCGGGAGGCCTTCGCCTTCTTCGACAAGGACGGGGACGGCTGCATCACGGCGGAGGAGCTGTCCACGGTCATCCGGTCCCTGGGCCAGACCCCGACGCCCGAGGAGCTGCGGGACATGGTGCGCGACGTGGACGCCGACGGCAACGGCACCATCGAGTTCGCCGAGTTCCTCGCGCTCATGTCACGCAAGGCCGACGCCGACGCGGACGCCAGCGACCCCGAGGAGGAGCTCCGGGAGGCCTTCAGGGTCTTCGACAAGGACCGCGACGGCCACATCTCCAAGGCCGAGCTGCGGCACGTCATGATCAGCCTCGGCGAGAAGCTCACCGACGAGGAGGTGGAGGAGATGATCCAGGAGGCCGACCTCGACGGCGACGGCCTCGTCAACTTCGACGAGTTCGTCAGGATGATGATGCTCTCCGAGTCCGATCAGCAGCAGCACTGA

>HvCML45

ATGAAGAAGGTGTTCTCCCGCTTCGAAACGGACGGGGACGGCAGGATCTCGCCCTCGGAGCTGGCGGCCGTGTCGCGCGCCATCGCGCCGCCGGCCACCGATTCGGCAGGGGGCCGGGAGGTGGCGTCCATGATGGATGAGCTCGACACCGACCGCGACGGCTACGTGGACCTCGGCGAGTTCGCCGCCTTCCACAGCCACGGCCGCGGGGAGCGCGAGCTGGACGCCGAGCTGCGCGATGCCTTCGACAGCGCATACACGGCGGTGCGCCTCGAGTGGATGTGGTGGGAGGAGGTGAGGAGAAGGGAACGCGGGGAGCTGCCCGCGCGGCGCTTCGAGGCCCTCGCCCGCTCACGCCGCGCCGCCTCGCTCGCGCTCTCCAACCGCAAGGAGATCGCCACCTCGCACCTCGGCGCCGTCAACTTCCTCCAGGAAGTGACTTATGATGCATTGACTCAAGAAATAGAAGAGATCTTTCTATTTTCTGAAGAGACCTGTGCACAATATAGCTGGGTACTATTATACCTGAAGGGTTACCATTCCTTGCAGTCCTATAGGATAGCTCATGTATTATGGAATCAAGGGCGTAAGGTTCTGGCGTTGGCACTGCAAAGCCGTATCAGCGAGGTTTTTGCAGTGGATATACACCCAGCTGCCAAAATTGGGGAAGGAATATTGTTGGATCATGGAACAGGTCTAGTCATTGGTGAAACTACTGTTGTTGGCAATTGGGTTTCATTAATGCAGGTTGTGGTGTGGTTCAGCACCGGCTACATCACCATAAAGGACATGGTGGTCACCGGCATGCCCATCAAGATCGTCGGCCTTGCCGCTCTCAAGGTCCTGTTATTTTCTGAAGTCATGTGTGCTTGCACTTATGATGCATTGACTCAAGAAATAGAAGAGATCTTTCTATTTTCTGAAGTCAATCTCAATTGTTCACCTATCCTTTTATACATGAGTAGAAGTCCTAATAAGAGAAAATAA

>HvCML46

ATGAAGTTCTTCGGTTCCTCCACTTCCAAGAAGGAGAACAAGGGGAAGAAGAGATCCAAAAGGAGCGGCAACAGCGGTTCCTTCGCCTCCACCGCGTCGTCGTCGGCATCAGATGAGCAGTCCGTCACAACGCCGAGTTCCGTCCCGCCGTCGTCTTCGGGGACGGCGGCACCGTCCGGCTGCGGGACGACCAAGAAACCGGCCATGGTCGCAGTGACGCGGCTGGAGCTGGAGGTCGCGTTGCGTACAGTCGTGTCGACGGAGGAGGAGCTGGCCGTGATGCTCGCCGAGGCGGAGGCCGGCCTCGCGCTTGATGGGGTCGAGGACGGCGAGGCCGCTGACGAGGGCGAGCTGAGGGACACATTTGCGGTCTTTGACGCTGACGGGGACGGGAGGATCTCCGCCGAGGAGCTCCGTGCCGTGTTGGCCACCCTCGGCGACGAGCGGTGTTCCGTCGAGGACTGCCGCCGCATGATCGGCGGCGTCGACAGCGACGGCGACGGCTTCGTCTGCTTCGACGAATTTACGCGCATGATGATGCTTGCCCTGTGA

>HvCML47

ATGGCCTGCGCGTCGAGAGTCATCATTTTGCCCCCATCACCCGTCTCGAGTCATAAAAACTGGTGCCAGCCTTCATTCACCGTTCGAAAGCCTCCCTCCCCACCCCGCCGCGATTCCCCGCACCCAATTCCCCAAGCACCGCCGCCCAAGCTCGATCGCCCGACCATGTCCGGCTACCCCTACGGCGGCGCCGGAGGAGGAGGAGGCTACGGGGCCCCTCCGCCCTACGGCTCCTCGCCCGCCCCCTCGGCCCCGCCCTACGGCGACAAGCCCCCCAAGGAAGCCAAGGCCTCCTCCCCCTACTACGCCTCCCCGCCCCAAGCCTACGGCGCCGGGGGAGCCGGAGGCGGAGGCGGAGGCTACGGGGCCCCGCCCTCCTCCCAGCCCCAGTCCTACGGCGGCGGCTACGGGGCCCCGCCACCCTCCCAGCCGCAGTCCTACGGCGGCGGTGGTTACGGGGCCCCGCCTTCCTCCCAGCCCCAGTCCTACGGCGGCGGCGGCTACGGGGCCCCGCCATCCGCCCAGCCCTACGGCGCCCCGCCTCCCTCGTCGGCGTCCTACGGGGCCCCGCCCCCCGCGGCAGCGCCGTACGGGGCCGCTGGCGGCTACGGGAGCCCGTTCGCGGCGCTCGTGCCGTCCGCGTTCCCGCCCGGGACGGACCCCAACGTCGTGGCGTGCTTCCAGGCCGCCGACCGCGACGGCAGCGGGACCATCGACGACAAGGAGCTGCAGTCCGCGCTCTCCGGCTACAACCAGAGCTTCAGCATCCGCACCGTCCACCTCCTCATGTACCTCTTCACCAACACCAACGTCCGCAGGATCGGGCCCAAGGAGTTTACTTCTGTTTTTTACAGTCTTCAGAATTGGAGGTCTATATTTGAGAGGTTTGACCGTGACCGAAGTGGTAAAATTGATGCATCGGAGCTGCGTGATGCTCTTCTCAGTCTGGGTTATTCAGTTTCTCCAACTGTGCTAGACTTGCTCGTGTCTAAATTCGACAAGACTGGGGGCATGAGCAAAGCAGTCGAATATGATAACTTTATTGAGTGTTGCCTTACAGTCAAGGGTCTGACTGAGAAGTTCAAGGAGAAGGACACGGCTTACTCCGGGTCTGCAACTTTCAGTTACGAGGCATTCATGTTGACTGTGCTCCCTTTCATCATTGCATGA

>HvCML48

ATGGACGAGCTTTCCAAGGAGCAGATCCAGGAGTTCCGGGAGGCCTTCAGGCTCTTCGACAAAGATGGCGACGGGACGATCACGACCAAGGAGCTGGGGACGGTGATGCGGTCGCTGGGGCAGCACCCGACGGAGGAGGAGCTCAAGGACATGGTGGAGGAGGTGGACGCCGACGGGAGCGGCTCCATCGACTTCAACGAGTTCCTGGGGCTGGTGGCGCGGCAGATGCGCGGGGACGCCGACGCCGAGGAGGAGCTCCACGAGGCCTTCCGCGTCTTCGACAAGGACAACAATGGCTTCATCTCCCTCGACGAGCTCCGCACCGTCATGAAGAACCTCGGCGAGAAGCTCTCCGAGGACGAGCTCAACGAGATGCTCCAAGAGGCCGACGCCGACGGCGACGGGCAGATTAACTACAAGGAGTTCGCCAAGGTCATGATGGCAAAACGGCGAGCAAATGCGGAGGAACATGGAGGTGGCGATCACGGCGGGTCAGATCACGCACACAGCGGTGGCGGAGGCTGCCCATGTACAATTCTCTAA

>HvCML49

ATGGTGGCCGATGCCGTGGTGCAGGCGTCGTCGGTGTTCGCCGCCTTCGACAAGGACGGCGACGGCAAGGTGTCGGCCGCCGAGCTGCGCGGCTCCATGACGGCGGCGCTGGGCGAGGAGGTGTCCGAGGAGGAGGCCGCGGCGATCCTGGCCACGGTGGACGCCGACGGCGACGGGCTGCTCGACCAGGAGGAGTTCTCCCGGCTCGGCCTCGGCGCCACCGGGGACGACGCTGGGGCGGCCGGAGCCGACGACGAGGAGGAGGTGAGGCGGCGGTGCCTGAGGGAGGCGTTCGCGATGTACGCCACGGAGGGCGGCGACGAGCGCGCCAGGATCACGCCGGCGAGCCTCAGGCGGATGCTGGGCAAGCTGCTGGGGTCCGAGAAAATGGGGCTGGAGGAGTGCAGGGCCATGATCTGCAGGTTTGACCTCAACGGCGACGGCGTCCTCTCCTTCGACGAGTTCAGGGTCATGATGATGGCCAATTTGTAA

>HvCML50

ATGGACATGGAGATGGCGGCATCTTCCTCGGCGCCGGGAGCGTCCTACTTCAGCTTCAACTTCTCCGTGGCGCAGGCCGTCGTGACCATCTCCATCAACGTCGTCGTCGTCTGGCTCTCCGCCCTCGTCAAGTCTTCCTCCTCCTCCTCTTCGTCCTCCGCCAGCAGCCGCTCGCCCGCCCCCGCGCCGGAGCCGGAGCCAGCGCAGCCTGCCGCGGCAAGTGGCGCCTCGGAGGTCGACCTGGACGTGGTTCTCGGGGTGATGGGCGCGGCCGGCGCCGCCTCGGTCGGGTTCGAGGAGGCGGCGGCGCTGTTCGAGGAGGAGGAGGCGACCGTGGAGGAGGCAGCGGCGGCGTTCCGCGTCTTCGACTGCAACGGCGACGGCTTCGTCGACGCCGGAGAGCTCGGGAGCGTGCTCAGGTCGCTCGGGTTCACCGCCGGCGTCGCGGCCGCGGAATGCCAGCGCATGATCGATGCCTATGACGAGAACAAGGACGGCCGGATGGACTTCCAGGAGTTCCTCAGCTTCATGGAGAGGAGCAGCTCGTGA

>HvCML51

ATGGTGCACGCCGCGACGGCCGAGTGCTTCAGCAGCGTGTTCGCCTCATTCGACCGCGACGCCGACGGCAGGATCTCGGCGGCGAAGCTGCGGCTGTGCATGAAGGCGACGTTGGGCGAGGACGTGTCGGCGGAGGACGCCGAGGCACTCGTGGCGTCGGCCGACGCCGACGGATACCGGCTGCTGGACGAGCAGGAGTTCCTTCGGCTGGTGGCGCGGCCGGAGACGGAGGAGGAGGAGGGGCGGTGCAGGGGGCTGAGGGAGGCATTCGCGATGTACGAGGTGAAGGGCGAAGGGTGCATTACGTCGTCGAGCCTGATGCGGATGCTCGCCAGGCTGGGGTCCGAACAGGGCATCGAGGAGTGCCGCGCCATGATCCGCATGTTTGATTTGAATGAAGACAAAGTGGTTTGCTTCGACGAGTTCAAGGTTATGATGGATGTGTAG

>HvCML52

ATGTCGCACCTGAGCATCCTGACGTTCAAATACAACCTAGCGAAGCTCCGGTTCAAGCCCGCCCGGCCGACCGGACGGCTGCTCTCCTCCAGGGACCGGCAGCAGTCCGACCTGATGATGTACAAGCCTGATGACGAGGAGATGGCCAAGGTGTTCGACAAGATCGCCGGCGAGCCTGGCCTGATCTCGAGGAGCGACCTCCAGGCGCTCCTGCAGAGGTTTGACAAGGCCGATGCCGTGGGCGAGGCGCGGCGGATGGTCTGCGCCGCCGACAGCAACAAGGACGGGTACATGGACCTGGAGGAGTTCATGGAGGTGCACAAGAACGGCGTCCTGCTAGGGGACATACGCCGGGCCTTCTTCGTGTTCGACAGGGACAGGGACGGCAGGATCACGGCCGAGGAGGTGATGGACGTCCTGCGCAAGCTCGGGGACAGCTGCAGCCTCGAGGACTGCCGGAAGATGGTGAAGGAGATCGACAGGAACCATGATGGGTACGTCGACATGGATGACTTCATGGCCATGATGACTCGCCCAAGGAAGAGGATGTAA

>HvCML53

ATGTCACTTCTGCACTGCTTCCTGAAACCGTCCAGGATGAGCCTGCAGCGGTGCGTCGTCGGCGACGACATGACGGCGGCCGAGTTCAAGGACTGGATCCGGCAGTTCGACGCGGACCAGGACGGGCGCATCAGCCGCGCCGAGCTGCGGCGCGCGATGAGGGCGCTGCGCGTGCGGTTCACGCGGCGGAGGAGCAGGAGCGGCATCAGCTACGCCGACGCGGACGGCGACGGGTACATCGACGACAGCGAGATCGACGGCCTCGTCGAGTTCGCGCGGACCAACCTCGGCCTCCGGATTGTCGCCTGCTAG

>HvCML54

ATGGCGATCAGGAACGTGACGGCGGCGACGCGGTCGCTGGACGGGGACATGACGGTGGACGAGTTCAAGGAGTGGCTCCGGCGGTTCGACGTGGACCGCGACGGCCGCATCAGCCGCGACGAGCTGCGGTGCGCGATGCGCACCATCCGGACGCGCTTCTCGGGGTACAAGAGTAAGCGTGGCATCGAGTACGCCGACACCGACGGCGACGGTTACGTCGACGACGGCGAGGTGGACGGCCTCATCGAGTACGCGCAGAGGAGCCTCGGGCTCAGGATCGTCGCCTACTAG

>HvCML55

ATGAGCGTGGAGATCCTGGACGGGCTGACGGTGCAGAGCTTCGTGGAGGACGAGGGCGCCTTCAACGCCTCCGTCGACGGCCGCTTCGCGGCGCTCGACGCCAACCACGACGGCCTGCTCTCCTACACCGAGATGGCCGGGGAGCTCATGAGCCTCCGGATCCTGGAAAAGCACTTCGGCGTCGACGAGGCCGGTGCCGATGGCCCCGAAGAGCTCGCGGGGCTCTACCGCGGCCTTTTCGCGCACTTTGACCGCGACGGCGATGGGGCGGTGGACCGCGAGGAGTTCAGGGCGGAGATGAAGGAGGTCATGCTCGCCGTGGCCAGCGGGCTCGGGTTCCTGCCGGTGCAGATGGTGGTCGAGGAAGGGAGCTTTCTCAAGGTGGCTGTGGACAGGGAGCTGGCCAGAGCGGCCTGA

>HvCML56

ATGAGTGTAGAAATCCTTGATGGCAAGACCGTCCAGAGCTTCGTCGAGGACGAAGACGCCTTCAACTCATCGGTGGACGGCCGGTTTGCGGCCCTCGACACCAACCATGATGGCTTGCTCTCGTACTCCGAGATGGCCCAGGAGCTCATGAGCCTCAGGGTTCTTGAGAAGCACTTTGGGGTCGATGAGTCCGCCATGAGCCATGGTGAGCTTGTCGAGCTCTACCGTGGCCTGTTTGCGAGGTTCGACCGAGACGGCAATGGCACGGTGGAACTCGAGGAGTTCCGGGCTGAGATGAAGGAGGTGATGCTCGCTGTGGCTAATGGACTCGGCTTCCTGCCGGTGCAGATGGTGGTTGAAGAAGGCAGCTTTCTGAAGGTGGCTGTGGACCGCGAGCTGGGCAAAGCAGCTTGA

>HvCML57

ATGGGGAACGCGTCGTCGATGCTGACGCAGTACGACATTGAGGAGGTGCAGGAGCACTGCAGCTACCTATTCTCGCAGCAGGAGATCGTGTCGCTGTACGAGCGATTCTGCCAGCTCGACCGCAGCGCCAAGGGCTTCGTCGCCGAGGACGAGTTCCTCTCCATCCCGGAGTACTCCACCAACCCGCTCTCCCAGAGGTTGCTACGTATGGTTGATGGATTGAACTTCAAGGAATTTGTTTCGTTTCTCTCTACCTTCAGCACAAGGGCCAGCTTTCAGCAAAAGATTGAGTTGATGTTTAAGGTGTATGACATTGATGGCAAGGGGAAAGTAAGCTTCAAGGACCTGGTAGAGGTTTTACGGGACCTAACGGGCTCATCCATGTCAGAGCAACAAAGAGAGCAAGTACTAACAAAGGTGTTGGAAGAAGCTGGGTACACACGGGATTCCACTCTATCATTAGAAGATTTTGTGACGATCATCGACCACCCTGGCCTGAAAATGGAGGTGGAAGTGCCCATCGACTAG

>HvCML58

ATGTCCACGGCGCCGCCGCAACCGAACCAACAGCAGCAGCGCCACCGGAGGCTGCGGGTGCGGCGTGTGTTCGACCTCTTCGACAACGACGGCGACGGGGTCATAACCGCCGGCGAGCTCTCGGGCGCGCTGGGACGCCTGGGCCTCGCCCTGGGCGCGCAGGCGGACGTGCTCATCGCCGAGTACGTCGCACCCGGCATGCCCGGCCTGCGGTTCGCGGACTTCGAGGCGCTCCACGCCGAGCTCGCCGGCGGAGGCGAGGAGGACGAGGAGGCGGAGATGAGGGAGGCTTTCGCCGTGTTCGACGAGAACGGCGACGGGTACATCTCCGCGGCGGAGCTGCAGGCCGTGCTGGCGCGGATGGGGGTGCCGGAGGCGGCGTGCATGGCGCGGGTGCGCGACATGATAGCCGCGCATGACCAGGACAGCGATGGCCGCGTCGACTTCCACGAGTTCAAGGCTATGATGGCTGCCGCATAA

>HvCML59

ATGGAAGAAGCGCTGACGGGGGAGCAGATGGCGGCGTTCCAGGAGGCCTTCTCGCTCTTCGACAAGAACGGCGATGGATGCATCAGCTTGGAAGAGCTGGCCACGGTGACTCGCTCCCTCGGCCTCGACCCGACCAACCAGGAGCTCACCGACATGATGCGCGAGGTCGACACGGACGGGAACGGCACCATCGATTTCCAGGAGTTCTTGAGCCTCATTGCCAGGAAGATGCAGGACGGAGACGCCGACGAAGAGCTCAAGGAAGCTTTCGAGGTCCTGGACAAGGATCGAAATGGATTTATCTCCCCTGTTGAGCTGAGGACGGTGATGATCAATCTCGGGGAGAAGATGACCGACGAGGAGGTTGAGCAGATGATCAGGGAGGCGGACACCGATGGCGACGGGCAGGTGAATTACGATGAATTTGCGCTCATTATGAAGAATGCCGAGCGCAAGATAACCGGGTGA

>HvCML60

ATGGCGAACGTGAGCGTCTCGAAGCCGGCCAAGCGCCTGACGGGAAAGAGCAGCTTCAGGCTTGGCCTGCCGCTGCTCTGCGGCCGGTCTGACGTGGCGAGCCCCGGTGCCGCCGCTCCTCGGTCGTCGTCGTCGTCCTCGAGCAGGAGGTCGTCCGGCACCGGGAGCAGCAGGAAGTCCGAGCTGCGCAGGATCTTCCAGCACTTCGACAGGGACAACGACGGCAAGATCTCCGGCGCCGAGCTGAGCGCCTTCTTCGCGTCCATGGGCGACGACCTGACGGTGCCGTCCTCGTCGTCAGGAGAAGGGTACCTGCTGGACTTCGCCGGGTTCGTGGCGCTGATGGAGAGGGGGGAGGGCAGCCAGGAGGAGGACCTGAAGAGGGCGTTCGAGGTGTTCAACGCCGTGGACCAGCCCGCCGGGAGGATCACCGCCAGGGGGCTGCGGCGGGTGCTCGCCCAGCTCGGCGACGACCGGTCAGTCGCGGACTGCGAGGCCATGATACGGGCCTACGACGTCGACGGCGATGGCGGCCTCGACTTCCATGAGTTCCAGAGGATGATGAGCTAG

>HvCML61

ATGAGCATGGTGGTCCTGGACGGGTCGACGGTGCGGTCCTTCGTGGCGGACGAGGCGGCCTTCGCCCGCAGCGTGGACGCGCGCTTCGCGGCGCTCGACGCCAACGGCGACGGCGTGCTCTCCCGCGCCGAGCTCCGCCGCGCGCTCGAGTCCTTCCGCCTCCTCGACGGCGCGGGCTTCGGCTCCGCCGAGCCGGCCCCGCTCCCGGGGGAGGTCGCGGCCCTGTACGACGCCGTCTTCGAGCAGTTCGACGCCGACCACAGCGGCGCCGTCGACCGCGCCGAGTTCCGCGGCGAGATGCGCCGCATCATGCTCGCCGTCGCCGACGGCCTCGGCTGCCAGCCGCTCCAGGTCGCCGTCGACGACGAGGGCGGCAGCTTCCTGCTCGAGGCCGCGGAGCACGAGGCGGCCATGATCGCCGCCAGGGTCCAGGAAGACCGCGACAGGGCCGCGGCCCAGGAGGCCGCCGACGGCAAGTGA

>HvCML62

ATGTGCCAAGCGGCGAGAGCGTACCACAACAATGGCCTGATCGCGTCGGTGTCGTCTCTCCTGATCTCGCTCGTCATGAAGCCCCTGGCCAAGAGCGCCATCCTCACGGGCCGCAGCATCCTCGCCCTCGTCGCCGGCGATGGCGACAGCAGCTCCGTCGTCGCCGTCGCCGACGCTCCACGTGGGCGGCATTGCGATCGCTGCGCCGCGTGCGAAGACGGCGCGCGCCTGTCGGGCTCCGACGCCGCGGCAGTCATGGCGAGCCTCGGCCTCGTCGTTTCACACGGTGACGACGACGACGACGACGACGACGGGATGGTTGAGTGCGGCGGGTGCGAGGCGATGTCGGTGGTGGAGGAGGTGGCGTGGGGGAGCAAGGAGGCCGGGGAGGCGGAGCTGCGGGAGGCGTTCGGGCTGTTCGACCGGGACGGGGACGGGCTGGTGAGCGCGGCGGAGCTGTGGGGCGTGCTGCGGAGGCTCGGGATGGCCGAGGGCACCAGGTACGAGGACTGCGCCAGGATGGTCGCCGCCGCGGCAGCCCGCCACGGCGACGCTACCGGCGGCGTTGACGCCGGGCTCGGGTTCCCCGAGTTCAGGGCCATGATGGAGCACGCGGTTTAA

>HvCML63

ATGCCCAAGCTCTCATCCCTGATATTACGTACATTTCCAGGTAACAGGTTTCTACCACTCTACAAAATAGAGCAATTCGGTGAAATGGGGGGTGTGATTGGAAAAGGAGACACCCCAAGGTACAGTTCAGCAGCTACAAAGTTAGAGCAGAAGATGGTTGATGCCATGCAGCAGAGGGCACAACAAGGGACTTCTCTGAAGTCATTCAACAGTGTCATCATGAAGTTCCCTAAAATTGACGAGAATTTGAGAAACTGCAGGATTATCTTTCAGCAATTTGATGAAGATTCAAATGGTGAAATAGATCAATTAGAACTGAAGCATTGTTTCCAAAAGCTGGATATCTCATTCACAGATGAGGAGATAAAAGATCTATTTGAAGCGTGCGACATATATGAACACATGGGCATGAAGTTCAATGAGTTCATTGTCTTCCTGTGCCTTGTTTATCTTCTCAATGATCCAGCTGTGTCAGAGGCAAGAAAAAGAATGGGATTAGGTAACCTTGAGCCGACTTTTGAGACGTTGGTTGATGCATTTGTGTTCTTGGATAAGAACAAAGATGGGTATGTCAGTAAGAATGAGGTGATAGAAGCAATAAATGAGACCAGTGCAGGAGAACGCTCTTCTGGACGCATAGGCGTGAAAAGATTTGAGGAAATGGATTGGGACAAGAACGGAACGGTGACCTTCAAGGAGTTTCTTTTTGCCTTTACTCGCTGGGTGGGGATCGACGATAACGAAGATGACGATGATGATGAATGA

>HvCML64

ATGGCGCAGTCGTCGTCGGCGGCGAGGAAGCCCTCGGCAGCGCCAACCGTCGTCCTGACACTTGTCCTGGTCCTCGCCTCCGCGGGCCTCCTCTTCCTTCTAGTCCACCTCTCCCCTTCGTCGCCATCCGCGCACCCGCACCCCCACCGCCGCCTCCGCCTGCGCGGGGAAAACCTCAGGCACGGAGGCGCCACCCGGCACCAGATCCCGTTCGACCCCGTCATCGCGGACCTCGAGCGCCGCGTCGACGACCGCGAGTGGGAGCGCCTGGCTGCCGCGGGGCTGCACGCGCCGGGCATGGAGTCTGCCCCCGTCCCAGAGGACCTCGCGGACTACGAGGACGAGTACATCAATGACGCCGCGCGGTTCAACATGACGCTGCGCGTGGCGGCTCTTTTCCCCAAGATCGACGTGGACCCCGCCGACGACGCCGTGACGGGCGCCGAGCTGGCTGCGTGGAATCTCGCGTCCGCGCGGCGGGAGGTGCTGCACCGCACCGCCCGGGAGCTCGACCTGCATGACCGCGACCACGACGGCCGCGTCGCCTTCTCCGAGTACGAGAGGCCCAGCTGGGCTTGGCGGTTCGACGATAATAACTCAAGCAGTGATGGGATGGGATGGTGGAAGGAGGGGCACTTCAATGCTGCGGATATGGACGGCGACGGCTTTCTAAATCTGACGGAGTTTAACGACTTCTTACATCCAGCTGATACTACCAACCCAAAGCTAATACATTGGTTGTGCAAAGAAGAAATCAGGGAAAGAGACAAAGATGCTGACGGGAAGCTCAATTTCCAAGAGTTCTATAAAGGATTATTTTACTCAGTTCGACATTACGATGATGAAACTTCAACAGATGACTCCAATGGCTCTGATGCACCGGCTAGAAAATCGTTTTTACAACTTGATCTGGATAATGATGGGTTTTTGTCAGCAGATGAGCTAAAACCTATCATCGGAAAACTCCATCCAGCAGAAAACTTCTATGCCAAGCAACAAGCTGACTATGTGATATCACAGGCTGATACAAATAAAGATGGACAGCTGAGTTTGAATGAGATGATTGAGAACCCCTATGTGTTTTACAGTGCTTTATTCACAGAAGATGATTATGGGTCTCATGACGAGCTCCGGTAG

>HvCML65

ATGGGTCAAGCTGTCTCCGCCGCTTCTTCCTCCTTCCGCCGCAAATCGCGGGCGCCGTCACCGGTTCCTCAGGCCACCGCCCCTCCAGCCGCGAAGGACCACGACCCGGAGCTCGTCCGCATCTTCCGCCGGTACGACACCGACGGGGACGGCCGCATCTCGGCCGCGGAGATACGCGAGATTTGGGGCTGCACGGACGCGGAGGCGCAGGAGATGGTCGCCGAGGCGGACAGCAACGGGGACGGCTTGATCAGCATCGAGGAGCTCGGGGCCCTGCTCAAGGACGGGGGCTCGGAGGACTTGCCCACGGCGTTCGCGGTGTTTGACGAGAACGGCGACGGGGTGATCACCCCCAAGGAGCTGCGCCGAGCGTTCCGCATGCCGCTGCTCGGCGGGGAGGAGCACACGATTGAGGAGTGCTTCAGGATGGTCGCCGCGTTTGACCAGGACGGCGACGGCGTCCTCTCCTTCGACGAGTTCAAGGCCATGATGGCGCCCAAGAGCGCGTGA

>HvCML66

ATGAAGAAGGTGTTCTCCCGCTTCGAAACGGACGGGGACGACAGGATCTCGCCCTCGGAGCTGGCGGCCGTGTCGCGCGCCATCGGGTCGCCGGCCACCGAGTCGGCAGGGGGCCGGGAGGTGGCGTCCATGATGGACGAGCTCGACACCGACCGCGACGGCTACGTGGACCTCGGCGAGTTCGCCGCCTTCCACGACCACGGCCGCGGGGAGCGCGAGCTGGACGCCGAGCTGCGCGATGCCTTCGACGTCTACGACATCAACGGCGACGGCCGCATCTCCGACGCCGAGCTCAGCAAGGTCGTGTCCCGGATCGGCGAGGGTCTGCTTGTTCTTGGCGTGTTGGCGAGCAGAGCGCATACACCGCGGTGCGCCCCGAGTGGATGTGCTGGGAGGAGGTGA

>HvCML67

ATGGCGTTCATGCGGTACGACTACAGGGCGCTGCCGCAGGAGACGACGGTGGAGGAGTTCAGGGCGTGGCTGGCGCAGTTCGACGCGGACGGGGACGGGCGGATCAGCCGGGAGGAGCTGCGGGAGGCGCTGCGCAGCCTCGACCTGTGGTTCGCATGGTGGAAGGCTAGGGAGGCGCTGCGGGACGCCGACGCCAACCGCAACGGCCTCGTCGACGGCGACGAGATGGCCAGGCTCTACGCCTTCGCCCGCAACAACCTCCACCTCAAGGCCGCCGACCTCGACGTCGATGCATAG

>HvCML68

ATGGGGAACGCGTCGTCGATGCTGACGCAGTACGACATCGAGGAGGTGCAGGAGCACTGCAGCTACCTATTCTCGCAGCAGGAGATCGTGTCGCTGTACGAGCGATTCTGCCAGCTCGACCGCAGCGCCAAGGGGTTCGTCTCCGAGGACGAGTTCCTCTCCATCCCCGAGTTCTCCACCAACCCGCTCTCCCAGAGGTTGCTACGCATGGTTGATGGATTGAACTTCAAGGAATTTGTTTCTTTTCTCTCTACCTTCAGCGCAAGGGCCAGCCTTCAGCAAAAGATTGAGTTGATATTTAAGGTGTATGACGTTGATGGCAAGGGGAAAGTATCCTTCAAGGACCTGGTAGAGGTTTTACGGGATCTAACTGGCTCATCCATGTCAGAGAAACAAAGAGAGCAAGTACTAACAAAGGTGTTGGAAGAAGCCGGGTACACACAGGATTCCACCCTAGCAATAGAAGATTTTGTGACGATCATCGACCACCCCGGCCTGAAGATGGAGGTGGAAGTGCCCATCGACTAA

>HvCML69

ATGCAAATAGAACGGCCGAAAGCTGAAAATATGAGCAGCAGGGTGGATGATTCGGAGCTAAGGAAGGTCTTCCAGATGTTCGACAAGAACGGCGACGGCCAGATCACCAAGAAGGAGCTACGTGAGTCGCTCAAGAACCTAGGGATCTACATCCCGGAGGATGAGATGGACGCCACCATGGCCAAGATTGATACCAACGGCGATGGCTGCGTCGACATCGAGGAGTTTGGCCTACTATATCGCTCCATCCTCGATGAAAGCGAGGGGCCTAACGGTGGCAACATGGGCGATGAGGAGGAAGCCATGAGGGAGGCATTCTGTGTCTTTGATCAGAATGGTGATGGCTACATCACCATTGAGGAGCTGCGGTCCGTGCTGGCAAGCCTCGGCCTCAAGCAGGGACGCACCATCGAGGAATGCCGCCAGATGATCAGCAAGGTCGATGCCAATGGCGATGGTCGCGTCGACTTCAAGGAGTTCAAGCAAATGATGCGTGGTGGTGGCTTTGCTGCCATCGGCAGATCATGA

>HvCML70

ATGGCCGACTACAACCGCTACGGCCACGCCCACGGCCAAGGATACGGCCAGCAGGGCTACGGCTACGCGCCATCGGCCCCTCCCGCGCCCACGCCCTCCTCCTCCTCGTCGTACGGCTACGCTCCCTCGGCCTCCTCGCCGTACGGCTACGGCTACGGCCGAGGAGGCTACCCGCCGCCGCCGATGGGCGGTTTCGGAGGAGCCGTGGCGTTCCCGCCGGGGACGCACCCGGACGTGGAGCGCGCGTTCCGGGCCGTCGACCGCGACCGCAGCGGCAGCATCGACGAGGGGGAGCTGCAGGCCGCGCTCTCCGGCGCGTACCACCGCTTCAGCATCCGCACCGTCCGCCTCCTCATCTTCCTCTTCAGCGACGCCTCCCCGCGCTCCCGGATGGGGCCGGCGGAGTTCGCGACGCTGTGGAACTGCCTCGGGCAATGGCGGGTCGTTTTCGACAGATACGATAGGGACCGCAGCGGTAAGATCGAATCCAACGAGTTGAGAGAAGCTCTTCGCGGCCTCGGATATGCGGTACCACCTGCGGTCATCGATCTTCTCATAGCAAACTACAACAATGGTGTTTCCAACCGGGGCGCCCTAGACTTCGACAACTTTGTGGAGTGCGGAATGGTTGTGAAGGGTCTGACTGAAAAATTTAAGGAGAATGACACGCGCCACACCGGCTCAGCTGCTCTTTCGTATGACGGCTTCTTGTCAATGGTCATCCCCTTCATTGTACCATAG

>HvCML71

ATGGCGTTCATGCGGTACCGTGCGCTGCCGCAGGGGGAGGTGACGGCGGAGGAGTTCTGGGCGTGGCTGGGGCAGTTCGACGCGGACCACGACGGCCGGATCAGCCGGGACGAGCTTCAGCGCGCGCTGCGGAGCCTCAACCTGTGGTTCGCGTCCTGGAAGGCGCGGGAAGGGGTGCGTTCCGCAGACGCCAACCGCGACGGCGCCGTCGGCAGGGAGGAGGCCGGCCGGCTCTTCGCCTACGCGCAGAGGCAGCTCGGCGGCAAGATCACCCAGCTCCGATCCTACTGA

>HvCML72

ATGGCGTTCATGCGGTACCGTGCGCTGCCGCAGGGGGAGGTGACGGCGGAGGAGTTCTGGGCGTGGCTGGGGCAGTTCGACGCGGACCATGACGGCCGGATCAGCCGGGACGAGCTGCAGCGCGCGCTGCGGAGCCTCAATCTGTGGTTCGCGTCGTGGAAGGCGCGGGAAGGGGTGCAGGCCGCGGACGCCAACCGCGACGGCGCCGTCGGCAGGGAGGAGGCCGGCAGGCTCTTCGCCTACGCGCAGAGGCAGCTCGGCGGCAAGATCACCCAGCTCGGATCCTACTGA

>HvCML73

ATGGCGGATGACATGGAGAGGATCTTCAAGAGGTTCGACACGAACGGTGACGGGAAGATCTCGCTGTCCGAGCTGACGGACGCGCTGCGGACGCTGGGGTCGACCTCCGCGGACGAGGTGCAGCGCATGATGGCGGAGATCGACACCGATGGTGACGGCTTCATTGACTTCAGCGAGTTCATCTCCTTCTGCAACGCCAACCCGGGACTCATGAAGGACGTCGCCAAGGCCTTCTGA

>HvCML74

ATGGATACGAGGCAGAGCGTCGCGTCGGTGGTGAAGCCGTCGTTGCCCACCGATGGCGGTGCCCCGGCCACGGCGTCGTTCCGTCTCCGAAACGGCAGCCTCAACTCGGTTCGCCTCCGCCGGGTGTTCGACCTGTTCGACAAGAACGGCGACGGTGAGATCACGGTCGACGAGCTGGCGCAGGCGCTGGATTCACTCGGGCTCGTCGCCGACCGCGAGGGCCTGGCCGCCACCGTGGGCGCATACGTCCCCGAGGGCGCGGCGGGGCTTCGGTTCCAGGACTTCGAGTCCCTCCACCGCGAGCTGGGTGACGCGCTCTTCGGCGCGCTGGATGACGTGCCCGAGGATGGCGAGGCCGGCGCCGGTGGGGACGAGGAGGAAATGAAGGAGGCGTTCAAGGTGTTCGACGTCGACGGCGACGGCTTCATCTCGGCATCCGAGCTGCAAGAGGTGCTCAAGAAGCTGGGCCTCCCCGAGGGCGGCAGCCTCGCCACCGTGCGCCAGATGATTTGCAACGTCGACCGTAACAGCGACGGCCGCGTCGACTTTGGGGAGTTCAAGTGCATGATGAAGGGGATCACGGTGTGGGGCGCGTGA

>HvCML75

ATGGCCGGCAAGGAGCTGAGCGAGGAGCAGGTTGCGTCGATGCGGGAGGCCTTCTCCCTCTTCGACACTGACGGCGACGGCCGCATCGCCCCCTCGGAGCTCGGCGTCCTCATGCGTTCCCTGGGTGGGAACCCGACGCAGGCGCAGCTCCGCGACATCGCCGCCCAGGAGAAGCTCACGGCCCCCTTCGACTTCCCGCGCTTCCTCGACCTCATGCGCGCCCACCTCCGCCCTGAGCCCTTCGACCGCCCGCTTCGCGACGCCTTCCGCGTCCTCGACAAGGACGCATCGGGCACCGTCTCCGTCGCGGACCTCCGCCACGTCCTCACTTCCATCGGCGAGAAGCTCGAGCCGCACGAGTTCGACGAGTGGATCCGCGAGGTCGACGTCGCCGCGGACGGCACCATCCGCTACGACGACTTCATCCGCCGCATCGTCGCCAAGTAG

>HvCML76

ATGGGGGGCGTGTTTGGACGCCACGACGCCGGCAGGCAGAGCTCCCACGGTATGAAGCTAGAGTCGAAGATGGTGGAGTCCATGAAGCAGAGAGCGTCGCATGGAACTTCGGTCAAGTCGTTCAACAGTATTATCATGAAGTTCGCGAAAATCGACAAGGGCTTGAGAAAATGCAAGACTATCTTTCAGCAATTCGATGAAGATTCCAATGGTGAAATTGATAAAGAAGAGCTCAAGAATTGCTTTCAGAAGCTGGAAATCTCATTCACAGAGGAGGAGATAAGTGATCTCTTTGAAGCTTGCGACATAAATGAAGATATGGGCATGAAGTACAATGAGTTCATTGTCTTTCTGTGCCTTGTTTATCTTCTCAATGACCCTGCTGCATCAGAAGCAAAAACAAAGATGGGATTAGGAGATCTTGAGTCAACTTTTGAGACCTTGGTTGATGCATTTGTCTTCTTGGATAAGAACAAGGATGGGTATGTGAGTAAGGATGAGATGATTCAAGCAATAAATGAGAGCATACCAGGGGAACGCTCTGCTGGCCGTATAGCCATGAAAAGATTTGAGGAGATGGATTGGGACAAGAACGGGATGGTTACCTTCAAGGAATTTCTGTTTGCATTCACTCGCTGGGTTGGGATAGGCGAAAACGAGGACGAAGATGAATGA

>HvCML77

ATGGCGACGCCTGGCCGCCGGCCGTCGGCGCAGCAGCAGCCGCAGCCGCTGACGGTGGACTTCGAGGCGCTGAGCTACATCAGCAGGCTCGTGGAGGCTTTCCAGGCGTTCGACTCCGACAACGACGGCCTCGTCACTGCCCCCGAGCTCCGGGGCCTCCTGGCCTCCCTGGGCCTCGACAAGTCGGAGGCGGAGGCGCGGGACATGCTGGCGCGCGCCGACGCCGACCGTGACGGCCGGCTCTGCGTGGAGGAGTTCCTGGACGTGATGAACGCCGGCGAGCTCGGCCTGGGCGCGCTCGGCGAGCTGCTGCAGGCCGCCGTCCCGGCGCTGGAGTCCTTCGCGGGGCCCGACGGCGCGCTGGGCGCCGACGAGCTCGCCAGGGTGCTGGGGCTCATGGGCACCGCCAGCGCCGAGGACTGCGCGGAGATCATCGCGTGCATGGACGGGGACGGCGACGGCGCCATCAGCGTCGAGGAGTTCAAGCTCATGGCTGACCTGCTCTAG

>HvCML78

ATGGCGAGCTATTTCAGAGGGCAGCGGCTGGAGCCTTCTTCGGATGCCCCCCGGCCCGGTTACCGGAGGGACAGGGGCGGGCGCAAGCGGCTGACGGCGCAGAAGAGGAAGGAGATCAAGGAGGCCTTCGACCTCTTCGACACCGATGGCTCCGGCACCATTGATCCAAGGGAGCTCAACGTTGCAATGAGAGCTCTGGGGTTTGAGATGACGCCGGAGCAGATCCAGCAGATGATCGCGGAGGTGGACAAGGACGGCAGCGGGACGATCGACCTGGACGAGTTCATACACATGATGACGGACAAGATGGGCGAGAGGGACGCCAGGGACGAGCTCCACAAGGCCTTCCGGATCATCGACCAGGACGCCAACGGGAAGATATCGGACATGGACATCCAGCGGCTGGCCATCGAGGCCGGCGAGCACTTCACGCTCGACGAGGTCAGGGAGATGATCGAGGCCGCCGACGAAAACGGTGACGGCGAGGTCGACATGGAGGAGTTTATGAAGATGATGAAGCGGACGGGCTTCGGTGCCGGATTCTAG

>HvCML79

ATGGTGGTGCCGTCGGTGTTCGCGGCCTTCGACGAGGATGGCGACGGCAAGGTGTCCGTGTCCGAGCTGCGGCGCTGCGTGGAGGCGACGCTGGGCGAGGACGTGTCCGAGGAGGAGGCTGCGTCGGTCCTTGCGGCGGCGGACGCCGACGGCGACGGGCTGCTAAACCAAGAAGAGTTCTCAAGGCTGGCAGCCGGCGGTGCCCAGGAAGAGGACGATGCCGACGTGAAGCGAAGGTGCCTGAGGGAGGCGTTCGGGATGTACGCGTCGTCGTCTGCGGAAGACACGGCGACGATGATTACGCCGGCGAGCCTGAGGCGGACGCTGAGCAGGCTGGGGTCGCACGAGCTGGGCGTGGAGGAGTGCCGGGCGATGATCTGTAGGTTCGACCTCGACGGCGACGGCAAGCTCTCGTTCGACGAGTTCAGGGTCATGATGATGACCTGA

>HvCML80

ATGGTGGTGCCGTCGGTGTTCGCGGCCTTCGACGAGGACGGCGACGGCAAGGTATCCGTGTCCGAGCTCCGGCGCTGCATGGAGGCGACGCTGGGCGCGGACGTGTCCGACGAGGAGGCGGCGGTGATCCTCGCGTCGGTGGACGCCGACGGCGACGGGCTGCTAAACCAAGAAGAGTTCTCAAGGCTGGCCGCCGGCGCCCATGAAGAGGACGATGCCGACGTGAAGCTAAGGTGCCTGAGGGAGGCGTTCGGGATGTACGCGTCGTCTTCCGCGGAAGACACGGCGACGATGATTACGCCGGCGAGCTTGAGGCGGACGCTGAGCAGGCTAGGGTCGCACGAGCTGGGCGTGGAGGAGTGCCGGGCAATGATCTGCAGATTTGACCTCGACGGCGACGGCAAGCTCTCGTTCGATGAGTTCCGGGTCATGATGATGGCCTGA

Amino acid sequences

>HvCaM1

MADQLTDEQIAEFKEAFSLFDKDGDGCITTKELGTVMRSLGQNPTEAELQDMINEVDADGNGTIDFPEFLNLMARKMKDTDSEEELKEAFRVFDKDQNGFISAAELRHVMTNLGEKLTDEEVDEMIREADVDGDGQINYEEFVKVMMAK

>HvCaM2

MADQLTDDQIAEFKEAFSLFDKDGDGCITTKELGTVMRSLGQNPTEAELQDMINEVDADGNGTIDFPEFLNLMARKMKDTDSEEELKEAFRVFDKDQNGFISAAELRHVMTNLGEKLTDEEVDEMIREADVDGDGQINYEEFVKVMMAK

>HvCaM3

MADQLTDDQIAEFKEAFSLFDKDGDGCITTKELGTVMRSLGQNPTEAELQDMINEVDADGNGTIDFPEFLNLMARKMKDTDSEEELKEAFRVFDKDQNGFISAAELRHVMTNLGEKLTDEEVDEMIREADVDGDGQINYEEFVKVMMAK

>HvCaM4

MADQLTDDQIAEFKEAFSLFDKDGDGCITTKELGTVMRSLGQNPTEAELQDMINEVDADGNGTIDFPEFLNLMARKMKDTDSEEELKEAFRVFDKDQNGFISAAELRHVMTNLGEKLTDEEVDEMVREADVDGDGQINYDEFVKVMMAK

>HvCaM5

MADQLTDDQIAEFKEAFSLFDKDGDGCITTKELGTVMRSLGQNPTEAELQDMINEVDADGNGTIDFPEFLNLMARKMKDTDSEEELKEAFRVFDKDQNGFISAAELRHVMTNLGEKLTDEEVDEMIREADVDGDGQINYEEFVKVMMAK

>HvCML1

MAGSCRSVHFYFWASDSTSSGAPLPPRSTEIAVTKTDSIPHSESNPTTTYTTYNKTLNSTTQGSTQASRAVSEEEEEEEEGGGGMGGAASRLAAPIKHRRGEKELDNRVAEALRERAKARTRTFRSVNSITMRLPRFKDGLKDIRDVFDHYDADSNGTIDNEELRSCLSKLEVRMSERESDDVHRYCDVDSRKGIQFQEFVVLICLMYLLFGPNVTRRVSEFESAKLNYIFDELIDAFLFFNRDGDGKVTRKDVTQRMNEECDRERTPAHITTQLFKEMDLNKNGKVNLKEFLFSMIRWAGLEPEEDDESNDISP

>HvCML2

MANGVGSAKPEICAGLGMPMAELEQVFRRYDANGDGKISADEMASVLCALGAPPGPGEVQSMMEEMDADRDGFVDLHEFAAFHCGPCKAGAGADAKEQEDATEAELKEAFRMYDADRNGLISARELHRVLRQLGDKCSVSDCSRMIRSVDADGDGSVNFEEFKKMMGGGGSNHSRLSFKSFDVLGLDVLLPTPMTGHVLLQPHSFGSNFCIRSRIKTIQLSKITASMR

>HvCML3

MSEAPGTTAAAGDDPDATANANQQQQQQQQQAQPEAQDDADQLSELRQIFRSFDRNKDGSLTQLELGSLLRSLGLKPSTDELDALIHRADTNSNGLVEFSEFVALVAPSLLDDRSPYSEDQLRRLFEIFDRDGNGFITAAELAHSMAKLGHALTAKELTGMIEEADTDGDGRIDFREFSRAITAAAFDNVFS

>HvCML4

MGKMRSLFSRSRSGNGGSRRSTSSSRSSAPPSPARGASREDEMERVFRKFDANGDGRISRAELAALFESVGHAVTDDEVARMMEEADADGDGYISLAEFAAINAAPDAAVEEDLRHAFRVFDADGNGVISPAELARVLRGLGEAATVAQCRRMIEGVDRNGDGLVSFDEFKLMMANGAGFAIAQGNVRA

>HvCML5

MAAKLTREQADECKEVFDLFDGDEDGRIAAGELVTALRSLGQNVDEAEARGFLEDAGAGAGAAAVDLPTFLAVAARKANAGVSAKRLVECLDAFDDDGSGVIPAEQLRQVMLTHGDRLTEEEADELVRKADPRGEGRVQCKELVKVLMNNK

>HvCML6

MVHTATAECFSSVFASFERDADGRISAAELWLCMKAALGEDVSAQDAEALVASADADGDQLLDEQEFLRLVARPETEEEEWCRGLREAFAMYEVKGEGCITRSSLMRMLARLGSEQGIEECRAMIRRFDLNEDRVVCFDEFKVMMDA

>HvCML7

MSTHPTNLASYRSPRSTRTTVAARDDPDATANANQQQQAQPEAQDDVDQLSELRQIFRSFDRNKDGSLTQLELGSLLRSLGLKPCTDELDALIHRADTNSEFVALVAPSLLDDRSPYSEDQLCRLFEIFDRDGTGFIIAAELAHSMAKLGHALTAKELTGMIEEADTDGDGRIDFREFSRAITAAAFDNVFS

>HvCML8

MDKSPATAGCHLEPLFLEPLGILILFILTWFISEVQRLLPSSCQSCSCPVSTTTSPPVLAETSKAPNKSEYVEMNAKQSDAEIAMRKMGFDFDQEKSCEHISTLFDDDEPSFQEVKMAFLVFDENNDGYIDALDLRRVLHNLGLGDRVGVSESEQMIARYDMNNDRRIDLMEFTKVLEDSFC

>HvCML9

MHPPTRSPPTPTTRLPPCRQCPVISPLLSSINIYQSRRLHSPPRPGHHPKPILHPPPPPPPIPSHRPSTMTKASPALRGSQLKQLRSLFDRFDMDGDGSLTQLELAALLRSLGLRPTGDESRALLLAIDADGSGTVEFDELARAIAPVLTAHAPRLVDQAQLLEVFQAFDRDGNGYISAAELARSMAKLGQPLTFDELRTMMRDADADGDGVISFGEFAAVMARSALDFLGVPAA

>HvCML10

MWVKMLMDQNMLIALVSSLLMLILGPLIKDIILVSKKIWSFLCTLTKYLVHNDTLAVDSVVLDDNISPPAQLACGGGLTSGDIEIVTARLGLTRFSYQGCEGLGVVEELMDGKQASQDELEEAFCIFDRDEDGFICTGELWNVMRRLGWKEGAMYEDCVRMIRAFDEDGDGKISFLEFRRMMENAV

>HvCML11

MWAVVIMGQNILIALVSSLLTLILGPLIIDVILVSKKIWSFLHTFTKYLVHDDTLVIDSVVLDDSPMLPAQLVGGGGLTSVDIEIVTTRLGLGGWSYQGCEGIGVVDELIDGKQASEDELEATFYIFDRNEDGFICAGELWNVMRRLGWKEGAMYEDCVRMIRAFDEDGDGKINFLEFRRMMENVV

>HvCML12

MAGEHQQSQGAAKPLSKGAPSPSFRLRNGSLNAVRLRRVFDMFDRNGDGEITVDELAQALDALGLEADRASLAATVGAHVPPGASGLRFEDFEGLHRALGDALFGALADDGEDGGAGGEDEEEMREAFKVFDVDGDGFISASELQEVLKKLGMPEASSLANVREMICNVDRDSDGRVDFGEFKIMMQGINV

>HvCML13

MADAIAPRPVLPRALSFREPLLLIPYFLGFLGTVASALFYNYASFLRSFARSIVVPSPAAACAKCTYATSSVPCCEDADADAEEMRKEEVEAIMARIGLGVTGAGEGLRASMGHNEVSRLFDAEEPSFAEVRRAFAVFDGDADGFIGAADLQGALTRLGLPDVDSAACEATISSSCGSTDGRMNLFQFVRFLEDGLQESGWMDCATN

>HvCML14

MADATAPRPLLRRMLSFREPLLLIPHLVFFLGTVASAFFHSYASFLQSFARSVVVPSPAACAKCAYAASSSAVCCDDAAVAEERGEGEELRKEEVEAIMARIGLGVAGAGEGLRASMGRNEVSRLFDAEEPSFAEVRRAFAVFDGDADGFIGAADLQGALARLGFPEVDAAACRAMISSSCGSTDGRMNLFQFVRFLEDGLC

>HvCML15

MAGRRWLLWPGGLTVEEFKEWLKQFDVDGDGRISRAELRKAIRSRGVWFATLRAGRAIRHADRDKSGYVEDAEIENLVAFAQKDLGMKISAW

>HvCML16

MPIRGVPCAWTVEEFKSWLKQFDVDRDGKISKAELRQAIRRRGCWFATARAGRAVRRADRDHNGYVDDAELENLVAFAREHLGMNISAR

>HvCML17

MAIRGIPSAREMTMEEFKEWLKQFDVDGDGRISRAELREAIRRRGGWFTTLRAGRAVRRADRDNSGFIDDAEVENLVAFAKKDLGMRISAW

>HvCML18

MSTMKGQTRRERPRTRPHGLTQQKRQEIKEAFDLFDTDNSGTIDAKELNVAMRALGFEMTEEQINQMIADVDKDGSGSIDYEEFEHMMTAKIGERDTKEELTKAFRIIDQDKNGKISNVDIQRIAKELGENFTLQEIQEMVQEADQNGDGEIDFGEFARMMKKTSYGGY

>HvCML19

MLEQIDSNTDGFVDFEEFVAATLHMHQLVEHDAEKWKSLSQAAFDKFDVDGDGYITSNELRMNTGLKGSIDPLLEEADIDKDGKISLDEFRKLLKTASMRSCNPTPRSVSK

>HvCML20

MVQGKGFAPPPPNLSPPNQLNSSDLQSLHSKATPPPSMPPSGLLSYIPTSLSSILPARGCGAAPSPSPPPPASPAPSKMSPSVKAADRAELARVFELFDRNGDGRITREELEDSLGKLGIPVPGDELAAMIARIDADGDGCVDVEEFGELYRTIMSTGSGGGQKGSSDAEAEEEDEDEDMREAFRVFDANGDGFITVDELSAVLASLGLKQGRSAEECRRMIGQVDRDGDGRVDFHEFRQMMRGGGLAALA

>HvCML21

MAGEEQPLTEYEKERLARIRENEARLEALGIRRLAASPLLNQPSSAAAAAAAGAKRKQKKRSDDADEEYLPSDGGGGEESSSASDQDTEEDFKPSSRSNQKGKAKKKLNLGSPSKSTFREEDAPLTDFMDDDAALQQAIALSLAEPSKSSVTTAEPSKSSVTTTTAAEPSKSSVTTTTAAEPSKSSVTTAEPSKSSVTTTTTAETSSRGAKGRKGTPCKNDNTTPVKDSAKNRKTKKQVRSRIQLSEDDVVAIFFSFDEAGKGYIAPWDLEKMANVNDFIWTDFELSNMINFFDNDKDGKISLEEFRAIVSRCNMLQEPGE

>HvCML22

MACQSGIHQSAAGSSSRLLCSHPADSLHLNNKNSSTPPTKTLLQGCNADVDNNGTIDYIEFIAATLHLNKLEREEHLVAAFSYFDKDGSGYITVDELRQACLEHNMPDAFLDDVIKEADQDNDKCGLDSIYLLFSRYGISWLPINPLLSS

>HvCML23

MQSGQQQARRKNAQVLDGSDIRELVENKEAFAKFVENKFRHLDADGDGRLSLKELQPAVADIGAAIGLPARGSSAQADHIYSEVLNEFTHGKQDSVSKPEFQHVLSDILLGMAAGLKRDPIMILRVDGEDLNEFVESAAYEAEAVAIFSQIESGNSSLRQCLPAALRQLTVDHGMPPASDSLVMEKIIVPALQELPADQLDQPASQEVFFQEFKKYLGMIARRLQECPIIVAHTENTFDGAGIRKILSNKFEFDKLLDSVWGDVPKEHKDRTSKKYLRVAFDKMAASVNLPPYGAVNQVDAMVNDAFKMANADDGKAVDEAEFKKLLTEILGAVMLQLDGNAIAVSTNTVLHEPMSTSSTLLSPSPSSPTVSSPSE

>HvCML24

MGQVWASLQEKLQGRHWKERQVRKITDKVFDRLTEDTQRREKEALQFEEVYIAVLCVYNDINKYLPGPHYDPPSKERLKALMNEFDIDMNGLLDREEFAEFIRKLTAESLCAISLKLIITLVAAPALALATKRATEGVPGVGKVVHKVPNAIYASAITLAAVLIQRSAEGVE

>HvCML25

MSSGGQRQQQAKKPSPAAAGADEIEIKKVFSRFDTDGDGRISPSELAAVSRAIAPPATESAGGREVASMMDELDTDRDGYVDLGEFAAFHGRGRGERELDAELRDAFDVYDINGDGRISVAELSKVLSRIGEGCTTQDCEKMIASVDVDGDGCVGFEEFKKMMAGDGAARPLDGGVPDDDGKAKTE

>HvCML26

MTPPPAVPPGRKSPAAVLFLCLVTTSLLMFILLASYTPRLEPHGRSPHRRLKLHPKNSAAVASSYGAGAVHESGGNRHAAPFDPAIAELERRLEDKEWEREHYRILHGDAEKGDHMKEWEDFLKEEEDFINDDDRFNISDRIRALFPKIDLSPEDGFVSLDELIRWNLDQARADQLHRSAREMELYDKNGNGIVSFTAFQTLRQQSHGDGNSLGFPWWKEEHFNASDVNGDGFLNKTEFHDFLNPSDSENPKIINLLCRQELRQRDKDGDGKLNFEEYFHGLHDHIHGYDDENAAISHIGNMTIAKERFSKLDKDNDGFISGHELEPVLDKLHLSERYYARQQATHAISEADKDHDGRLTLEEMIENPYAFYGSVYFSDDEDYFHEEFR

>HvCML27

MKRPRHQKLKTRSRRKGWSDLVATPRNMQSHRPAPEDHVREESRSTRAQPTTRPHAAATRTTATRARRSAFMSVPSDAGQETPPSATEGGRARLQDEQLGQLRELFLRFDLDGDGSLTKLEIAALLRSLGLRPAAGDEIHTLIASMDADGNGTVEFDELASSLSQLLLGPGRPAVAVDHEQLAEAFRAFDRDGNGYISAAELARSMAQMGHPICYAELTDMMREADTDGDGSISFEEFTAIMAKSAVEFLGLAAL

>HvCML28

MASMEVAKPGSSKRMSPKGSFKLSLLACGQCKATTVSPPDSPTGAGARSLSSSASSSAGTSRDRQAELREIFRHFDRDMDGRISGRELGEFFASMGDGGAKAALELDAAGGGDLMLGFEDFVRIVERKGGEEEEREDLRRAFEAFEAVKGSGRITPRGLQRVLSQLGDDPSVAECEAMIRAYDDDGDGELDFHDFHRMMNHD

>HvCML29

MATIGEFRRVFSAFDQDGDGKISAAELQLCMKAALGCDMSVEEVQSLMASADTDGDGLLDKEEFLRLVLETEAGKEEEGDRCREAFGMYEMEGRGCITPLSLQLMMSKLGLHLAVDECQAMIRRFDLNGDGVLTFDEFKTMMMMG

>HvCML30

MADQLSEEQIGEFKEAFSLFDKDGDGSITTKELGTVMRSLGQNPTEAELQDMINEVDADGNGTIDFPEFLNLMARKMKDTDSEEELKEAFRVFDKDQNGFISAAELRQVMTNLGEKLSEEEVEEMVREADVDGDGQINYDEFVKVMMAKRRDKRVEERRAPPARKSAAGASPSGAKSGNKCIIL

>HvCML31

MKLSMQSLARKLSIPSPKRGKKQQQQEESGKRGISRSEAPSFASASSSSTASSSASEDAPARASTPRSVLPAEISRRELEAVLRRLGHEEPSDDELDAVAAIAAAGEAGPEDELMEAFNVFDADGDGRITAEELRGVMVAILGGEADGCSLDDCRRMIGGVDADGDGFVGFQDFARMMMVSTTAAAGPRFL

>HvCML32

MGKIKMPSLFRRRSSSKSRSSSPPPPQQQEEEGDRAASGAGSPARTAEEEMERVFRKFDANGDGRISRPELAALFESLGHAATDDELSRMMAEADADGDGFISLAEFAALNATAAGDDEEDLRLAFKVFDADGSGAISAAELARVLHGLGEKATVQQCRRMIEGVDKNGDGLISFDEFKVMMASGFAAKMA

>HvCML33

MVAAKSAELRALFLSLDRDADGRISPAELQGCMRATLGEDVPAEEAEALVASVDADGDGLLSESEFLELAQQADAGEEDGERRIWALREAFGMYEMEGLGCITPASLARMLGRLGAERGAGECRAMICRFDLNGDGMLSFDEFKIMMS

>HvCML34

MVASKSGELSTLFASLDQDADGRISATELRLCMRATLGEDVPAEEAEALVASADADGDGLLSESEFLELAQQAAWAGDAGEEDDERRIQALKKAFGMYEMEGQGCITPASLGRMLGRLGAERGAGECRAMICRFDLDGDGVLSFDEFKIMMS

>HvCML35

MVVSKSGDLTALFLSLDRDADGLISAAELLGCMRATLGEDVPAEEAEELVASVDADGDGLLSESEFLELAQQAAWGGDAGEEDDEHRIRALREAFGMYEMEGQGCITPASLRRMLGRLGAERGSGECRAMICRFDLDGDGVLSFDEFKIMMS

>HvCML36

MVHAATAERFSSLFASFDRDADGRISAVELRLCMKAALGEEVSAEDAEALVASADADGDRLLDEQEFLRLVAPPETEEEERCRGLREAFAMYEVKGEGCITPSSLMRMLARLGSEQGIEECRAMIHRFDLNGDGVVCFDEFKVMMDA

>HvCML37

MAGYPPPPGSGYPYGAAGGYGAPPPSGQKPPKEGKTSSSSGPDPYHGAPPPQQPYGGGGGGGYGAPPSYGQKPPKEGKTSYSSGSDPYHGAPPPQQPYGGGGGGGYGQQPYGAQPPSSAAPYGGPPAAQPYAGGGAGGYGSPFAALVPSTFPPGTDPNVVACFQAADRDGSGMIDDKELQSALSGYSSQSFSLRTVHLLMYLFTNSNVRKIGPKEFTSVFYSLQNWRGIFERFDRDRSGRIDAAELRDALLDLGYSVSPTVLDLLVSKFDKTGGKNKAVEYDNFIECCLTVKGLTEKFKEKDTAYSGSATFGYEAFMLTVLPFLIA

>HvCML38

MDELSKEQIDEFRAAFSLFDKDGDGTITAKELGTVMRSLGQRPSEEELREMIAEVDADGNGVVDFSEFLVLLDRKMRGADAEDELREAFRVFDQDQNGFISLDEFRHVMDNLGERLSDEELKEMLREADLDGDGQINYSEFARVMMAKMDSTGSKPLVPLLFKNGVRVCRYLLAFRIMEIGRSIKRRRRKSQQNSREMVGEKTGGDADPPPEQGDKDKRGSDSRCIPSCTIL

>HvCML39

MDTAPAPAPAPTKPSLSKKPSPSFRLRNGSLNALRLRRVFDLFDRNGDGEITLDEMAAALDTLGLGADRASLEATVGAYIPAGAAGLGFEDFEGLHRALGDALFGPIAEEEPGKEGEAEDEDMKEAFRVFDENGDGFISAAELQAVLKKLGLAEARNLAAVQEMICNVDRDRDGQVDFGEFKCMMQGITVWGA

>HvCML40

MCPGGRYAGLDLPAGAGAGDLRPAFDVLDADHDGRISRDDLKSFYANAGATDERFDDDDIEAMIAAADADLDGFVQYDEFEGLLGRAAKAGTDGGCRSAMEDAFRLMDRDGDGKVGFEDLKAYLGWAGMPVADDEIRAMISMAGDGDGGVGLEALARILAVDFGAIV

>HvCML41

MERGWFGWRKAKQGGGKEEEGRAKVVVDGSGIRQLVEDREAFGMFAETKFRQLDADGDGRLSVRELQPAVADIGASLGLPAQGSSPNADHIYSEAMSEFTHGHGNQEGVSRAEFQEVLSDILLGMAAGLKKDPIVILRIDGEDLRDFLSSPRYEPVAAAIFSQVGSEDAPLRQCLLAAVQQLGVDHGMPPAADAWVVENVVEPALQQLPADELERRASRDVFLEQLKKLLAGVAEQLQERHVIVAHTENTFDGSGVRRLLGNKFELDKLLDSVWREVPAEHRKKAPKDKEHLRVALDKMADAASLPPHGAVDRVDAVVDEALKVADAGDGKAVEEAEFKKLLTDVLGAVMLRLSGEPIFFSTSTVVHESMPGSSALLPSPAVASPPSE

>HvCML42

MENAVVLREWFDRVDAAGTGNVTAPQLQSALAVGNLDFPLSVVQQMIRMYDFDRNGTMSFQEFLALNKFLHKVQGVFSTLERGRGFLSLEDVYEALIKLGFSLDSPAFYTVCESFDRSKKGMVRLDEFISICIFVQSARNLFSSFDTTKQGKVTFDFNQFVYCTANCRI

>HvCML43

MAGAGATAGISSEQMSEFREAFAFFDKDGDGCITAEELSTVIRSLGQTPTPEELRDMVRDVDADGNGTIEFAEFLALMSRKADADADAADPEEELREAFRVFDKDHDGHISKAELRHVMISLGEKLTDDEVDGMIQEADLDGDGLVNFDEFVRMMMLSDADQHQH

>HvCML44

MAAGAAAAISSEQMSEFREAFAFFDKDGDGCITAEELSTVIRSLGQTPTPEELRDMVRDVDADGNGTIEFAEFLALMSRKADADADASDPEEELREAFRVFDKDRDGHISKAELRHVMISLGEKLTDEEVEEMIQEADLDGDGLVNFDEFVRMMMLSESDQQQH

>HvCML45

MKKVFSRFETDGDGRISPSELAAVSRAIAPPATDSAGGREVASMMDELDTDRDGYVDLGEFAAFHSHGRGERELDAELRDAFDSAYTAVRLEWMWWEEVRRRERGELPARRFEALARSRRAASLALSNRKEIATSHLGAVNFLQEVTYDALTQEIEEIFLFSEETCAQYSWVLLYLKGYHSLQSYRIAHVLWNQGRKVLALALQSRISEVFAVDIHPAAKIGEGILLDHGTGLVIGETTVVGNWVSLMQVVVWFSTGYITIKDMVVTGMPIKIVGLAALKVLLFSEVMCACTYDALTQEIEEIFLFSEVNLNCSPILLYMSRSPNKRK

>HvCML46

MKFFGSSTSKKENKGKKRSKRSGNSGSFASTASSSASDEQSVTTPSSVPPSSSGTAAPSGCGTTKKPAMVAVTRLELEVALRTVVSTEEELAVMLAEAEAGLALDGVEDGEAADEGELRDTFAVFDADGDGRISAEELRAVLATLGDERCSVEDCRRMIGGVDSDGDGFVCFDEFTRMMMLAL

>HvCML47

MACASRVIILPPSPVSSHKNWCQPSFTVRKPPSPPRRDSPHPIPQAPPPKLDRPTMSGYPYGGAGGGGGYGAPPPYGSSPAPSAPPYGDKPPKEAKASSPYYASPPQAYGAGGAGGGGGGYGAPPSSQPQSYGGGYGAPPPSQPQSYGGGGYGAPPSSQPQSYGGGGYGAPPSAQPYGAPPPSSASYGAPPPAAAPYGAAGGYGSPFAALVPSAFPPGTDPNVVACFQAADRDGSGTIDDKELQSALSGYNQSFSIRTVHLLMYLFTNTNVRRIGPKEFTSVFYSLQNWRSIFERFDRDRSGKIDASELRDALLSLGYSVSPTVLDLLVSKFDKTGGMSKAVEYDNFIECCLTVKGLTEKFKEKDTAYSGSATFSYEAFMLTVLPFIIA

>HvCML48

MDELSKEQIQEFREAFRLFDKDGDGTITTKELGTVMRSLGQHPTEEELKDMVEEVDADGSGSIDFNEFLGLVARQMRGDADAEEELHEAFRVFDKDNNGFISLDELRTVMKNLGEKLSEDELNEMLQEADADGDGQINYKEFAKVMMAKRRANAEEHGGGDHGGSDHAHSGGGGCPCTIL

>HvCML49

MVADAVVQASSVFAAFDKDGDGKVSAAELRGSMTAALGEEVSEEEAAAILATVDADGDGLLDQEEFSRLGLGATGDDAGAAGADDEEEVRRRCLREAFAMYATEGGDERARITPASLRRMLGKLLGSEKMGLEECRAMICRFDLNGDGVLSFDEFRVMMMANL

>HvCML50

MDMEMAASSSAPGASYFSFNFSVAQAVVTISINVVVVWLSALVKSSSSSSSSSASSRSPAPAPEPEPAQPAAASGASEVDLDVVLGVMGAAGAASVGFEEAAALFEEEEATVEEAAAAFRVFDCNGDGFVDAGELGSVLRSLGFTAGVAAAECQRMIDAYDENKDGRMDFQEFLSFMERSSS

>HvCML51

MVHAATAECFSSVFASFDRDADGRISAAKLRLCMKATLGEDVSAEDAEALVASADADGYRLLDEQEFLRLVARPETEEEEGRCRGLREAFAMYEVKGEGCITSSSLMRMLARLGSEQGIEECRAMIRMFDLNEDKVVCFDEFKVMMDV

>HvCML52

MSHLSILTFKYNLAKLRFKPARPTGRLLSSRDRQQSDLMMYKPDDEEMAKVFDKIAGEPGLISRSDLQALLQRFDKADAVGEARRMVCAADSNKDGYMDLEEFMEVHKNGVLLGDIRRAFFVFDRDRDGRITAEEVMDVLRKLGDSCSLEDCRKMVKEIDRNHDGYVDMDDFMAMMTRPRKRM

>HvCML53

MSLLHCFLKPSRMSLQRCVVGDDMTAAEFKDWIRQFDADQDGRISRAELRRAMRALRVRFTRRRSRSGISYADADGDGYIDDSEIDGLVEFARTNLGLRIVAC

>HvCML54

MAIRNVTAATRSLDGDMTVDEFKEWLRRFDVDRDGRISRDELRCAMRTIRTRFSGYKSKRGIEYADTDGDGYVDDGEVDGLIEYAQRSLGLRIVAY

>HvCML55

MSVEILDGLTVQSFVEDEGAFNASVDGRFAALDANHDGLLSYTEMAGELMSLRILEKHFGVDEAGADGPEELAGLYRGLFAHFDRDGDGAVDREEFRAEMKEVMLAVASGLGFLPVQMVVEEGSFLKVAVDRELARAA

>HvCML56

MSVEILDGKTVQSFVEDEDAFNSSVDGRFAALDTNHDGLLSYSEMAQELMSLRVLEKHFGVDESAMSHGELVELYRGLFARFDRDGNGTVELEEFRAEMKEVMLAVANGLGFLPVQMVVEEGSFLKVAVDRELGKAA

>HvCML57

MGNASSMLTQYDIEEVQEHCSYLFSQQEIVSLYERFCQLDRSAKGFVAEDEFLSIPEYSTNPLSQRLLRMVDGLNFKEFVSFLSTFSTRASFQQKIELMFKVYDIDGKGKVSFKDLVEVLRDLTGSSMSEQQREQVLTKVLEEAGYTRDSTLSLEDFVTIIDHPGLKMEVEVPID

>HvCML58

MSTAPPQPNQQQQRHRRLRVRRVFDLFDNDGDGVITAGELSGALGRLGLALGAQADVLIAEYVAPGMPGLRFADFEALHAELAGGGEEDEEAEMREAFAVFDENGDGYISAAELQAVLARMGVPEAACMARVRDMIAAHDQDSDGRVDFHEFKAMMAAA

>HvCML59

MEEALTGEQMAAFQEAFSLFDKNGDGCISLEELATVTRSLGLDPTNQELTDMMREVDTDGNGTIDFQEFLSLIARKMQDGDADEELKEAFEVLDKDRNGFISPVELRTVMINLGEKMTDEEVEQMIREADTDGDGQVNYDEFALIMKNAERKITG

>HvCML60

MANVSVSKPAKRLTGKSSFRLGLPLLCGRSDVASPGAAAPRSSSSSSSRRSSGTGSSRKSELRRIFQHFDRDNDGKISGAELSAFFASMGDDLTVPSSSSGEGYLLDFAGFVALMERGEGSQEEDLKRAFEVFNAVDQPAGRITARGLRRVLAQLGDDRSVADCEAMIRAYDVDGDGGLDFHEFQRMMS

>HvCML61

MSMVVLDGSTVRSFVADEAAFARSVDARFAALDANGDGVLSRAELRRALESFRLLDGAGFGSAEPAPLPGEVAALYDAVFEQFDADHSGAVDRAEFRGEMRRIMLAVADGLGCQPLQVAVDDEGGSFLLEAAEHEAAMIAARVQEDRDRAAAQEAADGK

>HvCML62

MCQAARAYHNNGLIASVSSLLISLVMKPLAKSAILTGRSILALVAGDGDSSSVVAVADAPRGRHCDRCAACEDGARLSGSDAAAVMASLGLVVSHGDDDDDDDDGMVECGGCEAMSVVEEVAWGSKEAGEAELREAFGLFDRDGDGLVSAAELWGVLRRLGMAEGTRYEDCARMVAAAAARHGDATGGVDAGLGFPEFRAMMEHAV

>HvCML63

MPKLSSLILRTFPGNRFLPLYKIEQFGEMGGVIGKGDTPRYSSAATKLEQKMVDAMQQRAQQGTSLKSFNSVIMKFPKIDENLRNCRIIFQQFDEDSNGEIDQLELKHCFQKLDISFTDEEIKDLFEACDIYEHMGMKFNEFIVFLCLVYLLNDPAVSEARKRMGLGNLEPTFETLVDAFVFLDKNKDGYVSKNEVIEAINETSAGERSSGRIGVKRFEEMDWDKNGTVTFKEFLFAFTRWVGIDDNEDDDDDE

>HvCML64

MAQSSSAARKPSAAPTVVLTLVLVLASAGLLFLLVHLSPSSPSAHPHPHRRLRLRGENLRHGGATRHQIPFDPVIADLERRVDDREWERLAAAGLHAPGMESAPVPEDLADYEDEYINDAARFNMTLRVAALFPKIDVDPADDAVTGAELAAWNLASARREVLHRTARELDLHDRDHDGRVAFSEYERPSWAWRFDDNNSSSDGMGWWKEGHFNAADMDGDGFLNLTEFNDFLHPADTTNPKLIHWLCKEEIRERDKDADGKLNFQEFYKGLFYSVRHYDDETSTDDSNGSDAPARKSFLQLDLDNDGFLSADELKPIIGKLHPAENFYAKQQADYVISQADTNKDGQLSLNEMIENPYVFYSALFTEDDYGSHDELR

>HvCML65

MGQAVSAASSSFRRKSRAPSPVPQATAPPAAKDHDPELVRIFRRYDTDGDGRISAAEIREIWGCTDAEAQEMVAEADSNGDGLISIEELGALLKDGGSEDLPTAFAVFDENGDGVITPKELRRAFRMPLLGGEEHTIEECFRMVAAFDQDGDGVLSFDEFKAMMAPKSA

>HvCML66

MKKVFSRFETDGDDRISPSELAAVSRAIGSPATESAGGREVASMMDELDTDRDGYVDLGEFAAFHDHGRGERELDAELRDAFDVYDINGDGRISDAELSKVVSRIGEGLLVLGVLASRAHTPRCAPSGCAGRR

>HvCML67

MAFMRYDYRALPQETTVEEFRAWLAQFDADGDGRISREELREALRSLDLWFAWWKAREALRDADANRNGLVDGDEMARLYAFARNNLHLKAADLDVDA

>HvCML68

MGNASSMLTQYDIEEVQEHCSYLFSQQEIVSLYERFCQLDRSAKGFVSEDEFLSIPEFSTNPLSQRLLRMVDGLNFKEFVSFLSTFSARASLQQKIELIFKVYDVDGKGKVSFKDLVEVLRDLTGSSMSEKQREQVLTKVLEEAGYTQDSTLAIEDFVTIIDHPGLKMEVEVPID

>HvCML69

MQIERPKAENMSSRVDDSELRKVFQMFDKNGDGQITKKELRESLKNLGIYIPEDEMDATMAKIDTNGDGCVDIEEFGLLYRSILDESEGPNGGNMGDEEEAMREAFCVFDQNGDGYITIEELRSVLASLGLKQGRTIEECRQMISKVDANGDGRVDFKEFKQMMRGGGFAAIGRS

>HvCML70

MADYNRYGHAHGQGYGQQGYGYAPSAPPAPTPSSSSSYGYAPSASSPYGYGYGRGGYPPPPMGGFGGAVAFPPGTHPDVERAFRAVDRDRSGSIDEGELQAALSGAYHRFSIRTVRLLIFLFSDASPRSRMGPAEFATLWNCLGQWRVVFDRYDRDRSGKIESNELREALRGLGYAVPPAVIDLLIANYNNGVSNRGALDFDNFVECGMVVKGLTEKFKENDTRHTGSAALSYDGFLSMVIPFIVP

>HvCML71

MAFMRYRALPQGEVTAEEFWAWLGQFDADHDGRISRDELQRALRSLNLWFASWKAREGVRSADANRDGAVGREEAGRLFAYAQRQLGGKITQLRSY

>HvCML72

MAFMRYRALPQGEVTAEEFWAWLGQFDADHDGRISRDELQRALRSLNLWFASWKAREGVQAADANRDGAVGREEAGRLFAYAQRQLGGKITQLGSY

>HvCML73

MADDMERIFKRFDTNGDGKISLSELTDALRTLGSTSADEVQRMMAEIDTDGDGFIDFSEFISFCNANPGLMKDVAKAF

>HvCML74

MDTRQSVASVVKPSLPTDGGAPATASFRLRNGSLNSVRLRRVFDLFDKNGDGEITVDELAQALDSLGLVADREGLAATVGAYVPEGAAGLRFQDFESLHRELGDALFGALDDVPEDGEAGAGGDEEEMKEAFKVFDVDGDGFISASELQEVLKKLGLPEGGSLATVRQMICNVDRNSDGRVDFGEFKCMMKGITVWGA

>HvCML75

MAGKELSEEQVASMREAFSLFDTDGDGRIAPSELGVLMRSLGGNPTQAQLRDIAAQEKLTAPFDFPRFLDLMRAHLRPEPFDRPLRDAFRVLDKDASGTVSVADLRHVLTSIGEKLEPHEFDEWIREVDVAADGTIRYDDFIRRIVAK

>HvCML76

MGGVFGRHDAGRQSSHGMKLESKMVESMKQRASHGTSVKSFNSIIMKFAKIDKGLRKCKTIFQQFDEDSNGEIDKEELKNCFQKLEISFTEEEISDLFEACDINEDMGMKYNEFIVFLCLVYLLNDPAASEAKTKMGLGDLESTFETLVDAFVFLDKNKDGYVSKDEMIQAINESIPGERSAGRIAMKRFEEMDWDKNGMVTFKEFLFAFTRWVGIGENEDEDE

>HvCML77

MATPGRRPSAQQQPQPLTVDFEALSYISRLVEAFQAFDSDNDGLVTAPELRGLLASLGLDKSEAEARDMLARADADRDGRLCVEEFLDVMNAGELGLGALGELLQAAVPALESFAGPDGALGADELARVLGLMGTASAEDCAEIIACMDGDGDGAISVEEFKLMADLL

>HvCML78

MASYFRGQRLEPSSDAPRPGYRRDRGGRKRLTAQKRKEIKEAFDLFDTDGSGTIDPRELNVAMRALGFEMTPEQIQQMIAEVDKDGSGTIDLDEFIHMMTDKMGERDARDELHKAFRIIDQDANGKISDMDIQRLAIEAGEHFTLDEVREMIEAADENGDGEVDMEEFMKMMKRTGFGAGF

>HvCML79

MVVPSVFAAFDEDGDGKVSVSELRRCVEATLGEDVSEEEAASVLAAADADGDGLLNQEEFSRLAAGGAQEEDDADVKRRCLREAFGMYASSSAEDTATMITPASLRRTLSRLGSHELGVEECRAMICRFDLDGDGKLSFDEFRVMMMT

>HvCML80

MVVPSVFAAFDEDGDGKVSVSELRRCMEATLGADVSDEEAAVILASVDADGDGLLNQEEFSRLAAGAHEEDDADVKLRCLREAFGMYASSSAEDTATMITPASLRRTLSRLGSHELGVEECRAMICRFDLDGDGKLSFDEFRVMMMA
