# Supplementary figures and images for: Calmodulin and calmodulin-like gene family in barley: Identification, characterization and expression analyses
Source: Front Plant Sci. 2022 Aug 19;13:964888. doi: 10.3389/fpls.2022.964888 (PMC9439640; doi:10.3389/fpls.2022.964888)

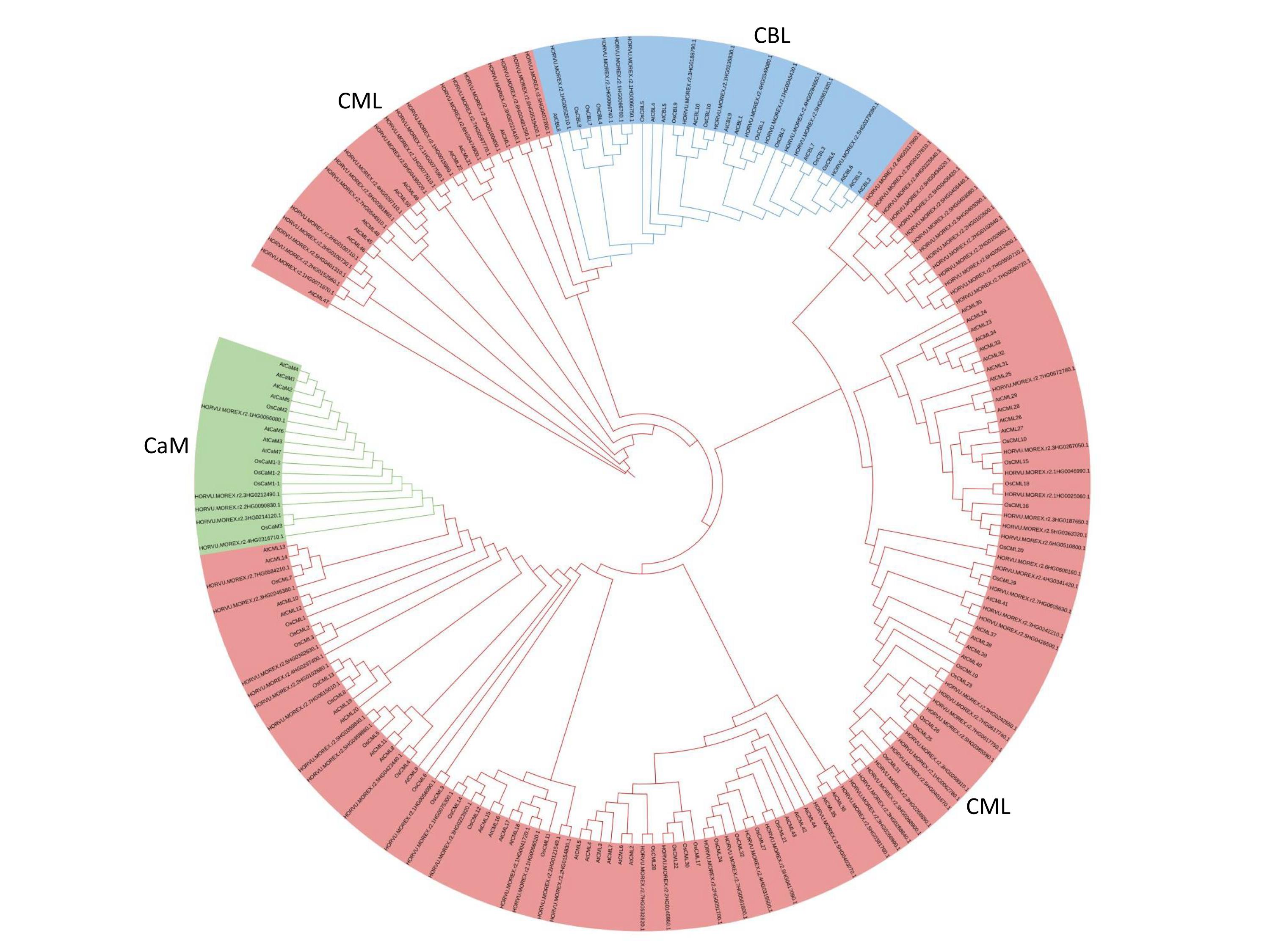


Phylogenetic analysis of calcium relay proteins in *Arabidopsis*, rice and barley.

Supplement: Supplementary file 8 [file Table_8.docx]
